# Spatiotemporal variability analysis of nutrient and sediment loads in surface water using improved hydrologic modeling for depression-dominated watersheds

**DOI:** 10.1007/s10661-026-15119-1

**Published:** 2026-03-05

**Authors:** Mosammat Mustari Khanaum, Marinus L. Otte, Xuefeng Chu

**Affiliations:** 1 Department of Civil, Construction and Environmental Engineering (Dept. 2470), North Dakota State University, PO Box 6050, Fargo, ND 58108-6050 USA; 2 Wet Ecosystem Research Group, Department of Biological Sciences (Dept. 2715), North Dakota State University, PO Box 6050, Fargo, ND 58108-6050 USA

**Keywords:** Surface depressions, Water quality, Watershed modeling, Overland flow generation, Spatial autocorrelation, Geodetector

## Abstract

Identifying pollutant hotspots and key influencing factors is crucial for mitigating nutrient and sediment pollution, which often involves collinearity among multiple independent variables. Spatial autocorrelation and Geodetector help address this collinearity challenge. The objective of this study was to examine the spatiotemporal variability of nutrients and sediment hotspots and identify the key driving factors. A recently developed Dynamic Partial Contributing Area (DPCA) model was coupled with the Soil and Water Assessment Tool (SWAT) and applied to a watershed in North Dakota, USA. Simulation results were used as inputs for Moran’s *I* spatial autocorrelation test to explore spatiotemporal variations of nutrients (total nitrogen (TN) and total phosphorus (TP)) and sediment hotspots. Additionally, Geodetector was employed to identify the key factors inducing these pollutions. Flood frequency analysis based on the 90-year peak discharge dataset was performed to observe variations in hotspots. The spatial autocorrelation results indicate a stronger correlation during dry periods compared to wet periods. Flood frequency analysis further underscored these findings, revealing that hotspots were more pronounced during periods of lower flood frequency. The Geodetector results showed that the interaction of precipitation and land use land cover (LULC) is the key indicator responsible for TN and TP pollution during wet periods, while the interaction of precipitation and cropping management factor emerges as the most significant influencing factor for sediment pollution during wet periods. This study demonstrates effective methods for identifying pollutant hotspots and their key driving factors, which will be helpful as theoretical guidance for best management practices (BMPs) for nutrient and sediment pollution.

**Supplementary Information:**

The online version contains supplementary material available at 10.1007/s10661-026-15119-1.

## Introduction

Statistical techniques such as multivariate tests, regression analysis, principal component analysis, clustering, and decision trees are commonly used for water quality analysis. However, inherent collinearity (multicollinearity) poses a challenge for statistical methods and its inferential interpretation, often leading to the exclusion of key environmental factors (Graham, 2003; Shi et al., 2022). Moreover, identifying and interpreting spatial variations of non-point source (NPS) pollutants and their influencing factors can be difficult. In contrast, spatial autocorrelation and Geodetector (Wang et al., 2010) help address this collinearity challenge. Geodetector has been applied across various research fields, including epidemiology and public health (Wang et al., 2010), environmental science and ecology (Matomela et al., 2022; Shi et al., 2022), land management (Shen et al., 2022), as well as economics and social sciences. Spatial autocorrelation has also been applied in various fields, including epidemiology and public health (Park et al., 2022), environmental science and ecology (Xie et al., 2024), and land management (Shen et al., 2022; Zhang et al., 2023) to quantify and analyze spatially stratified heterogeneity, helping researchers detect patterns where neighboring areas exhibit similar or different characteristics at various scales.

In the area of water resources, Shrestha and Luo (2017) used Geodetector to identify key factors of elevated nitrate concentrations in groundwater, and this tool has also been used to identify the main factors affecting surface water quality (Shi, 2023; Shi et al., 2022). Using hydrological simulations, Geodetector, and redundancy analysis, Shi et al. (2022) assessed how natural geographic features and landscape patterns influenced NPS pollution and found that proportions of agricultural land, forest area, and landscape contrast index were key factors influencing nutrient levels and the interactions between these factors significantly affected NPS pollution. Gao et al. (2022) used spatial autocorrelation to investigate the temporal and spatial characteristics of river water quality, determining water quality trends over time, identifying high nutrient concentration clusters, and observing variations in nutrient concentrations across different seasons. Using nutrients and sediment results simulated by the SWAT model, some studies used spatial autocorrelation to identify NPS pollutant clusters across watersheds (Shen et al., 2022; Wang et al., 2023; Zhang et al., 2023). Similarly, this method has been used to analyze the spatial and temporal distributions of total nitrogen (TN) and total phosphorus (TP) loads within watersheds (Wang et al., 2023) as well as identify trends of nutrient inputs and outputs (Shen et al., 2022).

In traditional watershed models, surface depressions are often filled to create a continuous drainage network with hydrologically connected areas. However, due to having unique filling-spilling characteristics, surface depressions influence overland flow within a watershed and perform critical sink‐lag‐source functions affecting downstream waters (Lane et al., 2018). Upstream water is stored in depressions, which leads to a delayed initiation of surface runoff at the onset of a rainfall event and provides an extended residence time for contaminant transport. At that stage, surface depression can function in two different ways: it may act as a sink by storing water for extended periods, serve as a gatekeeper by temporarily holding water and releasing it later, or operate as a source by routing water through processes such as evapotranspiration, seepage, and overland flow ultimately contributing outflow to downstream areas (Rajib et al., 2020; Tahmasebi Nasab et al., 2018).

Like hydrologic models, water quality models rely on mathematical equations of mass balance coupled with flow models to estimate the concentration of dissolved and suspended pollutants in streamflow. Contaminants are washed off the ground surface by runoff, entering stream channels as distributed inputs and transporting downstream with water flow. The initial availability of contaminants, the fraction washed off during a rainfall event, and the residence/retention time are critical factors in water quality simulation. As pollutants travel and are detained in surface depressions, their mass may be reduced through various environmental attenuation processes, which influence the overall transport of the contaminants (Putz et al., 2003). Hence, there would be potential consequences of not incorporating depression filling-spilling characteristics into watershed modeling.

Incorporating the complex characteristics of surface depressions into hydrologic and water quality modeling is challenging because the contributing area of a depression varies across spatial and temporal scales (Khanaum et al., 2025) and the associated overland flow generation exhibits a hierarchical pattern. Consequently, excluding or oversimplifying surface depressions in a model makes it inappropriate to represent the real and complex hydrologic and water quality systems of an area such as the Prairie Pothole Region (Qi et al., 2023; Tahmasebi Nasab et al., 2018).

Several studies have endeavored to account for the intrinsic filling-spilling characteristics of surface depressions in hydrologic modeling (e.g., Callaghan & Wickert, 2019; Evenson et al., 2016; Green & Crumpton, 2023; Haque et al., 2018; Khanaum et al., 2025; Muhammad et al., 2019; Shook et al., 2021; Zeng & Chu, 2021a; Zeng & Chu, 2021b). A new model coupling the Dynamic Partial Contributing Area (DPCA) method with the Soil and Water Assessment Tool (SWAT), DPCA-SWAT (Khanaum et al., 2025), accounted for flow dynamics of surface depressions to improve hydrologic modeling for depression-dominated watersheds. The DPCA-SWAT model allows separate runoff simulations for depressional and non-depressional areas in a watershed, accounting for the dynamic variations in contributing areas, ponded areas, storages, and overflow of all depressions, while also characterizing the dynamics of overland flow generation influenced by surface depressions to better reflect the complex reality (Khanaum et al., 2025). This study aimed to identify pollutant hotspots and their key driving factors. To achieve this, the DPCA-SWAT model was employed for hydrologic and water quality modeling, and the results were utilized to (1) identify nutrients (TN and TP) and sediment hotspots using Moran’s *I* autocorrelation test and explore the spatiotemporal variations of those hotspots; (2) examine noticeable variations in hotspot characteristics during periods of lower/higher flood frequency through flood frequency analysis; and (3) identify key factor(s) responsible for these pollutions by Geodetector test.

## Materials and methods

### Study area

**Fig. 1:**
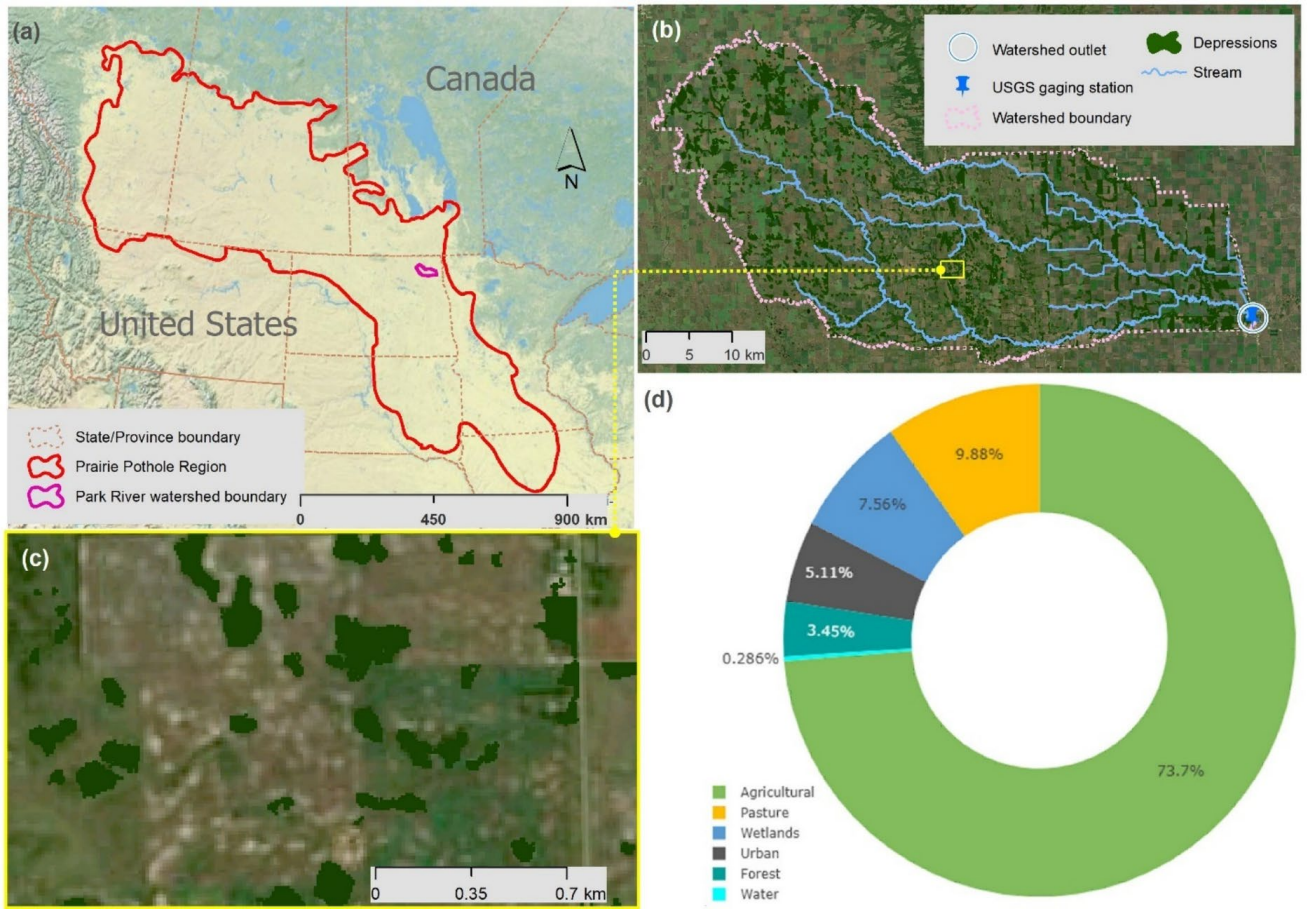
**a** Location of the Park River watershed within the Prairie Pothole Region; **b** USGS gaging station, streams, and depressions; **c** surface depressions within the watershed; and **d** distribution of land uses in the watershed

The Park River (PR) watershed in the Prairie Pothole Region (PPR) of North Dakota (ND) was selected for the study (Fig. 1a). Located in Walsh, Pembina, and Cavalier Counties in ND, the watershed features numerous surface depressions (Fig. 1b, c), which potentially affect surface runoff generation, contributing area (CA) dynamics, and water quality. The drainage area is 1446.4 km^2^, 22.96% of which is depressional area (i.e., 332.2 km^2^). The area is located in a cold climate region. During our study period, the lowest temperature was recorded as −17.68 °C and the highest temperature was 28.41 °C. The drainage area is identified by using the 8-digit Hydrologic Unit Code (HUC) 09020310. The terrain is relatively flat in relief, with slopes ranging from 0.04 to 0.3 m/km (Lin et al., 2015). Due to its flat topography, the area is prone to flooding, particularly from spring snowmelt. According to the 2019 Cropland Data Layer (USDA, 2024a), the dominant land use type in the study area is agricultural crops (73.7%), followed by pasture, wetlands, and urban areas (Fig. 1d). The final outlet of the watershed is at the USGS gaging station # 05090000 Park River at Grafton, ND (latitude: 48°25′29″ N, longitude: 97°24′42″ W). Watershed elevation ranges from 257 m in the southeast to 488 m in the west. The area is characterized by a climate primarily influenced by dynamic continental conditions, with an average yearly precipitation of 44.7 cm (Mushet et al., 2015). According to the classifications of the Soil Survey Geographic Database (SSURGO), the region contains 17 different soil types.

### Overall modeling framework

**Fig. 2:**
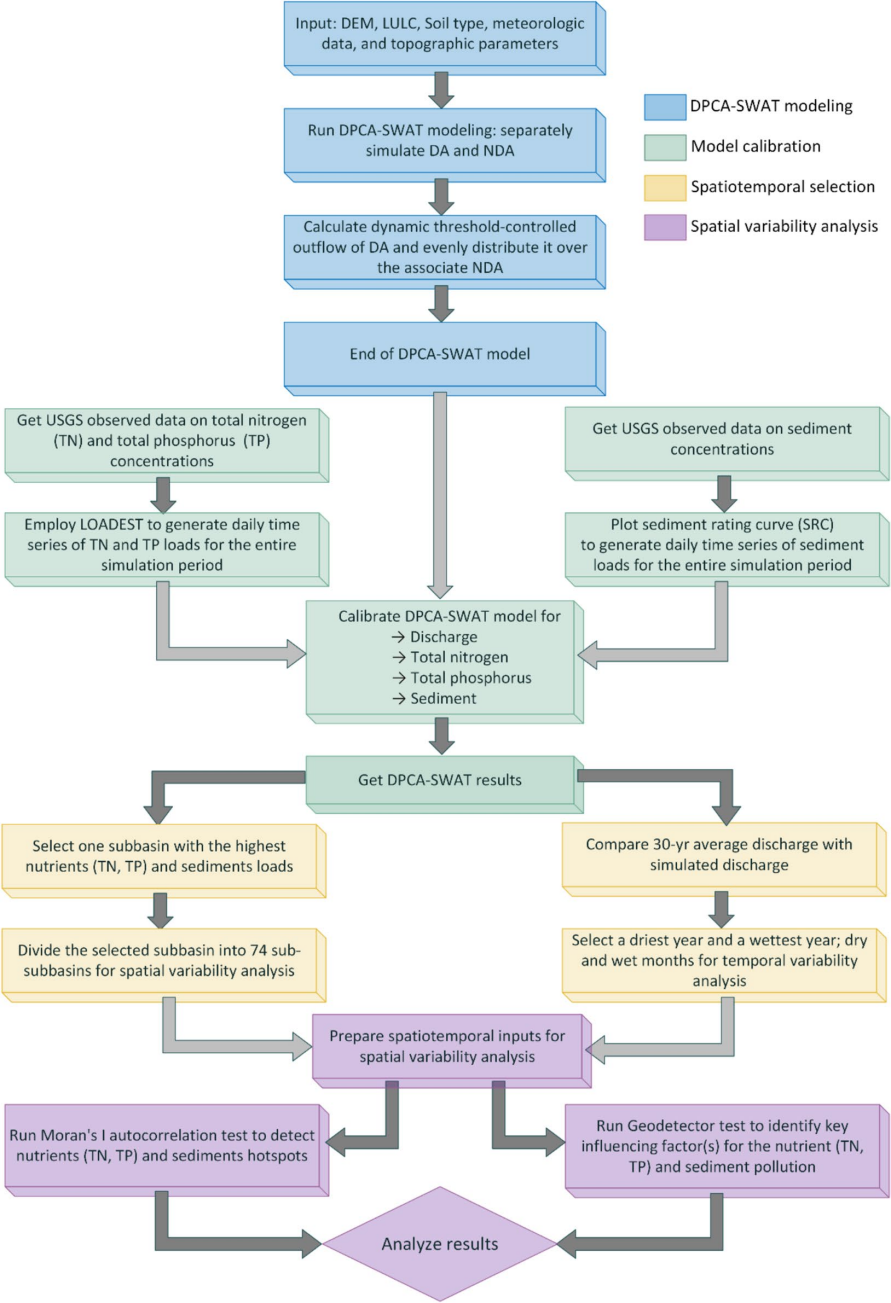
Detailed modeling and analysis framework (DEM, digital elevation model; LULC, land use land cover; DA, depressional area; NDA, non-depressional area; USGS, United States Geological Survey; LOADEST, load estimator model)

The overall modeling framework is illustrated in Fig. 2, consisting of four major steps: (1) DPCA-SWAT modeling (Khanaum et al., 2025); (2) model calibration and validation; (3) selection of the most polluted subbasin and different temporal settings; and (4) spatial variability analysis which is divided into two parts: pollutant hotspots detection with spatial autocorrelation and key influencing factor identification with Geodetector test. The DPCA-SWAT model separately simulates depressional areas (DAs) and non-depressional areas (NDAs) and calculates the threshold-controlled overflow from DAs at each time step, thereby distributing the water over the associated NDA as excess surface runoff. The model was calibrated and validated for discharge, nutrients (TN, TP), and sediment at the outlet of the watershed. No additional flow or water quality monitoring stations were available within the watershed. Due to the lack of sufficient observed water quality data at the watershed outlet, a sediment rating curve was developed to generate a sediment time series. In addition, LOADEST developed by the USGS (Runkel et al., 2004) was employed to get the TN and TP time series.

Next, the driest year, the wettest year, as well as dry and wet months were identified for spatial variability analysis. Based on the DPCA-SWAT modeling results, the highest polluted subbasin was selected for spatiotemporal analysis. Spatial autocorrelation was conducted to detect pollutant hotspots in different temporal settings. Finally, the key factors responsible for those nutrients and sediment pollutions were identified by the Geodetector test (Wang et al., 2010). The study used a 10-m digital elevation model (DEM) downloaded from the USGS National Map Viewer (USGS, 2024a). The soil type dataset was obtained from the Soil Survey Geographic Database (SSURGO) (USDA, 2024b), and the land use dataset was downloaded from the Cropland Data Layer (USDA, 2024a). In addition, daily precipitation and maximum and minimum temperatures were downloaded from the World Weather for Water Data Service W3S platform (Ghimire et al., 2022).

### DPCA-SWAT model

The DPCA-SWAT model (Khanaum et al., 2025) incorporates the filling-spilling processes of surface depressions. In the DPCA modeling framework, each subbasin is divided into DA and NDA, which are simulated separately. The DPCA-SWAT modeling approach involves four main steps: delineating the watershed and subbasin boundaries; extracting topographic parameters required for the DPCA algorithm; performing the DPCA modeling for DAs; and finally, based on the DPCA output, conducting the DPCA-SWAT modeling for the entire watershed (Khanaum et al., 2025). In the SWAT modeling, the area corresponding to DA is excluded so that it only simulates surface runoff for the NDA. The DPCA component captures temporal changes in surface depressions, estimating contributing areas, storage capacity, and water release from depressions at each time step. The water released from all depressions within a subbasin is aggregated and added to the SWAT model by evenly distributing the released water over the subbasin as additional equivalent rainfall excess. Afterward, the channel routing process is performed by SWAT.

To determine water release from surface depressions, dynamic changes in water balance are simulated for each individual depression, assuming that a depression does not release water until it is completely filled. The ponded water released from depressions is then incorporated into the SWAT modeling for the corresponding subbasins. The DPCA model calculates water released from a depression under two conditions: (1) if the ponded storage of a depression is less than or equal to its storage capacity, no water is released; (2) if the ponded storage of a depression is greater than its storage capacity, the model calculates the water release as the difference between the ponded storage at a given time step and the storage capacity of the depression (Khanaum et al., 2025). The outflow from a depression can be expressed as (Khanaum et al., 2025):

1 $${DO}_{i,j,k}=\left\{\begin{array}{cc}0&for\;S_{i,j,k}\leq S_{i,j}^{mx}\\\left(S_{i,j,k}-S_{i,j}^{mx}\right)&for\;S_{i,j,k}>\,S_{i,j}^{mx}\end{array}\right.$$

in which

2 $$S_{\textit{i},\textit{j},\textit{k}}=S_{\textit{i},\textit{j},\textit{k}-1}+\left({PA}_{\textit{i},\textit{j},\textit{k}-1}\times P_{\textit{i},\textit{j},\textit{k}}\right)+\left({CA}_{\textit{i},\textit{j},\textit{k}-1}\times R_{\textit{i},\textit{j},\textit{k}}\right)-{EV}_{\textit{i},\textit{j},\textit{k}}-{PR}_{\textit{i},\textit{j},\textit{k}}$$

where ${DO}_{\mathrm{i},\mathrm{j},\mathrm{k}}$ is the outflow from surface depression* j* in subbasin* i* during time step* k* [L^3^]; ${S}_{\mathrm{i},\mathrm{j},\mathrm{k}}$ and ${S}_{\mathrm{i},\mathrm{j},\mathrm{k}-1}$ are the storage volume of surface depression* j* in subbasin* i* at time step* k* and *k-1* [L^3^]; ${S}_{\mathrm{i},\mathrm{j}}^{\mathrm{mx}}$ is the maximum storage capacity of surface depression* j* in subbasin* i* [L^3^]; ${PA}_{\mathrm{i},\mathrm{j},\mathrm{k}-1}$ is the ponding area of depression* j* in surface subbasin* i* at time step *k-1* [L^2^]; ${PCP}_{\mathrm{i},\mathrm{j},\mathrm{k}}$ is the precipitation over surface depression* j* in subbasin* i* during time step* k* [L]; ${CA}_{\mathrm{i},\mathrm{j},\mathrm{k}-1}$ is the contributing area of surface depression *j* in subbasin* i* at time step *k-1* [L^2^]; ${R}_{\mathrm{i},\mathrm{j},\mathrm{k}}$ is the surface runoff generated from the contributing area of surface depression *j* in subbasin* i* during time step* k* [L]; ${EV}_{\mathrm{i},\mathrm{j},\mathrm{k}}$ is the volume of water loss through evaporation from depression* j* in subbasin* i* during time step* k* [L^3^]; and ${PR}_{\mathrm{i},\mathrm{j},\mathrm{k}}$ is the percolation loss of depression* j* in subbasin* i* during time step* k* [L^3^] (Khanaum et al., 2025).

Three additional topographic parameters for each surface depression, including ponded area (PA), contributing area (CA), and ponded water volume (PS), were required for the DPCA-SWAT model. Following Qi et al. (2023) and Khanaum et al. (2023), these parameters were calculated by employing the Hydrologic Unit Delineation for Depressions and Channels (HUD-DC, Wang & Chu, 2019). HUD-DC, an ArcGIS-based program developed for depression-oriented delineation (Chu et al., 2013; Chu, 2017), was added to ArcMap as part of the ArcToolbox. Using a DEM, it calculates the maximum surface area, maximum storage volume, contributing area of each depression, and other topographic parameters. In this study, the DPCA-SWAT model was applied to the PR watershed. SWAT-CUP (version 5.2.1.1, Abbaspour et al., 2017) was utilized for model calibration and validation for flow, nutrients (TN and TP), and sediment using the observed data downloaded from the USGS National Water Information System (USGS, 2024b). The calibration period ranged from 2011 to 2014 and the validation period from 2015 to 2018, with a 3-year warm-up period from 2008 to 2010. This warm-up period allows important processes (state variables) of a model to reach an equilibrium stage.

### Water quality calibration

Although the observed discharge data for the entire simulation period (2922 days from 2011 to 2018) were available, the observed water quality data (TN, TP, and sediment loads) were available for only 64 days. These water quality monitoring data were typically collected at lower frequencies (monthly or as few as 5–8 grab samples per year) compared to streamflow data. Additionally, grab samples can be affected by various factors such as uneven depth, collection methods, sampling frequency, and natural disturbances (Heng & Suetsugi, 2014). Among those limited grab samples, a few were not consistent with discharge. To address these challenges, the study employed two methods: sediment rating curves (SRC) and the USGS Load Estimator (LOADEST) program (Runkel et al., 2004). The time series of daily TN, TP, and sediment loads for the entire 2922-day period were created using the LOADEST and SRC to calibrate the water quality model at the outlet of the PR watershed and to assess the model performance.

#### Sediment rating curve

The sediment rating curve (SRC) method has been used for establishing the relationship between water discharge and sediment load (Isik, 2013). SRC has been widely used as a practical method to estimate sediment loads, particularly for data-scarce watersheds (Horowitz, 2003; Lee & Lee, 2010). Due to its simplicity and effectiveness, it remains a popular approach for generating sediment load time series. Thus, we used this method to generate the sediment load time series in this study. Specifically, a linear equation is fitted to the observed datasets of log(sediment load) and log(discharge) as follows:

3 $$\mathit{log}\left(Y\right)=\mathit{log}\left(a\right)+b\times log(Q)$$

where *Y* = sediment load (ton/day); *Q* = discharge (m^3^/s); and *a* and *b* are two coefficients. The daily sediment load dataset generated by Eq. 3 was used for model calibration and validation.

#### Load estimator (LOADEST)

The USGS LOADEST (Load Estimator) is a FORTRAN program for estimating pollutant loads over continuous time intervals (daily, monthly, or seasonal) using a set of regression models (Runkel et al., 2004). Those regression models incorporate explanatory variables such as streamflow functions, decimal time, and other user-defined variables (Runkel et al., 2004). LOADEST consists of 11 regression models, and the program can automatically select the best-fitting model that effectively represents the relationship between streamflow and water quality to estimate instantaneous pollutant loads. To estimate model coefficients, LOADEST provides three calibration methods based on model residuals distribution and data censoring: maximum likelihood estimation (MLE), adjusted maximum likelihood estimation (AMLE), and least absolute deviation (LAD) (Runkel et al., 2004). Among them, MLE and AMLE are applied when the residuals follow a normal distribution, while LAD is used when the model residuals do not meet normal distribution criteria (Runkel et al., 2004). LOADEST has been used for generating continuous data from discrete measurements and for estimating daily, monthly, or seasonal pollutant loads (Ji & Lu, 2018; Kim et al., 2018; Noori et al., 2020; Shrestha et al., 2018; Wallace et al., 2018; Wang et al., 2016). TN and TP loads are influenced by a broader range of factors. LOADEST is better equipped to capture the related complex relationships and has been widely used for TN and TP load estimation. In this study, LOADEST was used to generate the daily time series of TN and TP loads aligned with the daily discharge data from 2011 to 2018, which were further utilized for water quality model calibration and validation.

### Spatiotemporal variability analysis

For spatial variability analysis, the nutrients and sediment loads simulated by the DPCA-SWAT model were used to identify the subbasin with the highest nutrient and sediment loads. The selected subbasin was further divided into smaller areas (hereafter called sub-subbasins). In addition to spatial analysis, the study also examined temporal variations in nutrient and sediment loads. The driest year and the wettest year in the dataset were selected by comparing with the 30-year (1994–2023) average annual discharges at the watershed outlet. Using the 90-year discharge data, a flood frequency analysis was conducted to highlight variation in hotspot characteristics. To enhance understanding of how hydroclimatic conditions influence water quality, dry and wet months were further identified for a more detailed temporal variance analysis, by comparing monthly average discharge data against the 30-year average monthly discharge at the watershed outlet. Then, spatial autocorrelation and the Geodetector test were performed to locate hotspots, explore spatiotemporal variability, and identify the key influencing factors of pollution hotspots.

#### Spatial autocorrelation with Moran’s index

Moran’s index (Moran’s *I*) is an inferential statistic to measure spatial autocorrelation based on both feature locations and values. The results are interpreted within the context of a null hypothesis of spatial randomness in favor of identifying the presence of some type of spatial pattern (Anselin, 2024). Given a set of features and the associated attributes, Moran’s *I*, *Z*-scores (standard deviation), and *p*-values (probability) are calculated and used to evaluate whether a pattern is clustered, dispersed, or random. A small *p*-value and an extreme *Z*-score (either high or low) at the tails of the normal distribution curve indicate statistically significant data. A positive Moran’s *I* suggests a tendency toward clustering, while a negative value indicates dispersion. Here, spatiotemporal correlations of nutrients (TN, TP) and sediment were identified via Moran’s *I* autocorrelation. A weight matrix (*W*) was determined following Shen et al. (2022), based on inverse distance and minimum bandwidth. We utilized the open-source software Geoda V1.22.0.4 (Anselin, 2024) to identify local clusters and spatial outliers using five patterns: “high-high,” “high-low,” “low–high,” “low-low,” and “not significant” (*p* > 0.05). Among these, “high-high” indicates a cluster of high values surrounded by other high values, while “high-low” refers to a cluster of high values surrounded by low values (Anselin, 2024), with “high-high” clusters considered more important because they indicate spatial locations with high loading of nutrients and sediment. In this study, “high-high” clusters (Moran’s *I* > 0 and *p* < 0.05) are termed as “hotspots,” which refer to the areas with a statistically significant elevated prevalence of pollutant loadings.

#### Geodetector for identifying key factors responsible for nutrients and sediment pollution

Geodetector quantifies spatial variance based on the degree of influence of a single or multiple factors (independent variables) on a dependent variable (Wang et al., 2010). The tool calculates the ability of independent variables (*q*) to influence a dependent variable and also assesses the interaction between two factors (*X*_1_ and *X*_2_) across five different relationships: weaken-nonlinear, weaken-unifactor, enhance-bifactor, independent, and enhance-nonlinear. Specifically, if *q*(*X*_1_ ∩ *X*_2_) < Min(*q*(*X*_1_), *q*(*X*_2_)), it is termed weaken-nonlinear, while *q*(*X*_1_ ∩ *X*_2_) > *q*(*X*_1_) + *q*(*X*_2_) is referred to as enhance-nonlinear (Wang et al., 2010). Similarly, enhance-bifactor refers to where the interaction between two factors has a greater influence than either factor alone, while weaken-unifactor occurs when the combined influence of two factors is greater than the weaker one but less than the stronger one, indicating that their interaction adds some explanatory power but does not exceed that of the stronger individual factor. In this study, independent variables (e.g., LULC) and dependent variables (e.g., TN) were acquired from the DPCA-SWAT model, and they were pre-processed at the sub-subbasin level. For conducting the Geodetector test, it is essential to have spatially explicit values for all potential influencing factors across all classification categories in our study sub-subbasins. Obtaining these data from external sources for all sub-subbasins is challenging and may introduce additional uncertainties. Therefore, to ensure consistency with the modeling framework and maintain the spatial resolution of the study, we extracted the necessary factor values from the DPCA-SWAT input files. This approach allows us to preserve alignment between the input data used in the simulation and the data used in Geodetector analysis.

Geodetector identifies spatial variations in a dependent variable and clarifies how different influencing factors affect these variations. This is achieved by comparing the intra-layer variance of one variable and the inter-layer variance between two or more independent variables using the following equation (Wang et al., 2010):

4 $$q=1-\frac{\sum_{h=1}^L\;N_h\sigma_h^2}{N\sigma^2}$$

where *q* = the influence strength of independent variable/factor (e.g., LULC) on a dependent variable (e.g., TN load); *h* = classification categories of the independent variables (1, 2, 3, …, *L*), representing the types of LULC in different sub-subbasins in this study; ${N}_{\mathrm{h}}$ = number of units within category *h* (e.g., seven types of LULC in a sub-subbasin); *N* = total number of units across all categories in the region; ${\sigma }_{\mathrm{h}}^{2}=$ variance of the dependent variable within category *h*; and ${\sigma }^{2}=$ variance of the dependent variable in the entire region. The value of *q* ranges from 0 to 1, indicating the spatial heterogeneity of the factor (in this study, LULC) and the extent to which it explains the spatial variation of TN load. A value of *q* close to 1 indicates stronger explanatory power of LULC, while a value of 0 indicates that TN load is completely unrelated to LULC. For interaction detection, *q* evaluates the explanatory ability of two or more factors when they interact simultaneously. For example, assume the influence strength of factor *X*_1_, *q*(*X*_1_) = 0.5 and factor *X*_2_, *q*(*X*_2_) = 0.2, with the influence strength of their interaction, *q*(*X*_1_ ∩ *X*_2_) = 0.8. In this case, following Wang et al. (2010), it indicates an enhanced-nonlinear relationship since *q*(*X*_1_ ∩ *X*_2_) > *q*(*X*_1_) + *q*(*X*_2_).

Geodetector was employed to identify the key factor(s) influencing nutrients (TN and TP) and sediment loads in different temporal settings. Four factors (independent variables) were selected, including precipitation, LULC, land cover and management factor for the universal soil loss equation (USLE_C), and leaf area index (LAI). In selecting factors, it was considered that they significantly impact the three selected pollutants. For example, the USLE_C factor reflects the effect of cropping and management practices, including crop rotations, prior land use, canopy cover, surface roughness, soil moisture, as well as the related management practices such as weed control, tillage, watering, fertilization, and crop residues. Precipitation, being a major driving force of hydrological processes as well as sediment and nutrient fluxes (Price et al., 2014; Tuo et al., 2016), was included as a key variable in the Geodetector analysis.

The Geodetector analysis was performed to better capture the influencing factors driving pollutant hotspots variability within the most polluted subbasin. In this study, the DPCA-SWAT model was calibrated for the Park River watershed, and the most polluted subbasin (Subbasin 5) was identified for subsequent spatial autocorrelation and Geodetector analyses. Subbasin 5 was further divided into 74 smaller areas using ArcSWAT. The calibrated DPCA-SWAT parameters were then applied to re-run the DPCA-SWAT model, and the required subbasin-level inputs for the Geodetector analysis were extracted from the database. At this point, there were 74 subbasins, which are herein referred to as “sub-subbasins.” The SWAT model typically uses data from the precipitation gauge station closest to the centroid of each subbasin, which is subsequently adjusted using the elevation band method (Tuo et al., 2016). Before running the DPCA-SWAT model, the precipitation data from 28 locations were collected to better represent spatial variability within the Park River watershed. Among these, three stations were located within the most polluted subbasin (Subbasin 5), and three additional stations were in close proximity. Consequently, when DPCA-SWAT further divided this polluted subbasin into several sub-subbasins, the data from these six weather stations were used to generate spatially varying precipitation inputs for each sub-subbasin. Although we use the term “sub-subbasins,” these units correspond to the subbasin-level data structure within the model. Therefore, all 74 sub-subbasins are characterized by spatially heterogeneous precipitation, LULC, slope, pollutant loads, and other variables. Subsequently, the influence of each individual indicator and the interactions among indicators were analyzed to identify the key factors contributing to nutrients and sediment pollution. All results were examined, but only enhanced nonlinear interactions were considered in this study. The significance of the *q*-value was determined by an *F*-test with a significance level of 0.05.

## Results and discussions

### Characteristics of surface topography

**Fig. 3:**
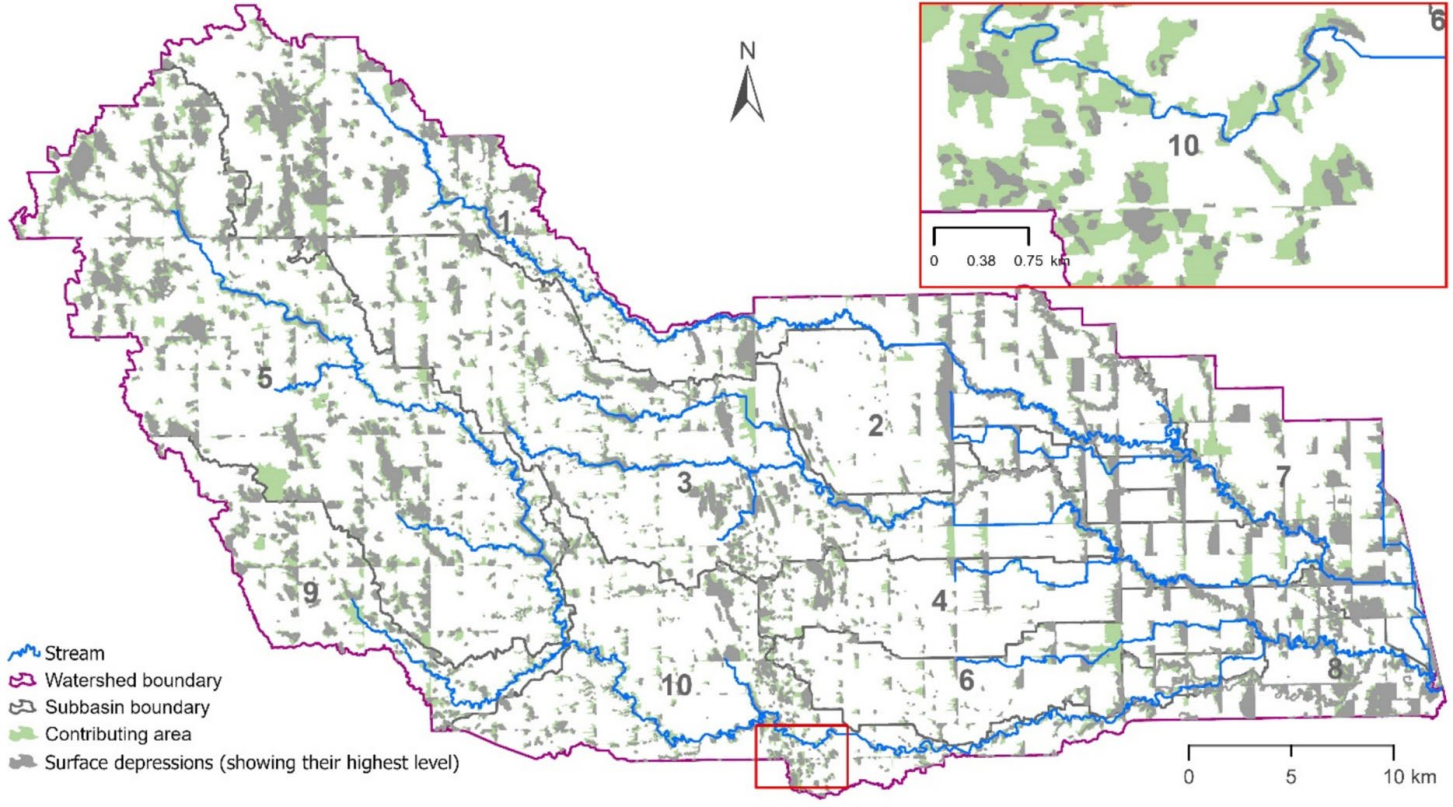
Distribution of the highest level of surface depressions and contributing areas (CAs) across subbasins in the Park River watershed, with inset figure showing the details of a small area of subbasin 10

Throughout the PR watershed, there are numerous unevenly distributed depressions (Fig. 3). A total of 5725 surface depressions were identified, and their topographic parameters were calculated by using HUD-DC. The sizes and spatial distributions of these surface depressions across the subbasins differed significantly (Fig. 3 and Table 1). The total contributing area (CA) of the depressional area (DA) was 214.65 km^2^, ponded area (PA) was 117.50 km^2^, and the storage capacity (PS_mx_) was 69.26 $\times$ 10^6^ m^3^ (Table 1). The subbasin-level depressional area (DA) varied between 14.51 and 35.23% of the associated subbasin areas, indicating varying effects of depressions on surface runoff generation.

**Table 1:** Topographic parameters across all subbasins in the PR watershed

Sub	Sub area (km^2^)	Non-depressional area (NDA)		Depressional area (DA)					
		Area (km^2^)	NDA (%)	Area (km^2^)	DA (%)	No. of depressions	∑CA (km^2^)	∑PA_mx_ (km^2^)	∑PS_mx_ (10^6^ m^3^)
1	239.99	175.99	73.33	64.01	26.67	1097	40.34	23.66	12.14
2	82.62	70.63	85.49	11.99	14.51	127	5.80	6.18	1.59
3	246.52	195.89	79.46	50.63	20.54	1226	34.30	16.32	20.45
4	98.33	81.95	83.35	16.37	16.65	225	9.49	6.89	3.37
5	311.52	232.62	74.67	78.90	25.33	859	51.82	27.07	18.63
6	85.92	71.97	83.76	13.96	16.24	200	8.51	5.44	1.82
7	95.02	67.87	71.42	27.15	28.58	236	14.90	12.25	2.01
8	52.04	33.70	64.77	18.33	35.23	302	11.31	7.03	2.21
9	72.53	55.67	76.75	16.87	23.25	283	11.80	5.07	1.69
10	161.86	127.91	79.03	33.95	20.97	1170	26.37	7.58	5.36
*Total*	*1446.35*	*1114.20*	*77.035*	*332.15*	*22.96*	*5725*	*214.65*	*117.50*	*69.26*

*Sub* subbasin, *CA* contributing area, *PA*_*mx*_ maximum ponded area, *PS*_*mx*_ storage capacity

### Processing of TN, TP, and sediment loading datasets

**Fig. 4:**
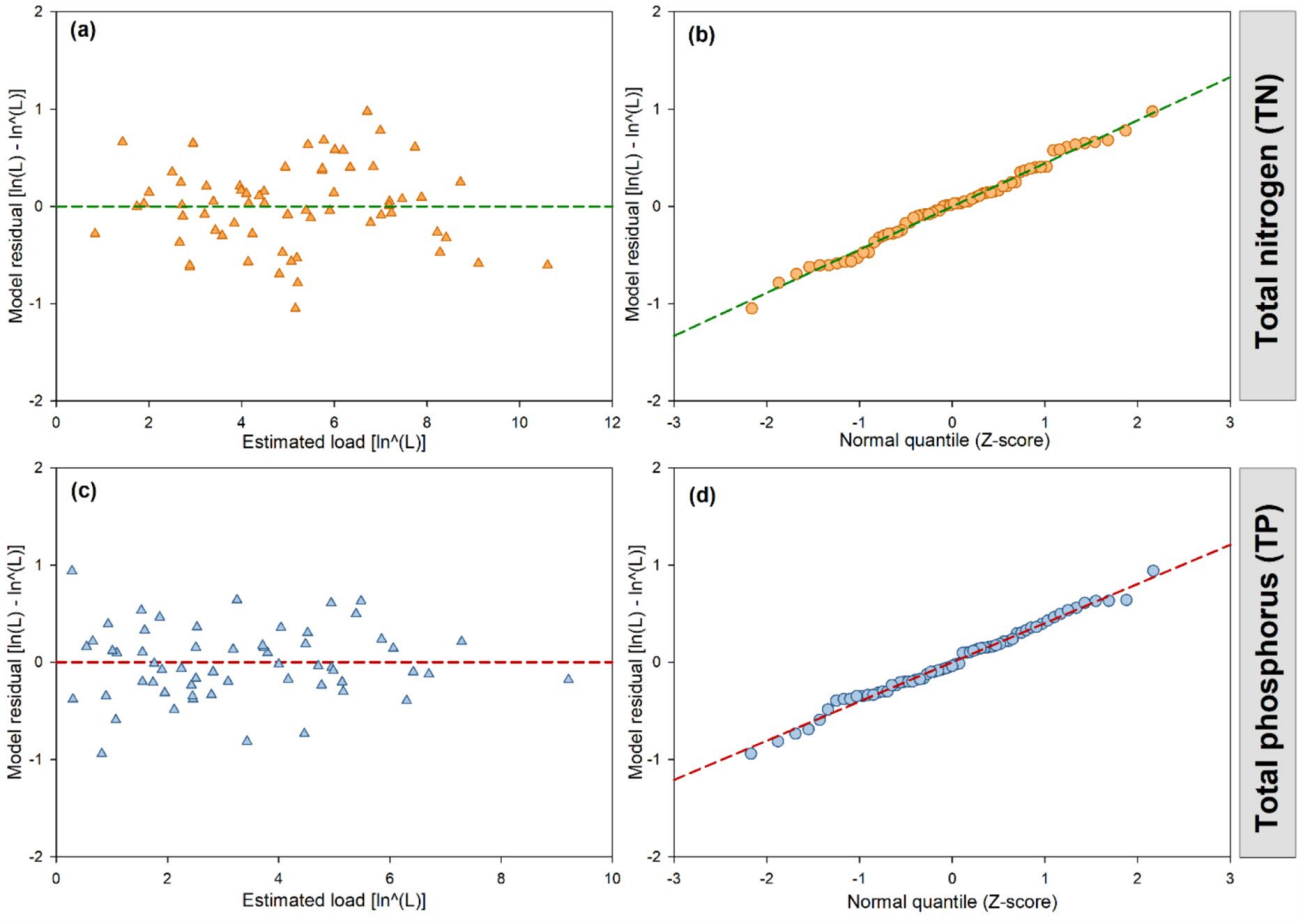
Adjusted maximum likelihood estimation (AMLE) regression **a** log load estimates vs. model residuals for total nitrogen; **b** normal probability plot of total nitrogen model residuals; **c** log load estimates vs. model residuals for total phosphorus; **d** normal probability plot of total phosphorus model residuals

For water quality model calibration purposes, the observed TN, TP, and sediment load data were processed using LOADEST and the SRC method, and the corresponding daily time series were generated. The AMLE method in LOADEST was selected for model calibration as the simulation residuals met the two assumptions of non-collinearity and normal distribution (Fig. 4). As shown in Fig. 4a, c, the residuals for both TN and TP loads were scattered randomly without patterns or trends, indicating they were independent and homoscedastic (i.e., having constant variance, Runkel et al., 2004). To test the second assumption, a normal probability plot was created using *Z*-scores and model residuals (Fig. 4b, d), and the linearity of the plot suggests that the residuals were normally distributed for both TN and TP. The program selected regression model 7 (Eq. 5) and model 6 (Eq. 6) to generate the daily time series of TN and TP loads for the simulation period:

5 $$\textit{Ln}\left(TN\right)=5.3242+1.0975\text{ }\textit{Ln}Q+0.3717\text{ }\textit{Sin}\left(2\pi\Delta t\right)+0.3038\textit{Cos}\left(2\pi\Delta t\right)-0.0623\;\Delta\textit{t}$$

6 $$\textit{Ln}\left(TP\right)=2.8543+1.2052\text{ }\textit{Ln}Q+0.0320\text{ }\textit{Ln}Q^2-0.2827\textit{Sin}\left(2\pi\Delta t\right)-0.0361\textit{Cos}\left(2\pi\Delta t\right)$$

where *TN* = total nitrogen load (kg/day); *TP* = total phosphorus load (kg/day); Ln*Q* = Ln(*Q*) − center of Ln(*Q*); and $\Delta t$ = decimal time − center of decimal time.

**Fig. 5:**
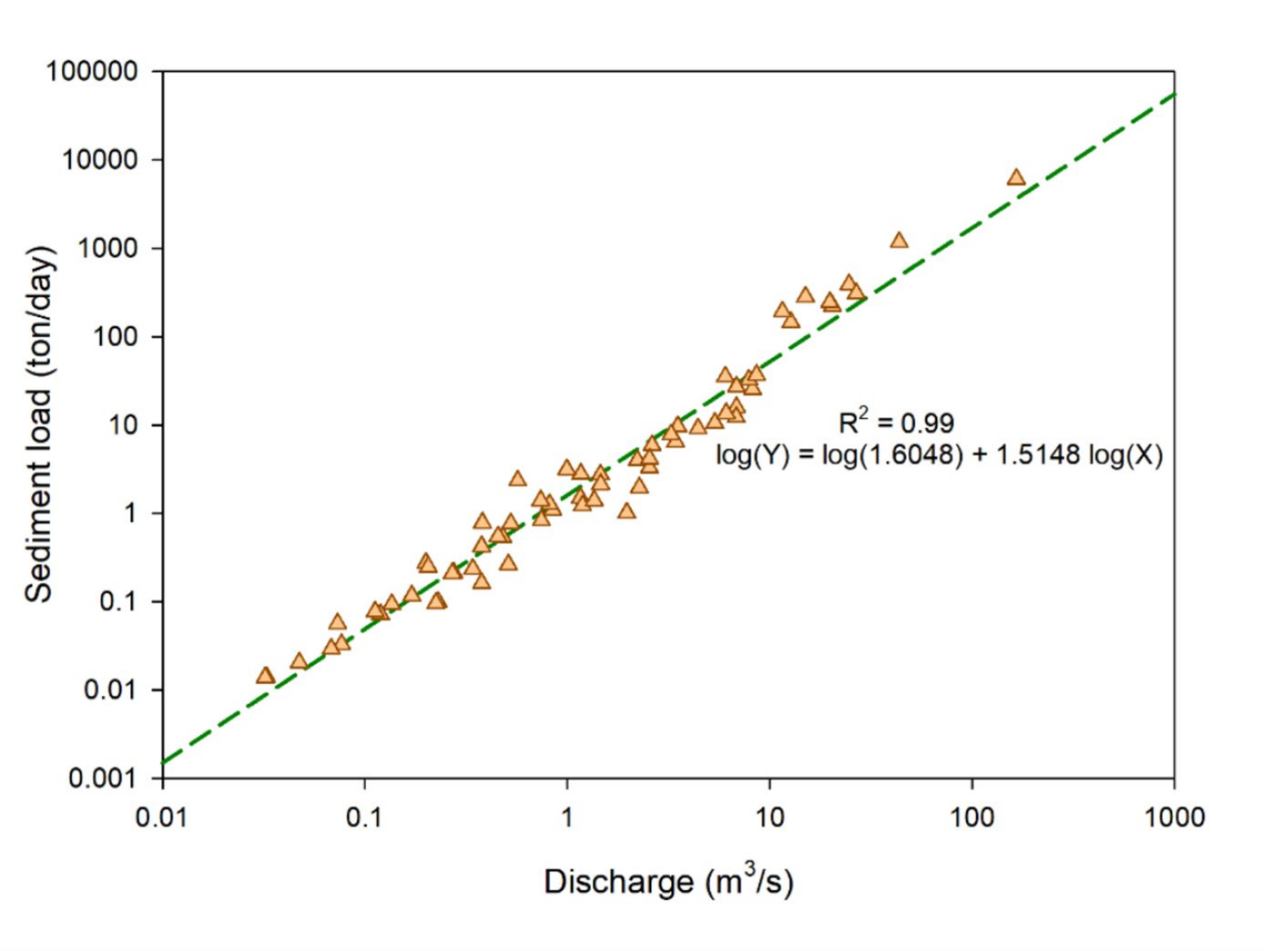
Relationship of observed sediment load versus observed discharge along with the best-fitted regression line and the regression equation

The sediment load time series for the simulation period was created using the fitted SRC, in which *a* = 1.6084 and *b* = 1.5148, with an *R*^2^ value of 0.99 (Fig. 5).

### Model performance

**Fig. 6:**
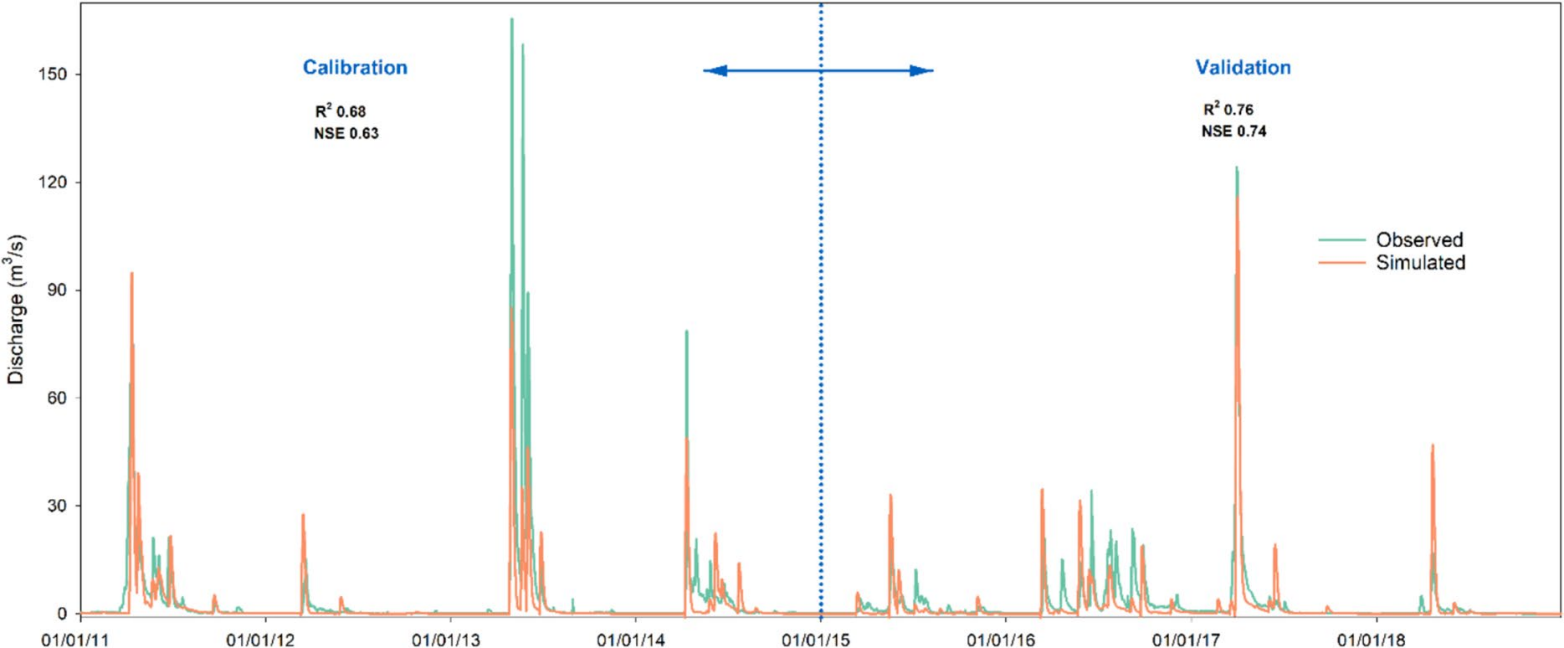
Hydrographs at the watershed outlet for the calibration and validation periods

The DPCA-SWAT model was calibrated against the USGS observed discharge at the outlet of the PR watershed with the parameters listed in Table 2. Nash–Sutcliffe efficiency (NSE) and coefficient of determination (*R*^2^) values were “Satisfactory” to “Good” (Moriasi et al., 2015) for both the calibration and validation periods. Figure 6 shows the simulated and observed discharges at the watershed outlet for the entire simulation period. Overall, the model effectively captured the responses of this depression-dominated watershed under both low- and high-flow conditions. Notably, the model was able to simulate the magnitude of most peak flows, and their distribution, timing, and duration were reasonably characterized and matched those of the observed hydrograph. Some high peak discharges in the years of 2013, 2014, and 2016 were underestimated by the model, likely due to the uncertainties in input data as well as model parameters. Except for those years, the model provided reasonable peak discharges for most of the years. For example, in 2017, the observed peak discharge reached 124.03 m^3^/s, and the simulated peak discharge was 115.80 m^3^/s. The deviation between the observed and simulated peak discharges in 2017 was only 6.6%. Overall, the strong agreement between the DPCA-SWAT simulations and the observed data underscored the capability of the model in the simulation of both low and high flows.

**Table 2:** List of parameters associated with flow, nutrients (TN, TP), and sediment for the DPCA-SWAT model calibration and their calibrated values

Parameter	Description	Calibrated values
r_CN2.mgt	Curve number (for moisture condition II)	− 0.04
r_SOL_K(1).sol	Saturated hydraulic conductivity (mm/hr)	− 0.01
v_SURLAG.hru	Coefficient representing surface runoff lag	11.87
v_GWQMN.gw	Required depth of shallow groundwater to occur return flow (mm)	1229.95
v_ALPHA_BF.gw	Baseflow alpha factor (1/day)	0.60
v_CANMX.hru	Maximum amount of water retained by canopy (mm)	39.60
v_SMTMP.bsn	Base temperature for snowmelt initiation (°C)	4.88
v_TIMP.bsn	Lag factor for snowpack temperature response	0.24
v_CH_K2.rte	Effective hydraulic conductivity of main channel (mm/hr)	66.33
v_CH_N2.rte	Manning’s roughness coefficient for the main channel	0.26
v_NPERCO.bsn	Percolation coefficient for nitrogen	0.19
v_SDNCO.bsn	Nutrient cycling water factor threshold required for denitrification	1.10
r_USLE_K(1).sol	Soil erodibility factor in the USLE equation (K)	− 0.22
v_USLE_P.mgt	Support practice factor in the USLE equation (P)	0.36
v_PRF_BSN.bsn	Factor used to adjust the peak sediment transport rate in the main channel	1.42
v_ADJ_PKR.bsn	Factor used to adjust the peak sediment transport rate in the subbasin (tributary channels)	1.20
v_CH_D50.bsn	Channel bed median particle diameter (µm)	86.46
v_CH_COV2.rte	Channel cover factor	0.75

prefix v_ means the existing parameter value is replaced by a given value; and prefix r_ means the existing parameter value is multiplied by (1 + a given value)

**Fig. 7:**
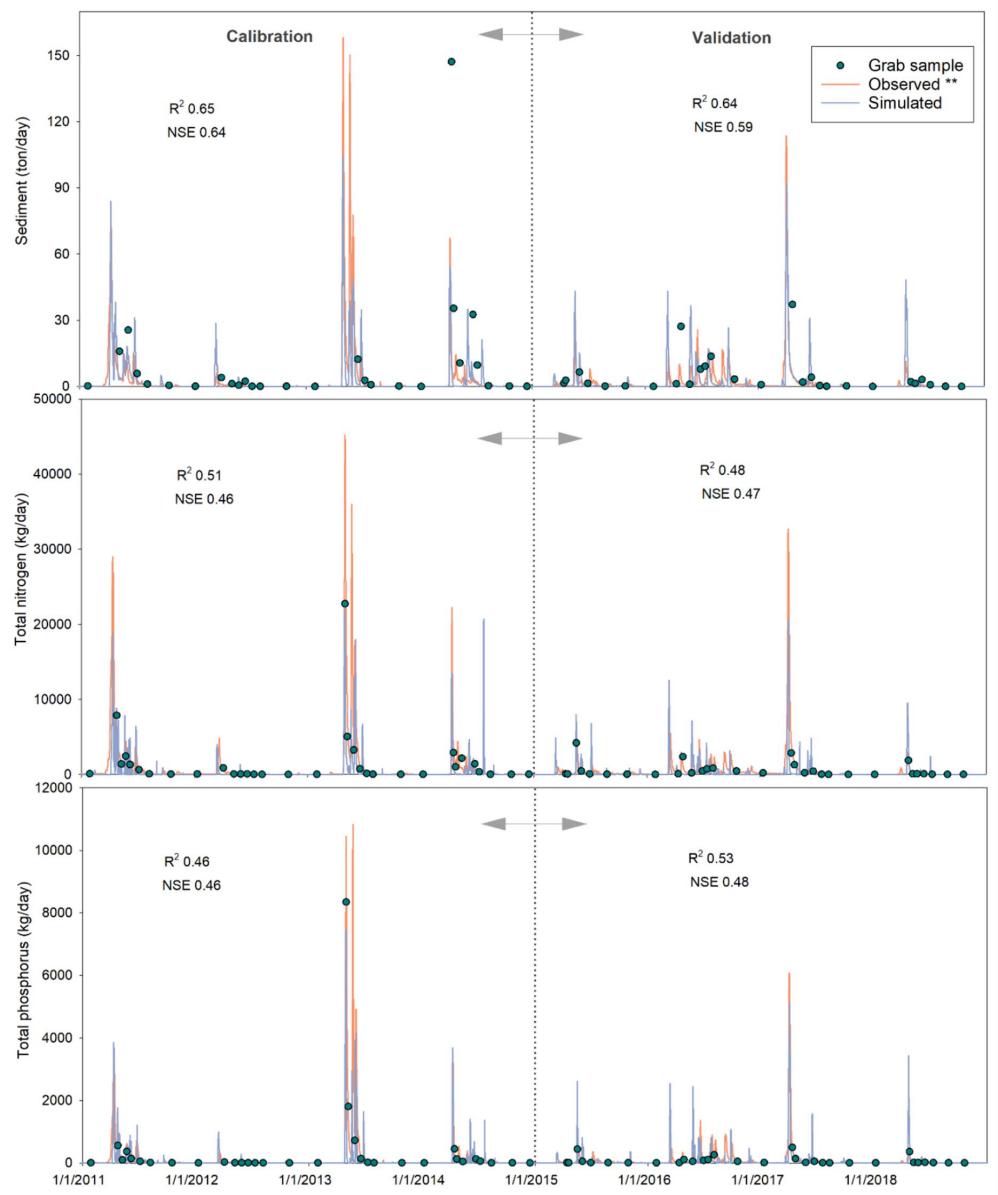
Water quality model performances. Sedigraph and chemographs at the watershed outlet for the calibration and validation periods. Asterisks (**) indicate observed datasets generated by employing the sediment rating curve and the LOADEST program

The model was calibrated against the observed TN, TP, and sediment load time series at the outlet of the PR watershed with the water quality parameters associated with nutrients (TN, TP) and sediments (Table 2). Model performance was satisfactory in simulating daily sediment, TN, and TP loads during both calibration and validation periods (Fig. 7). However, the simulated sedigraph and chemographs missed several extreme peaks in 2013 and some smaller peaks in 2016, likely due to the uncertainties in model parameters, input data, or limited details on the observed data (e.g., timing and magnitude). Overall, the model successfully captured most of the peak loads and lower loads for sediment, TN, and TP. Both NSE and *R*^2^ metrics demonstrated that the water quality model performances ranged from “Satisfactory” to “Good” (Moriasi et al., 2015) for sediment, TN, and TP loads in the calibration and validation periods (Fig. 7).

### Spatiotemporal variability analysis

**Fig. 8:**
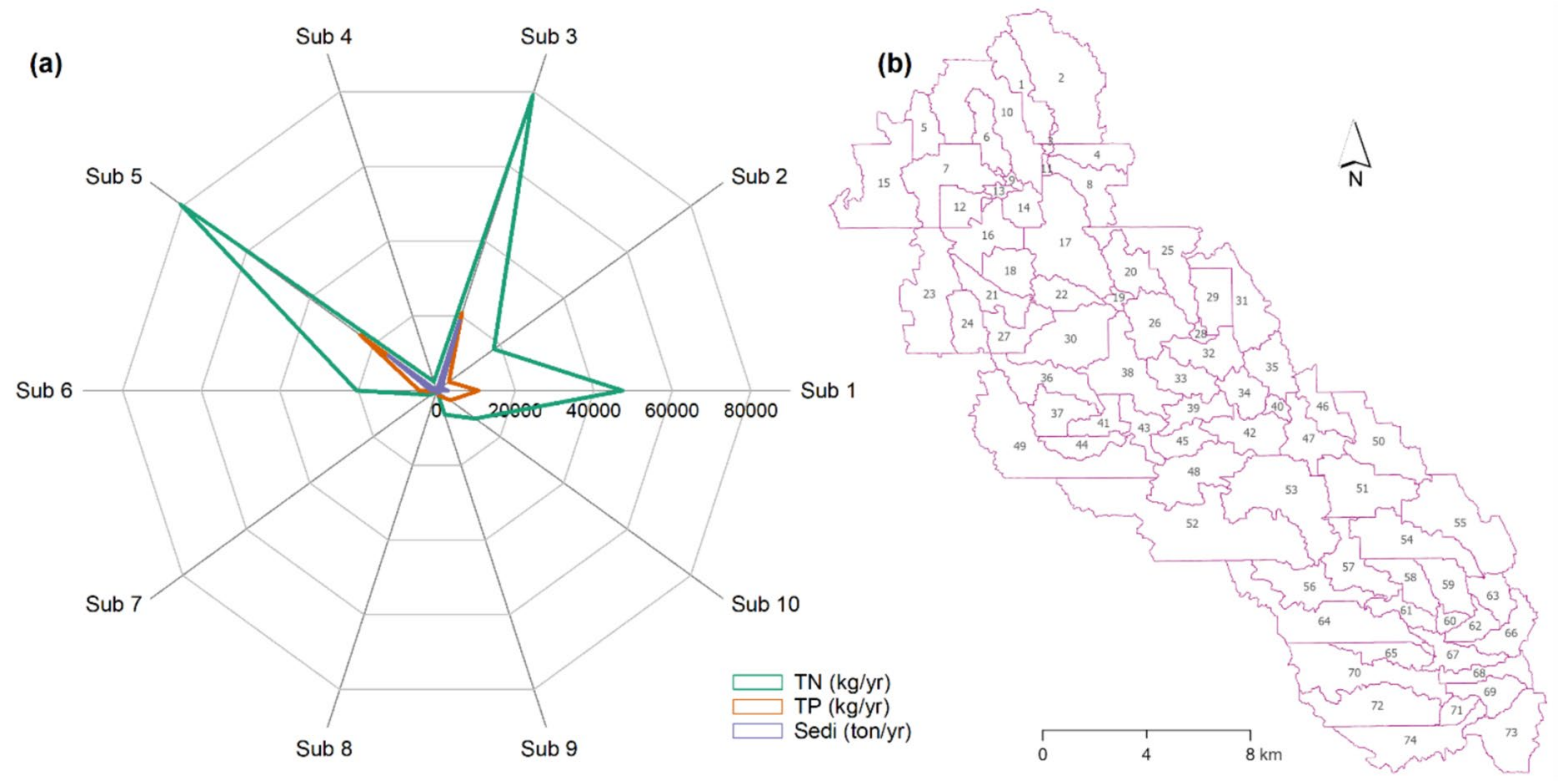
**a** Nutrients and sediment loads in different subbasins in the PR watershed, **b** 74 sub-subbasins of Subbasin 5 with the highest loads of pollutants (TN, total nitrogen; TP, total phosphorus; Sedi, sediment)

For spatial variability analysis, the study focused on selecting the most heavily polluted subbasin. Average annual TN, TP, and sediment loads were plotted in a radar diagram to identify the subbasin with the highest loads. As shown in Fig. 8a, subbasins 1, 3, and 5 were identified as the three heavily polluted subbasins in the watershed. The highest average annual loads were observed in subbasin 5, with 80,640 kg/yr of TN and 24,100 kg/yr of TP. Subbasin 3 showed higher sediment loads with 18,630 ton/yr, followed by subbasin 5 with 15,180 ton/yr. Having the highest loads with all three pollutants, subbasin 5 was hence selected and divided into 74 sub-subbasins for further analysis (Fig. 8b). According to the 2019 Cropland Data Layer (USDA, 2024a), the dominant LULC type in subbasin 5 was agricultural crops (76.32%), followed by pasture (12.85%) and wetlands (3.63%). The total area encompassed 312.98 km^2^, which included 27.07 km^2^ of depressions. Subbasins 4, 7, and 8, located in the downstream part of the PR watershed, are dominated by forests and urban areas (Cropland Data Layer, USDA, 2024a). Due to comparatively lower fertilizer applications in these subbasins, the input and output of TN and TP were significantly lower than those in Subbasin 5, which is dominated by agricultural land use. Our findings agree with Carey et al. (2012), who reported that lower fertilizer applications in urban areas, compared to agricultural lands, reduced the risk of nutrient contamination in waterbodies. In addition, forested land cover enhanced soil stability through extensive and protective roots, making these subbasins less susceptible to erosion. Although Subbasin 9 is not located in the downstream area, the pollution load from this subbasin was lower than that of all other subbasins. The spatial locations of the “source” and “sink” zones in the watershed differ, and their effects on pollutant loads in waterbodies also vary (Shi et al., 2022). Furthermore, the area of Subbasin 9 is the smallest among all the subbasins and, hence, it generated comparatively less pollutant than the others.

**Fig. 9:**
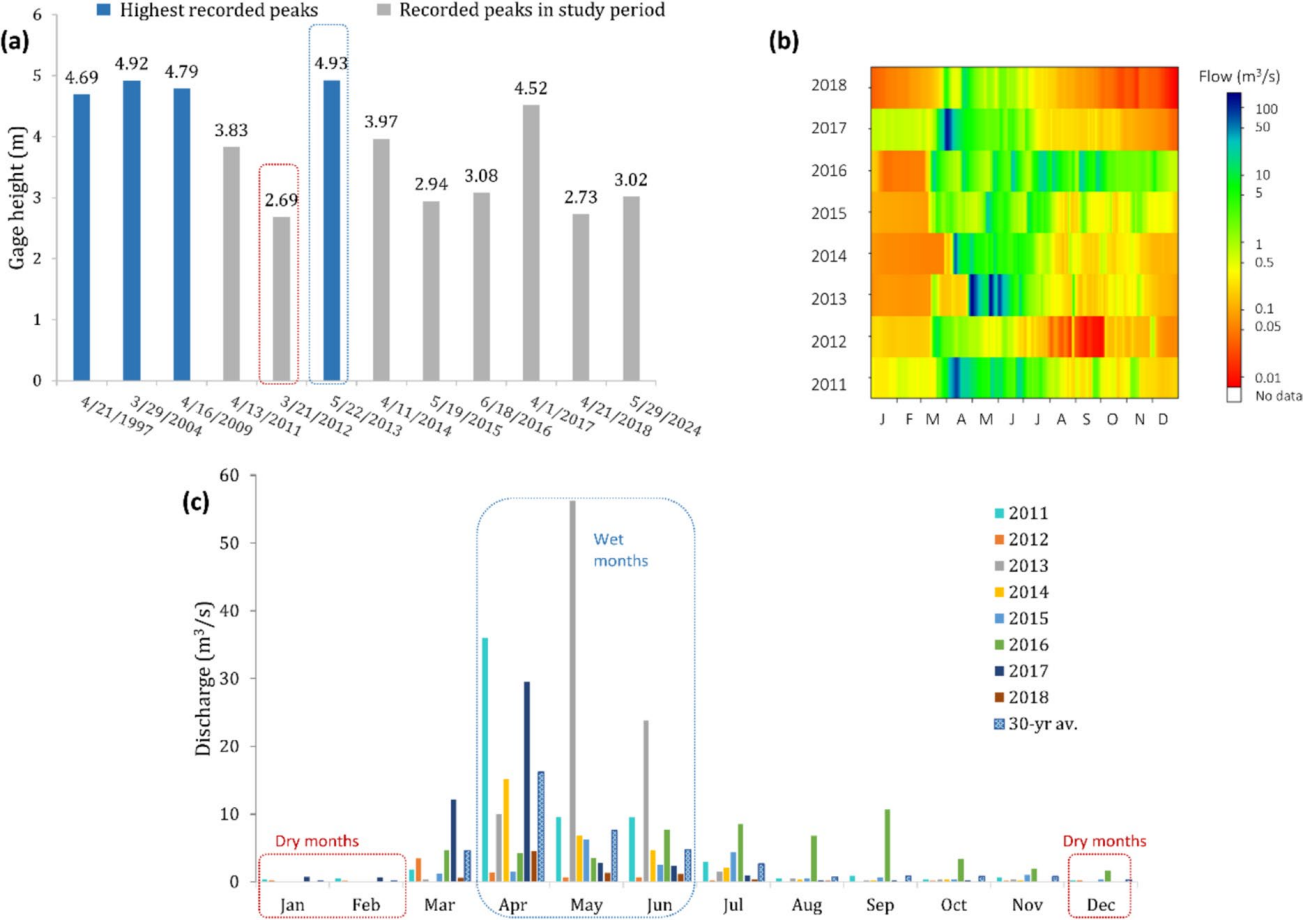
Selection of dry and wet years and months: **a** gage height comparison at the watershed outlet; **b** raster hydrograph of daily flow (*x*-axis: months, *y*-axis: years) (figure adopted and modified from the USGS WaterWatch, 2024); **c** selection of dry and wet months based on the 30-year average monthly discharge at the watershed outlet

**Fig. 10:**
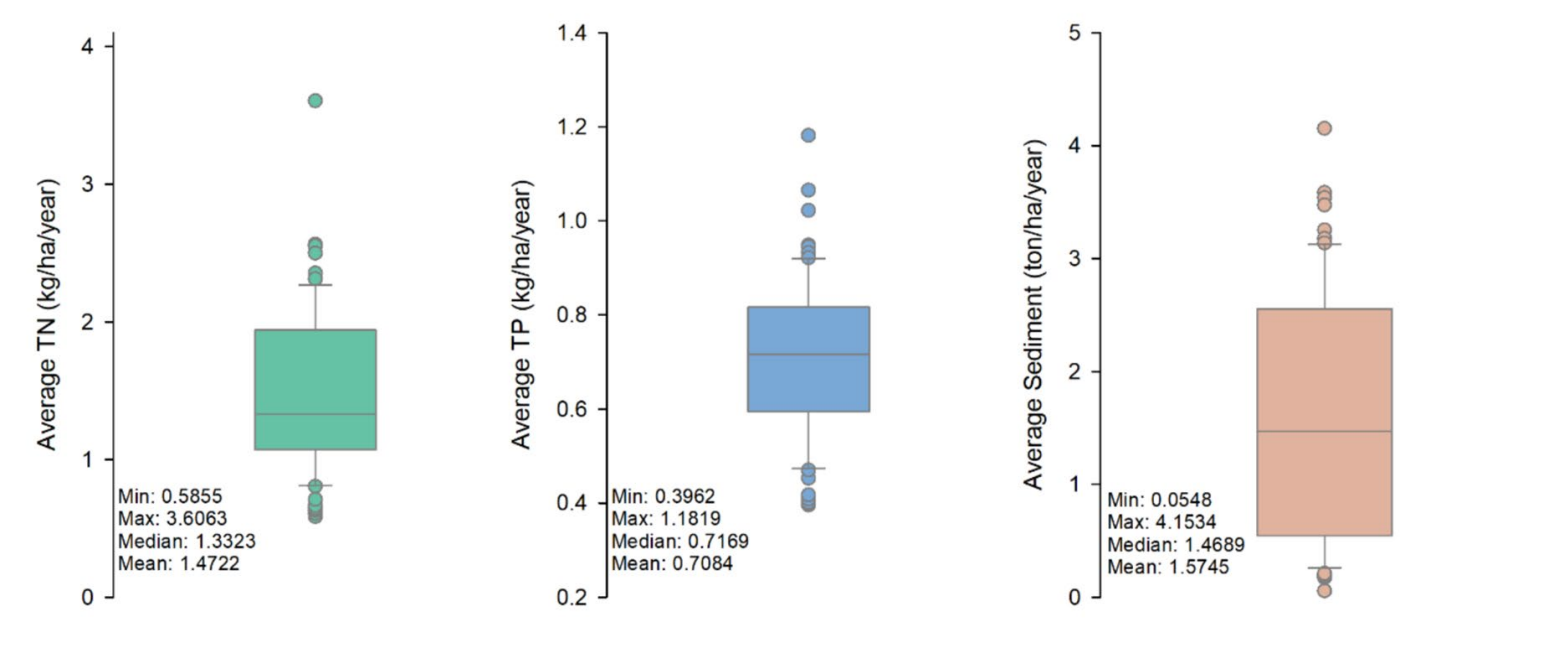
Distributions of annual TN, TP, and sediment loads for the selected subbasin

For selecting different temporal settings, the driest and wettest years were identified by comparing the average annual discharge with a 30-year average discharge in 1994–2023 as well as with the annual gage height at the watershed outlet. The average annual discharge in 2013 was 7.82 m^3^/s, which was 2.38 times higher than the 30-year average of 3.29 m^3^/s (Table 3). In 2012, however, the average annual discharge was only 0.67 m^3^/s, which was 4.91 times lower than the 30-year average. The USGS gage measurements at the watershed outlet indicated that the watershed experienced significant floods in 1997, 2004, 2009, and 2013, with the highest annual water levels of 4.69 m in 1997, 4.92 m in 2004, 4.79 m in 2009, and 4.93 m in 2013 (Fig. 9a). In other years, the watershed experienced normal annual flows. Consistent with the lowest average discharge, the lowest gage height (2.69 m) was observed in 2012 (Table 3 and Fig. 9a). The raster hydrograph at the watershed outlet shows that the daily streamflow never exceeded 15 m^3^/s in 2012, whereas it reached 165 m^3^/s in 2013 (Fig. 9b). The median discharge in 2012 was 0.17 m^3^/s and in 2013 was 0.24 m^3^/s. Hence, 2012 and 2013 were selected as the driest year and the wettest year for spatiotemporal variability analysis. Following the same approach, the dry and wet months were selected in this study. April, May, and June were found to be the wettest months, while January, February, and December were found to be the driest months (Fig. 9c). Hence, these months of the entire simulation period were selected to examine the temporal variations between dry and wet months. For the selected subbasin, boxplots of TN, TP, and sediment loads are shown in Fig. 10, along with summary statistics. The median value of average annual TN load was 1.33 kg/ha/year, TP load was 0.72 kg/ha/year, and sediment load was 1.47 ton/ha/year. The standard deviation of sediment was higher than those of TN and TP.

**Table 3:** Average annual discharge and the highest annual gage height at the watershed outlet for the entire simulation period, along with the 30-year (1994–2023) average annual discharge

	2011	2012	2013	2014	2015	2016	2017	2018	30-yr av
Annual average discharge (m^3^/s)	5.67	0.67	7.82	2.52	1.52	3.99	4.72	0.71	3.29
Highest annual gage height (m)	3.83	2.69	4.93	3.97	2.94	3.08	4.52	2.73	-

#### Autocorrelation analysis with Moran’s index

**Fig. 11:**
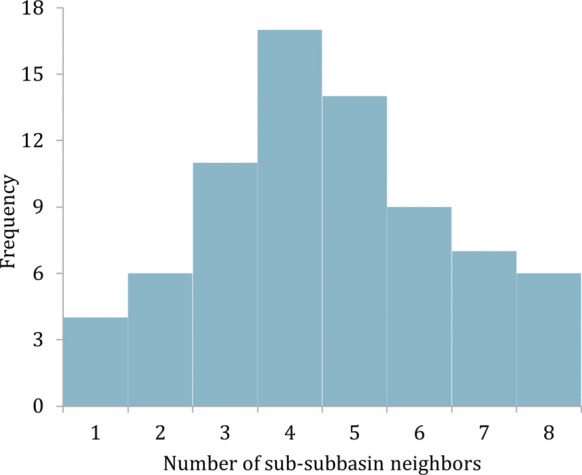
Histogram of neighbor relationships among sub-subbasins for calculating the weight matrix (*W*) using the inverse distance weighting method based on the proximity and influence of adjacent sub-subbasins

Calculating the spatial weight matrix (W) for autocorrelation analysis is both a prerequisite and a critical step. Following Shen et al. (2022), *W* was calculated using the inverse distance weighting method, which involved applying the inverse operation to the Euclidean distance between two locations for any two variables. The minimum, maximum, and median number of sub-subbasin neighbors were 1, 8, and 4, reflecting a bell-curved Gaussian distribution (Fig. 11).

**Fig. 12:**
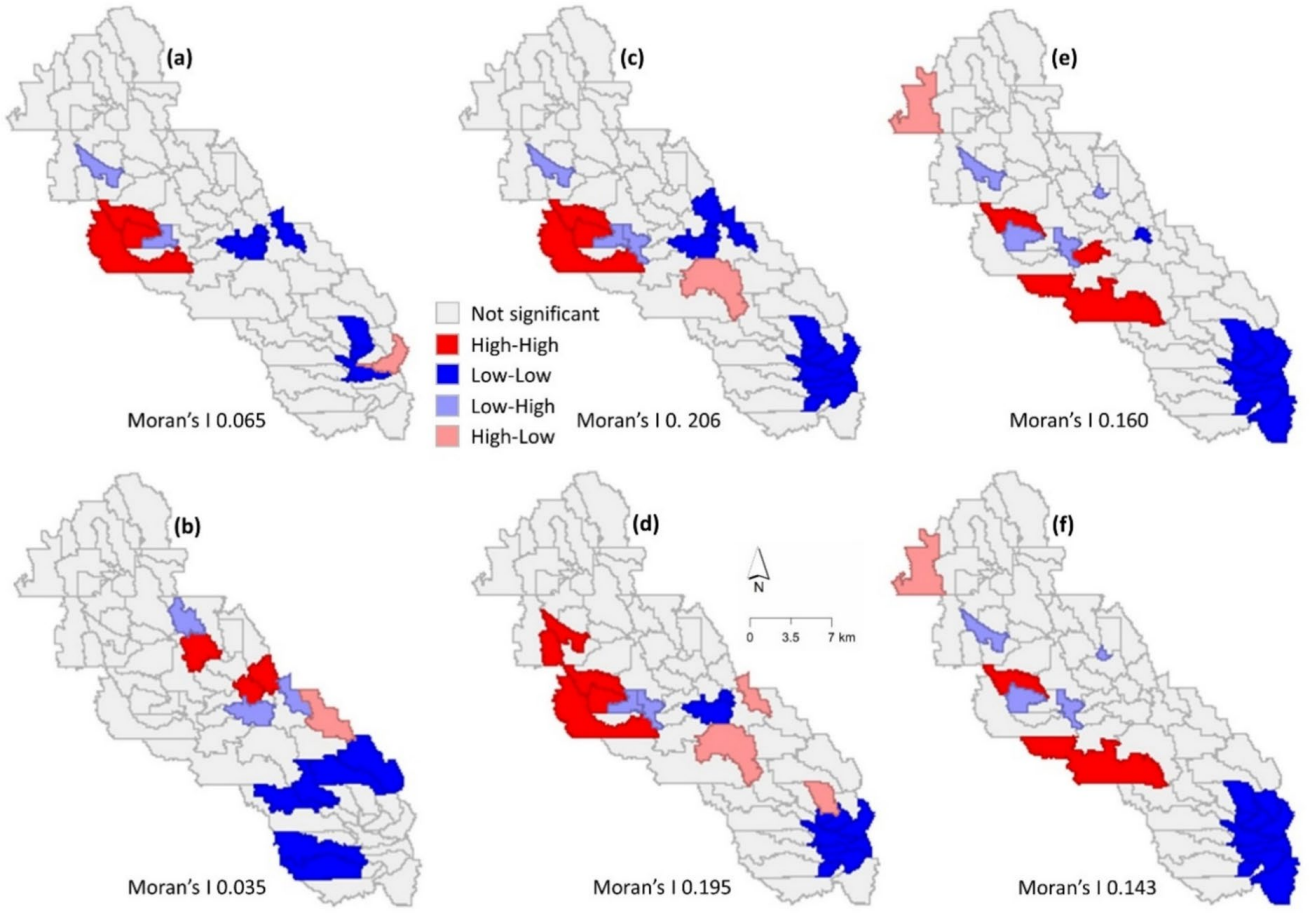
Spatial autocorrelation analysis for **a** TN in the dry year; **b** TN in the wet year; **c** TP in the dry year; **d** TP in the wet year; **e** sediment in the dry year; **f** sediment in the wet year

Spatial autocorrelation analysis indicated statistically significant spatial clustering of the pollutants (TN, TP, and sediment) in both dry and wet years. From dry to wet year, Moran’s *I* value decreased from 0.065 to 0.035 for TN, 0.206 to 0.195 for TP, and 0.160 to 0.143 for sediment, indicating a stronger correlation in the dry year than the wet year (Fig. 12). In the dry year, high-high clustering was observed in the central part of the area for TN and TP. However, high-high clustering for TN was observed at different locations in the wet year. Similar sediment hotspot locations were observed in both dry and wet years. For all pollutants, low-low clustering was observed in the downstream area, which can be attributed to the land use type. Note that the upstream area was mainly agricultural lands, while the downstream area was dominated by forest and urban LULC (Cropland Data Layer, USDA, 2024a). The downstream area benefitted from the strong root systems of forests, making it less prone to sediment erosion. Lower fertilizer application in urban areas, compared to agricultural lands, further reduced the risk of nutrient contamination to waterbodies (Carey et al., 2012).

Using the 90-year (1934–2023) peak discharges at the PR watershed outlet, flood frequency analysis was performed for better interpretation of the spatial hotspots. Table 4 presents the analysis results and summarizes the statistics of the 2012 and 2013 discharge data. The median discharge in 2013 was nearly twice that of 2012. The maximum discharges were 15.21 m3/s in 2012 and 165.37 m3/s in 2013, corresponding to return periods of 1.38 years in 2012 and 22.5 years in 2013. For the wet year, Moran’s *I* spatial autocorrelation results showed a weaker correlation.

**Table 4:** Flood frequency analysis based on the 90-year (1934–2023) peak discharges at the PR watershed outlet and summary of statistics for the 2012 and 2013 daily discharge data

Peak discharge (m^3^/s)	Return period (yr)	Peak discharge (m^3^/s)	Return period (yr)	Statistics	2012	2013
0.48	1	144.98	15	Median (m^3^/s)	0.17	0.24
*15.21*	*1.38*	*165.37*	*22.5*	Min. (m^3^/s)	0.01	0.07
38.51	2	235.30	30	Max. (m^3^/s)	15.21	165.37
86.37	5	297.33	45	Std. deviation (m^3^/s)	1.51	23.97
137.62	10	331.31	90			

**Fig. 13:**
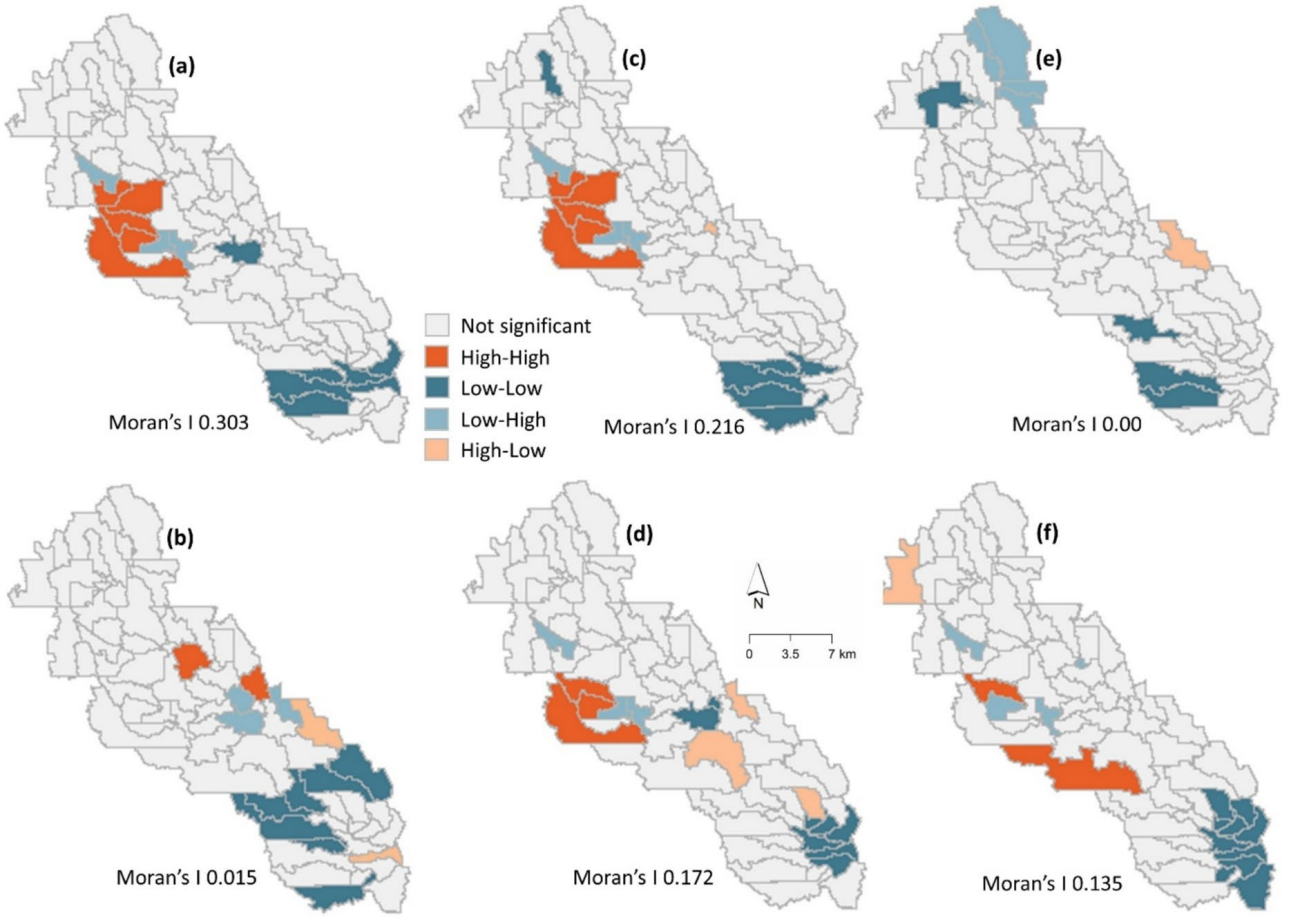
Spatial autocorrelation analysis of **a** TN during dry months; **b** TN during wet months; **c** TP during dry months; **d** TP during wet months; **e** sediment during dry months; **f** sediment during wet months

Similar to the yearly autocorrelation results, the monthly TN and TP results showed lower Moran’s *I* values for dry months. Moran’s *I* statistics decreased from 0.303 to 0.015 for TN and from 0.216 to 0.172 for TP from dry to wet months, indicating a stronger correlation in dry months than wet months (Fig. 13). The higher Moran’s *I* value for TN (0.303) suggests a less than 5% likelihood that the observed clustering could be random. There was mild clustering of sediment during the dry months. This does not imply that there was no sediment pollution, while the statistical test did not detect any statistically significant clustering. Instead, sediment loads were scattered throughout the subbasin.

#### Geodetector analysis for identifying key factors

Geodetector revealed four key influencing factors for nutrients and sediment pollution (Table 5). Precipitation was a significant single indicator for TN and sediment pollution during the dry year (Table 5). Lower *q*-values of precipitation indicated lower influencing power. Higher *q*-values of USLE_C and LAI suggested that they had greater influencing power than precipitation and LULC. Despite the high *q*-values, LAI was not a statistically significant single indicator in any temporal setting for any variable. In both dry and wet years, LULC emerged as a statistically significant single indicator, followed by precipitation and USLE_C. Among these, USLE_C showed the highest *q*-value (97.94%) for TN during the wet year (Table 5). LULC was the only indicator that showed statistically significant *q*-values for TN during the wet year, TP during the dry year, and sediment during both dry and wet years. Precipitation showed statistically significant *q*-values of 16.12% for TN and 17.10% for TP during the dry year (Table 5).

**Table 5:** Geodetector *q*-values for evaluating the impacts of a single factor on different variables in dry and wet years

Variables in dry and wet years		Single indicator			
		Precipitation	LULC	USLE_C	LAI
TN	Dry year	5.70%	19.70%	90.95%	86.29%
	Wet year	5.01%	*24.67%	*97.94%	62.92%
TP	Dry year	*16.12%	*30.51%	89.73%	87.34%
	Wet year	9.53%	30.02%	87.49%	90.74%
Sedi	Dry year	*17.10%	*29.73%	87.42%	83.12%
	Wet year	9.47%	*24.81%	84.21%	85.52%

*TN* total nitrogen, *TP* total phosphorus, *Sedi* sediment, *LULC* land-use land-cover, *USLE_C* land cover and management factor for the universal soil loss equation, *LAI* leaf area index

*Statistically significant tests for *p* < 0.05

Table 6 shows statistically significant *q*-values for evaluating the influences of interactions between independent variables and factors in dry and wet years. Although precipitation, as a single indicator, has a lower influence than others, its interactions with the indicators (LULC, USLE_C, and LAI) resulted in higher influences. Specifically, precipitation interacting with LULC showed statistically significant influences on TN during both dry and wet years, as well as on TP and sediment during the wet year. Precipitation showed higher influences on sediment (97.69%) for USLE_C and on TN (83.03%) for LAI in the wet year (Table 6). The highest influence of 99.34% on TN pollution was observed for the interactions between LULC and LAI during the wet year. Overall, precipitation and LULC were key factors for all three pollutants: TN, TP, and sediment. The interactions between precipitation and LULC, precipitation and LAI, and LULC and LAI were key factors for TN pollution. However, the interaction between precipitation and ULSE_C was identified as the key factor responsible for sediment pollution during the wet year.

**Table 6:** Statistically significant Geodetector *q*-values for evaluating the influences of interactions between independent variables and factors in dry and wet years

Variables in dry and wet years		Interacting factors			
		PCP ∩ LULC	PCP ∩ USLE_C	PCP ∩ LAI	LULC ∩ LAI
TN	Dry year	*35.76%	-	-	-
	Wet year	*57.49%	-	*83.03%	*99.34%
TP	Dry year	-	-	-	-
	Wet year	*43.08%	-	-	-
Sedi	Dry year	-	-	-	-
	Wet year	*40.01%	*97.69%	-	-

*TN* total nitrogen, *TP* total phosphorus, *Sedi* sediment, *PCP* precipitation, *LULC* land-use land-cover, *USLE_C* land cover and management factor for the universal soil loss equation, *LAI* leaf area index

*Statistically significant tests for *p* < 0.05

USLE_C denotes the land cover and management factor in universal soil loss equation, which reflects how different conservation practices influence soil loss and how the soil loss potential varies over time during crop rotations, construction activities, or other management approaches (USDA-NRCS, 2024). Geodetector indicated that the interaction between precipitation and USLE_C was observed as a key factor responsible for sediment pollution during the wet year. Besides, nitrogen and phosphorus fertilizer application rates vary across LULC types (Franzen, 2010), making LULC a key influencing factor for TN and TP pollution. Additionally, LAI was a key influencing factor specifically for TN pollution during the wet year. Geodetector analysis suggests that an indicator may not act as a key factor alone; however, when it interacts with another factor, it can have higher influences on nutrients and sediment pollution.

## Discussion

Incorporating the filling-spilling processes of surface depressions into modeling is critical for depression-dominated watersheds. A recently developed DPCA-SWAT model (Khanaum et al., 2025) was applied to a depression-dominated watershed to identify pollutant hotspots and their influencing factors using autocorrelation and Geodetector analyses. The DPCA-SWAT model separately simulates DAs and NDAs and calculates the threshold-controlled overflow from DAs at each time step, thereby redistributing this outflow over the associated NDAs as additional excess surface runoff. Note that there are some limitations and uncertainties of the study. For example, to simulate water release from a depression, the model employs a simple mass balance approach, assuming that the depression does not release any water until it is fully filled (Khanaum et al., 2025). Furthermore, to calculate ponding and contributing areas, it is assumed that depressions are cone-shaped (Khanaum et al., 2025). Additionally, due to the complex topography of the study area, the meteorological data used in the study may introduce some uncertainty, as they might not fully capture the local variations.

The DPCA-SWAT uses a simplified approach for nutrients and sediment transport, assuming that the system is completely mixed. That is, once nutrients and sediments enter a depression, they are instantaneously distributed throughout the depression storage. The fate and transport of nutrients, including transformation between nutrient pools, as well as other processes such as fertilizer application, fixation, atmospheric deposition, plant uptake, leaching, ammonia volatilization, nitrification, denitrification, mineralization, erosion, detachment, and deposition, were simulated by SWAT for non-depressional areas.

Due to pre-filling the DEM for all depressions during the watershed delineation stage, the conventional SWAT model ignored the actual ponding storage. As a result, it overpredicted the average peak flows at the watershed outlet by 15.74% in the dry year and 22.00% in the wet year. Accounting for depressional storage, the average peak flows simulated by the DPCA-SWAT model were 11.16 m^3^/s in 2012 (dry year) and 55.63 m^3^/s in 2013 (wet year). The reason was that, with lower precipitation intensity, larger depressions may be partially filled during most of the time in the dry year and even did not contribute surface runoff to the outlet. However, by ignoring the filling-spilling dynamics of depressions, the conventional SWAT model simulated surface runoff for the entire watershed and consequently overestimated peak flows compared to the DPCA-SWAT model. Similar overestimations were observed for nutrients and sediment loads. Due to ignoring dynamic contributing area and ponding storage, the conventional SWAT model failed to simulate the dynamics changes in discharge, TN, TP, and sediment retained in depressions. In contrast, the DPCA-SWAT model accounted for actual ponding storage and better captured the threshold-controlled dynamic filling-spilling processes of surface depressions.

A detailed study on the effects of surface depressions on discharge at both watershed and individual depression scales, along with a comparison of the traditional SWAT model, the Fixed Partial Contributing Area (FPCA) model, and the DPCA-SWAT model, was conducted by Khanaum et al. (2025). That study quantified the spatiotemporal variations in ponding and contributing areas, demonstrating that discharge was strongly influenced by complex terrain, and highlighted the potential issues associated with traditional modeling based on depressionless DEMs. Khanaum et al. (2025) also demonstrated that smaller depressions released water more quickly, while larger depressions either contributed nothing or released substantial volumes of water depending on the storage capacity, precipitation duration and intensity, and other factors. Given the scope of the present study, the focus was not on the effects of surface depressions on discharge or pollutant loads. Instead, the objective was to identify pollutant hotspots and their key driving factors by employing the DPCA-SWAT model, which explicitly accounted for the fill-spill dynamics of depressions. Due to the lack of available observed data, the model was calibrated using the flow and water quality data at the watershed outlet. The reliability of the hydrologic and water quality simulations would likely be improved if additional monitoring data within the watershed are available.

This study focused on applying the DPCA-SWAT model for water quality assessment, detecting pollutant hotspots using Moran’s *I* autocorrelation, and identifying the key influencing factors. We limited the Geodetector analysis to four factors: precipitation, LULC, USLE_C, and LAI. Among these four variables, LAI and USLE_C are, to some extent, interrelated with LULC, but there are unique dissimilarities as well. USLE_C represents the effect of canopy cover, prior land use, cropping rotation, and management practices on soil erosion estimation, while LAI refers to the leaf area per unit ground area and serves as an indicator of vegetation health and density. LULC categorizes lands based on their use (e.g., urban, agricultural, wetlands, water bodies). Typically, LULC changes over longer timescales, such as decades, whereas both USLE_C and LAI vary on a yearly or seasonal basis. Yet we cannot ignore the possibility that the high *q*-values of USLE_C and LAI may indicate that they have greater influencing power than LULC and precipitation. However, this influence was balanced by the *F*-test results. Greater statistical significance was observed for precipitation and LULC compared to USLE_C and LAI. Since LAI and LULC are interrelated, their interaction may somewhat reduce the significance of each as an independent influencing factor. However, incorporating both variables allows for a more detailed understanding of vegetation health and density across different land-use types, thereby improving the strength of our analysis.

In a dry year, precipitation and LULC emerged as statistically significant single indicators for TP and sediment, while in a wet year, LULC was a statistically significant single indicator for TN and sediment, and USLE_C for TN. The areas with higher TN and sediment loads were mainly concentrated in the middle part of the studied subbasin, which was the primary area of agricultural land and farmland, with contributions from upstream agricultural runoff leading to higher pollution in the wet year. Different types of LULC can alter the hydrological cycle through interception, infiltration, and evapotranspiration, thereby influencing the transport of terrestrial nutrients (Li et al., 2021). During the wet year, we observed that the interaction of precipitation with LULC significantly influenced TN, TP, and sediment pollution. This is largely because land use regulated water flow and the exchange of nutrients and energy between different land use types, a phenomenon that becomes especially pronounced in wet years (Ricart et al., 2015; Shi et al., 2022). The interactions between precipitation and LAI, and between LULC and LAI, were responsible for TN pollution in the wet year, while the interaction between precipitation and USLE_C was identified as the key factor responsible for sediment pollution during the wet year. LAI and LULC, specifically, the complexity of vegetation density, have a distinct impact on TN pollution (Shi et al., 2022). The combination of precipitation and USLE_C in the wet year, mainly high flow together with canopy cover, cropping, and management practices, affects the erosion process and thus contributes to the sediment load.

Although both autocorrelation and Geodetector analyses provide valuable insights, uncertainties associated with these methods may introduce certain limitations. Autocorrelation analysis is associated with zoning (e.g., grid size, administrative/subbasin boundaries) uncertainty and weight matrix uncertainty. It is highly sensitive to how spatial relationships are defined (e.g., distance, adjacency), which can substantially influence the results (Anselin, 2024). Therefore, careful consideration is required when selecting the calculation method for spatial weight matrix (*W*). Similarly, the Geodetector method identifies statistical associations but does not establish true causality (Wang et al., 2010). Moreover, it may fail to detect certain risks; for example, some types of environmental pollution may not exhibit distinct spatial patterns, or the study area may be too small to reveal such patterns. Despite these limitations, both methods remain useful for identifying pollutant hotspots and influencing factors, providing valuable evidence to support decision-making for researchers and policymakers. The modeling approach presented in this study, combined with hotspot and influencing factor identification, can be applied to watersheds with similar topographic and climatic conditions, as well as to other depression-dominated watersheds in different climate settings, to improve hydrologic and water-quality modeling.

## Summary and conclusions

A recently proposed DPCA-SWAT model (Khanaum et al., 2025) was applied to the Park River watershed in North Dakota, U.S. To facilitate model calibration with limited observed water quality data, a sediment rating curve was developed and the LOADEST program was applied. Using the USGS observed discharge, TN, TP, and sediment loading data, observed time series were generated for TN, TP, and sediment loads. The model performances in the simulations of discharge and water quality were satisfactory. One subbasin with the highest loads of pollutants was selected for spatial variability analysis for TN, TP, and sediment pollution. The driest and wettest years were selected by comparing them with the 30-year discharge data. The dry months (January, February, and December) and wet months (April, May, and June) were further selected based on the monthly discharges over the entire simulation period. The DPCA-SWAT model results were used to perform a spatial autocorrelation test to detect pollutant hotspots during those different temporal settings. In addition, a flood frequency analysis with 90-year peak discharges at the PR watershed outlet was performed to better explain the spatial hotspot analysis results. Geodetector test was also utilized to identify the key influencing factors for the TN, TP, and sediment pollution.

The spatial autocorrelation results with Moran’s *I* indicated a stronger correlation for all three pollutants (i.e., TN, TP, and sediment) during the dry year and dry months than the wet year and wet months. The hotspots were also more pronounced during the periods of lower flood frequency. Moreover, it was found that LULC influenced pollution levels. The downstream areas dominated by forests and urban areas benefited from robust root systems and reduced fertilizer applications, making them less susceptible to TN, TP, and sediment pollution.

Geodetector test revealed the dominant factors that were responsible for the TN, TP, and sediment pollution in the study area at different spatiotemporal settings. Both single factor and multiple factor interaction analyses were performed to characterize the spatial variability of the related pollution. The Geodetector test results demonstrated that the interaction of precipitation and LULC was the main indicator influencing TN, TP, and sediment pollution during the wet year. The interaction of precipitation and USLE_C emerged as the most significant influencing factor for sediment pollution during the wet year. The results suggested that an indicator may not be a key factor on its own; however, when combined with other factors, it can have a more significant influence. This study highlights the effectiveness of the methods developed for identifying pollutant hotspots, their spatiotemporal variations, and key driving factors, offering valuable guidance for implementing best management practices to control nutrients and sediment pollution in a watershed.

## Supplementary Information

Below is the link to the electronic supplementary material.

ESM1 (PDF 1.28 MB**)**

## Data Availability

The data presented in this paper can be provided upon request to the corresponding author.
