# Supplementary material for: Spatiotemporal variability analysis of nutrient and sediment loads in surface water using improved hydrologic modeling for depression-dominated watersheds
Source: Environ Monit Assess. 2026 Mar 5;198(3):281. doi: 10.1007/s10661-026-15119-1 (PMC12963125; doi:10.1007/s10661-026-15119-1)
Supplement: Supplementary file 1 — (PDF 1.28 MB) [file 10661_2026_15119_MOESM1_ESM.pdf]

Spatiotemporal Variability Analysis of Nutrient and Sediment Loads in Surface Water Using Improved Hydrologic Modeling for Depression-Dominated Watersheds

Mosammat Mustari Khanaum

Department of Civil, Construction and Environmental Engineering (Dept 2470), North Dakota State University, PO Box 6050, Fargo, ND 58108-6050, USA; Email: mosammat.khanaum@ndsu.edu (Corresponding author)

Marinus L. Otte

Wet Ecosystem Research Group, Department of Biological Sciences (Dept 2715), North Dakota State University, PO Box 6050, Fargo, ND 58108-6050, USA; Email: marinus.otte@ndsu.edu

Xuefeng Chu

Department of Civil, Construction and Environmental Engineering (Dept 2470), North Dakota State University, PO Box 6050, Fargo, ND 58108-6050, USA; Email: xuefeng.chu@ndsu.edu (Corresponding author)

Supplemental Information

Theoretical Explanation of the DPCA-SWAT Model

The DPCA-SWAT model (Khanaum et al., 2025) incorporates the filling-spilling processes of surface depressions. The modeling approach involves four main steps: delineating the watershed and defining subbasin boundaries; extracting topographic parameters required for the DPCA algorithm; performing the DPCA modeling for depressional areas (DAs); and finally, conducting the DPCA-SWAT modeling based on the DPCA output (Khanaum et al., 2025). In the DPCA modeling framework, each subbasin is divided into DA and non-depressional area (NDA), which are simulated separately. In the SWAT modeling, the area corresponding to DA is excluded so that it only simulates surface runoff for the NDA. The DPCA component captures temporal changes in surface depressions, estimating contributing areas, storage capacity, and water release from depressions at each time step. The water released from all depressions within a subbasin is aggregated and added to the SWAT model by evenly distributing the released water as additional equivalent rainfall excess over the subbasin. Afterward, the channel routing process is performed by SWAT.

To determine water release from surface depressions, dynamic changes in water balance are simulated for each individual depression, assuming that a depression does not release water until it is completely filled. The ponded water released from depressions is then incorporated into the SWAT modeling for the corresponding subbasins. The DPCA model calculates water released from a depression under two conditions: (1) if the ponded water volume of a depression is less than or equal to its storage capacity, no water is released; (2) if the ponded water volume of a depression is greater than its storage capacity, the model calculates the water release as the difference between the ponded water volume at a given time step and the storage capacity of the

depression (Khanaum et al., 2025). The outflow from a depression can be expressed as (Khanaum et al., 2025):

$$DO_{i,j,k} = \begin{cases} 0 & for\ \ S_{i,j,k} \leq S_{i,j}^{mx} \\ \left(S_{i,j,k} – S_{i,j}^{mx}\right) & for\ \ S_{i,j,k} > S_{i,j}^{mx} \end{cases} \tag{1}$$

in which,

$$S_{i,j,k} = S_{i,j,k-1} + \left(PA_{i,j,k-1} \times P_{i,j,k}\right) + \left(CA_{i,j,k-1} \times R_{i,j,k}\right) - EV_{i,j,k} - PR_{i,j,k} \tag{2}$$

where $DO_{i,j,k}$ is the outflow from surface depression *j* in subbasin *i* during time step *k* [L^3]; $S_{i,j,k}$ and $S_{i,j,k-1}$ are the storage volume of surface depression *j* in subbasin *i* at time step *k* and *k-1* [L^3]; $S_{i,j}^{mx}$ is the maximum storage capacity of surface depression *j* in subbasin *i* [L^3]; $PA_{i,j,k-1}$ is the ponding area of depression *j* in surface subbasin *i* at time step *k-1* [L^2]; $PCP_{i,j,k}$ is the precipitation over surface depression *j* in subbasin *i* during time step *k* [L]; $CA_{i,j,k-1}$ is the contributing area of surface depression *j* in subbasin *i* at time step *k-1* [L^2]; $R_{i,j,k}$ is the surface runoff generated from the contributing area of surface depression *j* in subbasin *i* during time step *k* [L]; $EV_{i,j,k}$ is the volume of water loss through evaporation from depression *j* in subbasin *i* during time step *k* [L^3]; and $PR_{i,j,k}$ is the percolation loss of depression *j* in subbasin *i* during time step *k* [L^3] (Khanaum et al., 2025).

The model updates ponding storage ($S_{i,j,k+1}$) and ponding area ($PA_{i,j,k+1}$) by assuming that depressions are cone-shaped, and contributing area ($CA_{i,j,k+1}$) for time step *k+1* by the following equations (Khanaum et al, 2025):

$$S_{i,j,k+1} = \begin{cases} S_{i,j,k} & for\ \ S_{i,j,k} < S_{i,j}^{mx} \\ S_{i,j}^{mx} & for\ \ S_{i,j,k} \geq S_{i,j}^{mx} \end{cases} \tag{3}$$

$$PA_{i,j,k+1} = PA_{i,j}^{mx} \left(\frac{PS_{i,j,k+1}}{PS_{i,j}^{mx}}\right)^{\frac{2}{3}} \tag{4}$$

$$CA_{i,j,k+1} = AP_{i,j} - PA_{i,j,k+1} \tag{5}$$

Finally, the DPCA model initiates a subbasin loop to calculate the aggregated ponding area, contributing area, and ponding storage at time step *k+1*, and the total ponded water released from a subbasin during time step k, using the following equations (Khanaum et al, 2025):

$$PA_{i,k+1}^{B} = \sum_{j=1}^{Dep_i} PA_{i,j,k+1} \tag{6}$$

$$CA_{i,k+1}^{B} = \sum_{j=1}^{Dep_i} CA_{i,j,k+1} \tag{7}$$

$$S_{i,k+1}^{B} = \sum_{j=1}^{Dep_i} S_{i,j,k+1} \tag{8}$$

$$DO_{i,k}^{B} = \sum_{j=1}^{Dep_i} DO_{i,j,k} \tag{9}$$

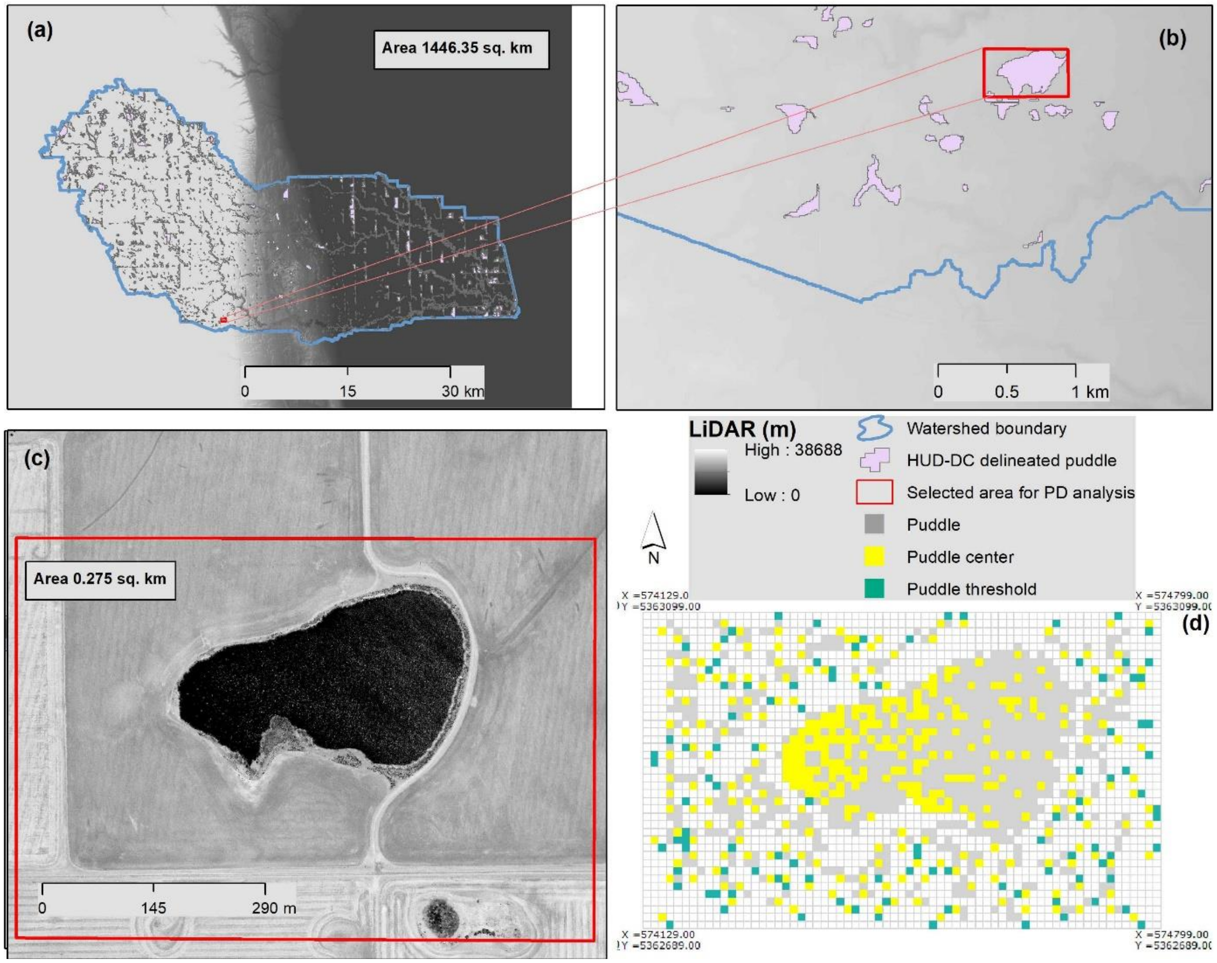

Figure S1: (a) Distribution of depressions identified by HUD-DC within the PR watershed; (b) depressions in a small part of the watershed (in red solid box); (c) LiDAR data showing a depression within the PR watershed; (d) numerous puddles within the selected depression along with puddle center and threshold identified by the puddle delineation (PD, Chu et al., 2010) algorithm.

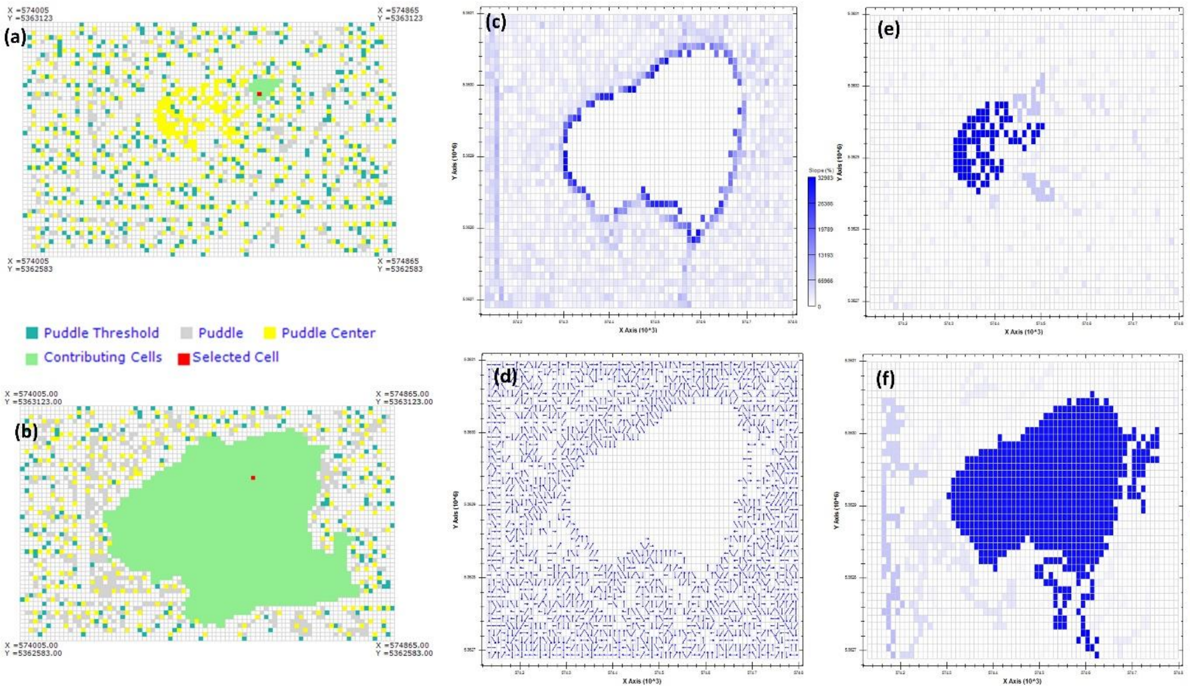

Figure S2: Total 28 puddle level was observed within 0.275 km^2 area (a) puddle with center and threshold in partially filled condition & (b) fully-filled condition (green color indicating contributing area) (c) showing local slope (d) flow direction in fully filled condition (e) flow accumulation in unfilled condition (f) flow accumulation in fully-filled condition

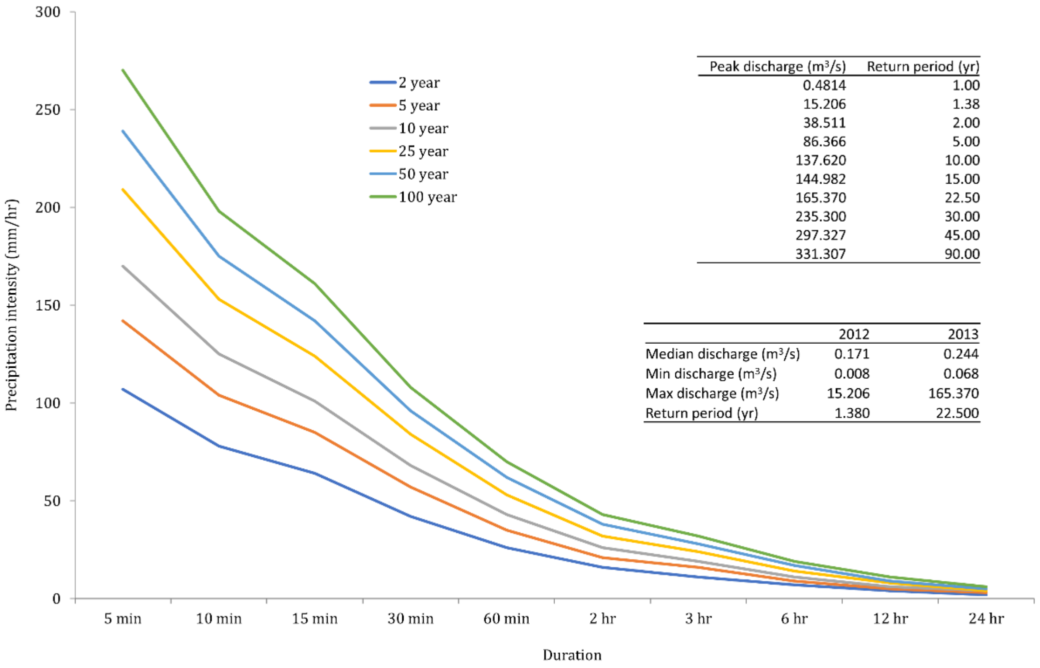

Peak discharge (m^3/s)	Return period (yr)
0.4814	1.00
15.206	1.38
38.511	2.00
86.366	5.00
137.620	10.00
144.982	15.00
165.370	22.50
235.300	30.00
297.327	45.00
331.307	90.00

	2012	2013
Median discharge (m^3/s)	0.171	0.244
Min discharge (m^3/s)	0.008	0.068
Max discharge (m^3/s)	15.206	165.370
Return period (yr)	1.380	22.500

Figure S3: Intensity-Duration-Frequency (IDF) curve of the PR River watershed along with discharge statistics for 2012 & 2013 and return period

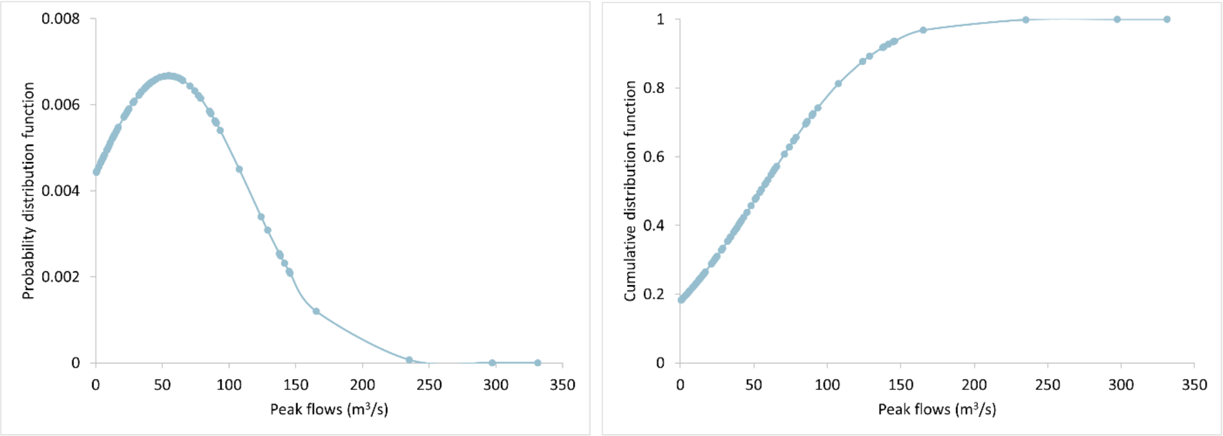

Figure S4: Probability Distribution Function (PDF), Cumulative Distribution Function (CDF) curves

LOADEST (for TN)
A Program to Estimate Constituent Loads
U.S. Geological Survey, Version: MOD48 (March 2013)

PARK RIVER AT GRAFTON, ND (Station #05090000)
Constituent: nitrogen

Constituent Output File Part Ia: Calibration (Load Regression)

Number of Observations : 64
Number of Uncensored Observations: 64
"center" of Decimal Time : 2014.936
"center" of Ln(Q) : 3.9143
Period of record : 2011-2018

Model Evaluation Criteria Based on AMLE Results

Model #	AIC	SPPC
1	1.657	-55.176
2	1.671	-56.723
3	1.588	-54.068
4	1.341	-47.218
5	1.606	-55.697
6	1.368	-49.187
7	1.266	-45.921
8	1.293	-47.856
9	1.326	-49.981

Model # 7 selected

Selected Model:

Ln(Load) = a0 + a1 LnQ + a2 Sin(2 pi dtime) + a3 Cos(2 pi dtime) + a4 dtime
where:
Load = constituent load [kg/d]
LnQ = Ln(Q) - center of Ln(Q)
dtime = decimal time - center of decimal time

Model Coefficients

	a0	a1	a2	a3	a4
AMLE	5.3242	1.0975	0.3717	0.3038	-0.0623
MLE	5.3242	1.0975	0.3717	0.3038	-0.0623
LAD	5.2858	1.0421	0.4575	0.2245	-0.0806

AMLE Regression Statistics

R-Squared [%] : 96.08
Residual Variance : 0.1921
Serial Correlation of Residuals: 0.0174
Prob. Plot Corr. Coeff. (PPCC) : 0.9948
Significance Level of PPCC Test: 7.887E-01

Coeff.	Std.Dev.	t-ratio	P Value
a0	0.0710	74.96	1.585E-65
a1	0.0410	26.79	9.421E-38
a2	0.0970	3.83	1.618E-04
a3	0.1064	2.86	3.996E-03
a4	0.0242	-2.58	8.973E-03

Correlation Between Explanatory Variables

Explanatory variable corresponding to:

	a1	a2	a3
a2	0.3651		
a3	-0.6326	-0.0952	
a4	-0.1432	-0.0979	0.0262

Additional Regression Statistics

MLE Residual Variance: 0.1921
Summary Stats: Est. and Obs. Loads in [KG/DAY]

	Min.	25th Pct	Med.	75th Pct	90th Pct	95th Pct	99th Pct	Max.
Est.	2.51E+00	3.29E+01	1.67E+02	9.48E+02	3.48E+03	6.25E+03	4.52E+04	4.52E+04
Obs.	1.74E+00	3.12E+01	9.72E+01	9.74E+02	2.88E+03	4.84E+03	2.27E+04	2.27E+04
Est/Obs	1.44	1.05	1.72	0.97	1.21	1.29	1.99	1.99

# Residual output file (for TN)

DTIME   decimal time minus "center" of decimal time
LN(CFLOW) natural log of (uncentered) streamflow
F   flag indicating observation is censored (C) or uncensored (U)
CCONC   observed concentration for F=U; 1/2 of the observed concentration for F=C
CCONCAML estimated concentration
YHATC   estimated natural log of concentration
CLOAD   observed load for F=U; 1/2 of the observed load for F=C (units dependent on ULFLAG)
CLOADAML estimated load (units dependent on ULFLAG)
YHAT   estimated natural log of load (where load is in kg/d)
RESID   difference between observed and estimated values of log load (or log concentration)
Z   z-score for residual

#DATE	TIME	DTIME	LN(CFLOW)	F	CCONC	CCONCAML	YHATC	CLOAD	CLOADAML	YHAT	RESID	Z
20110128	935	-3.86E+00	2.48E+00	U	3.49E+00	3.26E+00	1.10E+00	1.02E+02	9.56E+01	4.48E+00	1.54E-01	4.59E-01
20110425	850	-3.62E+00	6.77E+00	U	3.70E+00	3.15E+00	1.06E+00	7.88E+03	6.70E+03	8.72E+00	2.49E-01	6.86E-01
20110510	935	-3.58E+00	5.49E+00	U	2.36E+00	2.45E+00	8.08E-01	1.40E+03	1.45E+03	7.19E+00	5.08E-02	1.74E-01
20110524	852	-3.54E+00	6.58E+00	U	1.39E+00	2.44E+00	8.02E-01	2.45E+03	4.29E+03	8.28E+00	-4.72E-01	-8.98E-01
20110607	1115	-3.50E+00	5.67E+00	U	1.86E+00	2.01E+00	6.11E-01	1.32E+03	1.43E+03	7.18E+00	9.87E-03	-5.77E-02
20110706	1315	-3.42E+00	4.53E+00	U	2.80E+00	1.55E+00	3.48E-01	6.34E+02	3.50E+02	5.77E+00	6.81E-01	1.68E+00
20110808	1000	-3.33E+00	3.39E+00	U	1.26E+00	1.33E+00	1.98E-01	9.19E+01	9.70E+01	4.49E+00	3.32E-02	9.63E-02
20111017	1000	-3.14E+00	2.83E+00	U	1.25E+00	1.80E+00	5.04E-01	5.17E+01	7.45E+01	4.23E+00	-2.81E-01	-6.38E-01
20120110	1315	-2.91E+00	2.09E+00	U	3.26E+00	2.89E+00	9.75E-01	6.46E+01	5.72E+01	3.96E+00	2.07E-01	5.92E-01
20120403	1325	-2.68E+00	4.36E+00	U	4.51E+00	2.78E+00	9.32E-01	8.63E+02	5.31E+02	6.19E+00	5.74E-01	1.09E+00
20120507	1450	-2.59E+00	3.36E+00	U	8.70E-01	1.90E+00	5.56E-01	6.13E+01	1.34E+02	4.81E+00	-6.95E-01	-1.68E+00
20120529	1155	-2.53E+00	2.77E+00	U	1.63E+00	1.51E+00	3.25E-01	6.38E+01	5.91E+01	3.99E+00	1.64E-01	5.02E-01
20120619	940	-2.47E+00	3.00E+00	U	1.41E+00	1.35E+00	2.11E-01	6.90E+01	6.60E+01	4.10E+00	1.32E-01	3.33E-01
20120711	1550	-2.41E+00	1.79E+00	U	1.28E+00	1.09E+00	1.78E-03	1.88E+01	1.60E+01	2.69E+00	2.45E-01	6.38E-01
20120806	1300	-2.34E+00	9.48E-01	U	9.00E-01	9.80E-01	-1.05E-01	5.68E+00	6.19E+00	1.74E+00	-8.35E-04	-9.63E-02
20121030	830	-2.11E+00	1.56E+00	U	8.20E-01	1.67E+00	4.24E-01	9.59E+00	1.95E+01	2.88E+00	-6.23E-01	-1.54E+00
20130130	1040	-1.85E+00	9.93E-01	U	2.31E+00	2.48E+00	8.26E-01	1.53E+01	1.64E+01	2.71E+00	1.15E-02	-1.92E-02
20130501	1000	-1.61E+00	8.67E+00	U	1.59E+00	3.16E+00	1.07E+00	2.27E+04	4.52E+04	1.06E+01	-6.05E-01	-1.33E+00
20130508	1445	-1.59E+00	7.34E+00	U	1.34E+00	2.63E+00	8.79E-01	5.05E+03	9.91E+03	9.11E+00	-5.86E-01	-1.24E+00
20130529	915	-1.53E+00	6.85E+00	U	1.41E+00	2.13E+00	6.66E-01	3.26E+03	4.93E+03	8.41E+00	-3.22E-01	-7.88E-01
20130618	1335	-1.47E+00	5.48E+00	U	1.26E+00	1.63E+00	3.99E-01	7.43E+02	9.64E+02	6.78E+00	-1.68E-01	-4.59E-01
20130710	1520	-1.41E+00	3.94E+00	U	1.07E+00	1.28E+00	1.53E-01	1.34E+02	1.60E+02	4.99E+00	-8.54E-02	-2.93E-01
20130730	930	-1.36E+00	2.92E+00	U	8.60E-01	1.12E+00	2.26E-02	3.89E+01	5.07E+01	3.84E+00	-1.73E-01	-5.02E-01
20131028	1145	-1.11E+00	2.27E+00	U	1.12E+00	1.65E+00	4.14E-01	2.65E+01	3.90E+01	3.58E+00	-3.01E-01	-7.36E-01
20140108	1000	-9.15E-01	8.75E-01	U	2.94E+00	2.25E+00	7.26E-01	1.73E+01	1.32E+01	2.50E+00	3.52E-01	7.36E-01
20140416	1030	-6.47E-01	6.11E+00	U	2.65E+00	2.64E+00	8.82E-01	2.91E+03	2.91E+03	7.88E+00	9.27E-02	2.53E-01
20140423	1100	-6.27E-01	5.36E+00	U	1.94E+00	2.32E+00	7.51E-01	1.01E+03	1.21E+03	7.01E+00	-8.81E-02	-3.33E-01
20140513	1030	-5.73E-01	5.24E+00	U	4.69E+00	1.94E+00	5.72E-01	2.17E+03	8.99E+02	6.71E+00	9.74E-01	2.16E+00
20140624	915	-4.58E-01	5.63E+00	U	2.07E+00	1.51E+00	3.20E-01	1.41E+03	1.03E+03	6.84E+00	4.07E-01	1.02E+00
20140708	1015	-4.19E-01	4.82E+00	U	1.15E+00	1.32E+00	1.84E-01	3.49E+02	4.00E+02	5.90E+00	-4.39E-02	-1.74E-01
20140813	1015	-3.21E-01	1.95E+00	U	8.00E-01	9.68E-01	-1.22E-01	1.38E+01	1.67E+01	2.73E+00	-1.01E-01	-3.75E-01
20141020	1015	-1.34E-01	2.25E+00	U	1.04E+00	1.45E+00	2.85E-01	2.42E+01	3.38E+01	3.43E+00	-2.46E-01	-5.46E-01
20141216	1300	2.21E-02	1.44E+00	U	3.57E+00	2.04E+00	6.24E-01	3.67E+01	2.09E+01	2.95E+00	6.48E-01	1.43E+00
20150414	1550	3.48E-01	3.71E+00	U	8.30E-01	2.00E+00	6.00E-01	8.31E+01	2.00E+02	5.21E+00	-7.87E-01	-1.87E+00
20150420	1050	3.64E-01	3.72E+00	U	6.10E-01	1.90E+00	5.53E-01	6.13E+01	1.91E+02	5.16E+00	-1.05E+00	-2.16E+00
20150518	1505	4.42E-01	6.27E+00	U	3.24E+00	1.93E+00	5.68E-01	4.20E+03	2.51E+03	7.74E+00	6.08E-01	1.24E+00
20150603	930	4.85E-01	4.80E+00	U	1.56E+00	1.49E+00	3.04E-01	4.62E+02	4.40E+02	5.99E+00	1.40E-01	3.75E-01
20150630	1030	5.59E-01	3.26E+00	U	1.03E+00	1.09E+00	-1.16E-03	6.55E+01	6.96E+01	4.15E+00	3.07E-02	5.77E-02
20150825	1330	7.13E-01	2.59E+00	U	9.60E-01	1.00E+00	-9.09E-02	3.12E+01	3.25E+01	3.39E+00	5.01E-02	1.35E-01
20151029	1315	8.91E-01	2.89E+00	U	8.10E-01	1.56E+00	3.59E-01	3.57E+01	6.87E+01	4.14E+00	-5.70E-01	-1.16E+00
20160128	1545	1.14E+00	5.13E-01	U	2.09E+00	1.97E+00	5.92E-01	8.54E+00	8.03E+00	2.00E+00	1.45E-01	4.16E-01
20160411	1145	1.34E+00	3.73E+00	U	1.03E+00	1.92E+00	5.60E-01	1.05E+02	1.96E+02	5.19E+00	-5.30E-01	-1.02E+00
20160426	1130	1.38E+00	5.49E+00	U	4.00E+00	2.01E+00	6.08E-01	2.37E+03	1.19E+03	6.99E+00	7.79E-01	1.87E+00
20160524	945	1.46E+00	4.24E+00	U	1.24E+00	1.42E+00	2.57E-01	2.10E+02	2.40E+02	5.39E+00	-4.16E-02	-1.35E-01
20160628	1145	1.55E+00	4.74E+00	U	1.58E+00	1.20E+00	8.78E-02	4.45E+02	3.37E+02	5.73E+00	3.70E-01	7.88E-01
20160713	1200	1.60E+00	5.05E+00	U	1.91E+00	1.17E+00	6.56E-02	7.29E+02	4.46E+02	6.01E+00	5.82E-01	1.16E+00
20160801	1230	1.65E+00	5.37E+00	U	1.61E+00	1.18E+00	7.49E-02	8.47E+02	6.19E+02	6.34E+00	4.01E-01	8.98E-01
20161016	1115	1.86E+00	4.50E+00	U	2.09E+00	1.54E+00	3.50E-01	4.60E+02	3.38E+02	5.74E+00	3.87E-01	8.41E-01
20170110	1600	2.09E+00	3.27E+00	U	3.26E+00	2.37E+00	7.78E-01	2.09E+02	1.52E+02	4.94E+00	4.04E-01	9.57E-01
20170411	1000	2.34E+00	6.55E+00	U	1.67E+00	2.38E+00	7.79E-01	2.86E+03	4.06E+03	8.22E+00	-2.66E-01	-5.92E-01
20170421	1100	2.37E+00	5.72E+00	U	1.73E+00	2.02E+00	6.15E-01	1.29E+03	1.50E+03	7.23E+00	-6.72E-02	-2.13E-01
20170524	1130	2.46E+00	4.38E+00	U	1.10E+00	1.35E+00	2.13E-01	2.15E+02	2.65E+02	5.49E+00	-1.17E-01	-4.16E-01
20170621	1030	2.53E+00	4.50E+00	U	1.96E+00	1.14E+00	3.83E-02	4.31E+02	2.50E+02	5.43E+00	6.35E-01	1.33E+00
20170718	1240	2.61E+00	2.59E+00	U	9.60E-01	8.55E-01	-2.47E-01	3.12E+01	2.78E+01	3.24E+00	2.06E-01	5.46E-01
20170809	1115	2.67E+00	2.07E+00	U	5.10E-01	8.06E-01	-3.04E-01	9.86E+00	1.56E+01	2.66E+00	-3.69E-01	-8.41E-01
20171012	1100	2.84E+00	1.98E+00	U	5.50E-01	1.10E+00	8.95E-03	9.73E+00	1.95E+01	2.88E+00	-6.07E-01	-1.43E+00
20180105	1430	3.08E+00	1.40E-01	U	2.88E+00	1.62E+00	3.97E-01	8.10E+00	4.55E+00	1.43E+00	6.61E-01	1.54E+00
20180424	1415	3.38E+00	6.01E+00	U	1.88E+00	1.90E+00	5.55E-01	1.88E+03	1.90E+03	7.46E+00	7.58E-02	2.13E-01
20180508	1300	3.41E+00	3.94E+00	U	7.20E-01	1.38E+00	2.36E-01	9.07E+01	1.74E+02	5.07E+00	-5.65E-01	-1.09E+00
20180522	945	3.45E+00	3.87E+00	U	7.00E-01	1.23E+00	1.16E-01	8.19E+01	1.44E+02	4.88E+00	-4.73E-01	-9.57E-01
20180613	1100	3.51E+00	3.56E+00	U	1.04E+00	1.02E+00	-7.02E-02	8.91E+01	8.72E+01	4.38E+00	1.09E-01	2.93E-01
20180710	915	3.59E+00	2.60E+00	U	6.90E-01	8.18E-01	-2.89E-01	2.26E+01	2.68E+01	3.20E+00	-8.24E-02	-2.53E-01
20180828	1100	3.72E+00	1.38E+00	U	7.00E-01	7.42E-01	-3.86E-01	6.78E+00	7.19E+00	1.89E+00	2.89E-02	1.92E-02
20181019	1015	3.86E+00	1.22E-01	U	6.30E-01	9.10E-01	-1.80E-01	1.74E+00	2.51E+00	8.37E-01	-2.82E-01	-6.86E-01

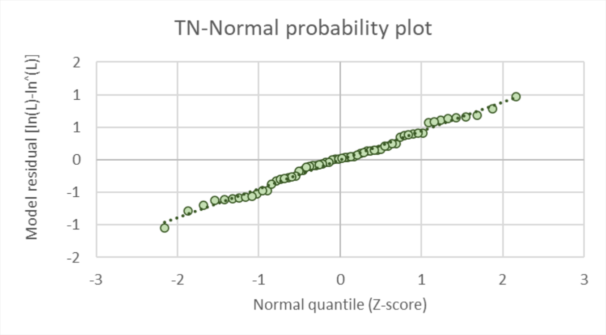

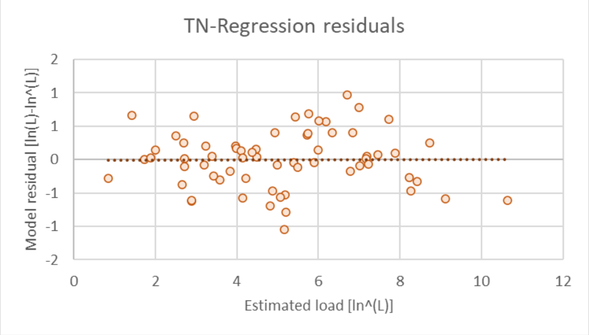

LOADEST (for TP)
A Program to Estimate Constituent Loads
U.S. Geological Survey, Version: MOD48 (March 2013)

PARK RIVER AT GRAFTON, ND (Station #05090000)
Constituent: phosphorus

Constituent Output File Part Ia: Calibration (Load Regression)

Number of Observations : 65
Number of Uncensored Observations: 65
"center" of Decimal Time : 2014.908
"center" of Ln(Q) : 3.9172
Period of record : 2011-2018

Model Evaluation Criteria Based on AMLE Results

Model #	AIC	SPPC
1	1.198	-41.101
2	1.182	-41.680
3	1.215	-42.756
4	1.148	-41.651
5	1.197	-43.267
6	1.074	-40.342
7	1.165	-43.312
8	1.090	-41.947
9	1.122	-44.083

Model # 6 selected

Selected Model:

Ln(Load) = a0 + a1 LnQ + a2 LnQ^2 + a3 Sin(2 pi dtime) + a4 Cos(2 pi dtime)
where:
Load = constituent load [kg/d]
LnQ = Ln(Q) - center of Ln(Q)
dtime = decimal time - center of decimal time

Model Coefficients

	a0	a1	a2	a3	a4
AMLE	2.8543	1.2052	0.0320	-0.2827	-0.0361
MLE	2.8543	1.2052	0.0320	-0.2827	-0.0361
LAD	2.9125	1.2213	0.0227	-0.2786	0.0832

AMLE Regression Statistics

R-Squared [%] : 97.19
Residual Variance : 0.1587
Serial Correlation of Residuals: 0.0581
Prob. Plot Corr. Coeff. (PPCC) : 0.9953
Significance Level of PPCC Test: 8.340E-01

Coeff.	Std.Dev.	t-ratio	P Value
a0	0.0865	32.98	1.260E-43
a1	0.0373	32.27	4.844E-43
a2	0.0124	2.59	8.777E-03
a3	0.0858	-3.30	1.008E-03
a4	0.1033	-0.35	7.161E-01

Correlation Between Explanatory Variables

Explanatory variable corresponding to:

	a1	a2	a3
a2	0.0000		
a3	0.2453	0.2319	
a4	-0.6720	0.2319	-0.0584

Additional Regression Statistics

MLE Residual Variance: 0.1587
Summary Stats: Est. and Obs. Loads in [KG/DAY]

	Min.	25th Pct	Med.	75th Pct	90th Pct	95th Pct	99th Pct	Max.
Est.	2.52E-01	3.15E+00	1.33E+01	1.09E+02	4.10E+02	8.11E+02	1.04E+04	1.04E+04
Obs.	1.66E-01	3.57E+00	1.17E+01	1.07E+02	4.45E+02	6.71E+02	8.34E+03	8.34E+03
Est/Obs	1.52	0.88	1.14	1.03	0.92	1.21	1.25	1.25

# Residual output file (for TP)

DTIME   decimal time minus "center" of decimal time

LN(CFLOW) natural log of (uncentered) streamflow

F   flag indicating observation is censored (C) or uncensored (U)

CCONC   observed concentration for F=U; 1/2 of the observed concentration for F=C

CCONCAML estimated concentration

YHATC   estimated natural log of concentration

CLOAD   observed load for F=U; 1/2 of the observed load for F=C (units dependent on ULFLAG)

CLOADAML estimated load (units dependent on ULFLAG)

YHAT   estimated natural log of load (where load is in kg/d)

RESID   difference between observed and estimated values of log load (or log concentration)

Z   z-score for residual

#DATE	TIME	DTIME	LN(CFLOW)	F	CCONC	CCONCAML	YHATC	CLOAD	CLOADAML	YHAT	RESID	Z
20110128	935	-3.83E+00	2.48E+00	U	1.28E-01	9.28E-02	-2.45E+00	3.76E+00	2.72E+00	9.31E-01	3.93E-01	9.67E-01
20110425	850	-3.59E+00	6.77E+00	U	2.62E-01	3.11E-01	-1.24E+00	5.58E+02	6.62E+02	6.42E+00	-9.86E-02	-1.91E-01
20110510	935	-3.55E+00	5.49E+00	U	1.57E-01	2.15E-01	-1.62E+00	9.30E+01	1.27E+02	4.77E+00	-2.36E-01	-6.04E-01
20110524	852	-3.52E+00	6.58E+00	U	2.08E-01	3.32E-01	-1.18E+00	3.66E+02	5.86E+02	6.30E+00	-3.95E-01	-1.25E+00
20110607	1115	-3.48E+00	5.67E+00	U	1.97E-01	2.60E-01	-1.42E+00	1.40E+02	1.85E+02	5.14E+00	-2.02E-01	-5.15E-01
20110706	1315	-3.40E+00	4.53E+00	U	2.18E-01	2.13E-01	-1.62E+00	4.93E+01	4.83E+01	3.80E+00	9.81E-02	1.52E-01
20110808	1000	-3.31E+00	3.39E+00	U	1.43E-01	1.82E-01	-1.78E+00	1.04E+01	1.33E+01	2.51E+00	-1.66E-01	-3.08E-01
20110808	1000	-3.31E+00	3.39E+00	U	1.97E-01	1.82E-01	-1.78E+00	1.44E+01	1.33E+01	2.51E+00	1.54E-01	3.89E-01
20111017	1000	-3.12E+00	2.83E+00	U	1.12E-01	1.48E-01	-1.98E+00	4.63E+00	6.11E+00	1.74E+00	-2.06E-01	-5.59E-01
20120110	1315	-2.88E+00	2.09E+00	U	1.02E-01	9.34E-02	-2.44E+00	2.02E+00	1.85E+00	5.44E-01	1.60E-01	4.30E-01
20120403	1325	-2.65E+00	4.36E+00	U	1.44E-01	1.36E-01	-2.07E+00	2.76E+01	2.60E+01	3.18E+00	1.33E-01	2.69E-01
20120507	1450	-2.56E+00	3.36E+00	U	7.30E-02	1.28E-01	-2.13E+00	5.14E+00	9.00E+00	2.12E+00	-4.86E-01	-1.34E+00
20120529	1155	-2.50E+00	2.77E+00	U	9.90E-02	1.30E-01	-2.11E+00	3.88E+00	5.09E+00	1.55E+00	-1.99E-01	-4.72E-01
20120619	940	-2.44E+00	3.00E+00	U	1.27E-01	1.48E-01	-1.99E+00	6.21E+00	7.24E+00	1.90E+00	-7.76E-02	-1.14E-01
20120711	1550	-2.38E+00	1.79E+00	U	1.63E-01	1.41E-01	-2.03E+00	2.39E+00	2.07E+00	6.55E-01	2.17E-01	6.04E-01
20120806	1300	-2.31E+00	9.48E-01	U	2.52E-01	1.44E-01	-2.01E+00	1.59E+00	9.06E-01	-1.66E-01	6.31E-01	1.69E+00
20121030	830	-2.08E+00	1.56E+00	U	7.90E-02	1.24E-01	-2.16E+00	9.24E-01	1.45E+00	2.99E-01	-3.79E-01	-1.17E+00
20130130	1040	-1.83E+00	9.93E-01	U	1.05E-01	8.37E-02	-2.55E+00	6.94E-01	5.53E-01	-6.63E-01	2.97E-01	6.98E-01
20130501	1000	-1.58E+00	8.67E+00	U	5.84E-01	7.31E-01	-3.59E-01	8.34E+03	1.04E+04	9.21E+00	-1.79E-01	-3.89E-01
20130508	1445	-1.56E+00	7.34E+00	U	4.79E-01	4.15E-01	-9.48E-01	1.80E+03	1.56E+03	7.29E+00	2.12E-01	5.59E-01
20130529	915	-1.50E+00	6.85E+00	U	3.11E-01	3.78E-01	-1.05E+00	7.20E+02	8.74E+02	6.70E+00	-1.22E-01	-2.69E-01
20130618	1335	-1.45E+00	5.48E+00	U	2.27E-01	2.58E-01	-1.43E+00	1.34E+02	1.52E+02	4.95E+00	-5.32E-02	-3.79E-02
20130710	1520	-1.39E+00	3.94E+00	U	1.44E-01	1.89E-01	-1.74E+00	1.81E+01	2.38E+01	3.09E+00	-1.98E-01	-4.30E-01
20130730	930	-1.33E+00	2.92E+00	U	1.13E-01	1.67E-01	-1.87E+00	5.11E+00	7.55E+00	1.95E+00	-3.14E-01	-7.99E-01
20131028	1145	-1.08E+00	2.27E+00	U	6.80E-02	1.32E-01	-2.10E+00	1.61E+00	3.12E+00	1.07E+00	-5.92E-01	-1.43E+00
20140108	1000	-8.88E-01	8.75E-01	U	6.10E-02	8.84E-02	-2.50E+00	3.58E-01	5.19E-01	-7.26E-01	-3.01E-01	-7.48E-01
20140416	1030	-6.19E-01	6.11E+00	U	4.10E-01	2.36E-01	-1.52E+00	4.50E+02	2.59E+02	5.48E+00	6.29E-01	1.55E+00
20140423	1100	-6.00E-01	5.36E+00	U	2.40E-01	1.91E-01	-1.73E+00	1.25E+02	9.95E+01	4.52E+00	3.04E-01	7.48E-01
20140513	1030	-5.45E-01	5.24E+00	U	9.00E-02	2.02E-01	-1.67E+00	4.16E+01	9.36E+01	4.46E+00	-7.34E-01	-1.69E+00
20140624	915	-4.30E-01	5.63E+00	U	1.90E-01	2.77E-01	-1.36E+00	1.29E+02	1.88E+02	5.16E+00	-3.00E-01	-6.98E-01
20140708	1015	-3.92E-01	4.82E+00	U	1.80E-01	2.31E-01	-1.54E+00	5.46E+01	7.01E+01	4.17E+00	-1.74E-01	-3.48E-01
20140813	1015	-2.93E-01	1.95E+00	U	1.00E-01	1.52E-01	-1.96E+00	1.72E+00	2.63E+00	8.93E-01	-3.47E-01	-9.67E-01
20141020	1015	-1.07E-01	2.25E+00	U	1.40E-01	1.37E-01	-2.06E+00	3.26E+00	3.18E+00	1.09E+00	9.64E-02	1.14E-01
20141216	1300	4.94E-02	1.44E+00	U	1.60E-01	9.85E-02	-2.39E+00	1.64E+00	1.01E+00	-6.07E-02	5.58E-01	1.34E+00
20150414	1550	3.76E-01	3.71E+00	U	9.00E-02	1.23E-01	-2.17E+00	9.01E+00	1.23E+01	2.43E+00	-2.36E-01	-6.51E-01
20150420	1050	3.92E-01	3.72E+00	U	8.00E-02	1.26E-01	-2.15E+00	8.04E+00	1.26E+01	2.46E+00	-3.78E-01	-1.10E+00
20150518	1505	4.69E-01	6.27E+00	U	3.40E-01	2.89E-01	-1.32E+00	4.41E+02	3.75E+02	5.85E+00	2.37E-01	6.51E-01
20150603	930	5.12E-01	4.80E+00	U	1.80E-01	1.98E-01	-1.70E+00	5.33E+01	5.87E+01	4.00E+00	-1.97E-02	3.79E-02
20150630	1030	5.86E-01	3.26E+00	U	1.40E-01	1.61E-01	-1.90E+00	8.91E+00	1.02E+01	2.25E+00	-6.38E-02	-7.58E-02
20150825	1330	7.40E-01	2.59E+00	U	2.10E-01	1.63E-01	-1.89E+00	6.83E+00	5.30E+00	1.59E+00	3.29E-01	7.99E-01
20151029	1315	9.18E-01	2.89E+00	U	1.30E-01	1.41E-01	-2.03E+00	5.72E+00	6.22E+00	1.76E+00	-1.36E-02	7.58E-02
20160128	1545	1.17E+00	5.13E-01	U	1.20E-01	8.36E-02	-2.55E+00	4.90E-01	3.41E-01	-1.14E+00	4.28E-01	1.03E+00
20160411	1145	1.37E+00	3.73E+00	U	8.00E-02	1.22E-01	-2.18E+00	8.18E+00	1.25E+01	2.45E+00	-3.49E-01	-1.03E+00
20160426	1130	1.41E+00	5.49E+00	U	1.80E-01	2.02E-01	-1.68E+00	1.07E+02	1.19E+02	4.71E+00	-3.80E-02	1.01E-07
20160524	945	1.49E+00	4.24E+00	U	2.90E-01	1.65E-01	-1.88E+00	4.91E+01	2.80E+01	3.25E+00	6.39E-01	1.88E+00
20160628	1145	1.58E+00	4.74E+00	U	2.90E-01	2.19E-01	-1.59E+00	8.16E+01	6.16E+01	4.04E+00	3.57E-01	8.52E-01
20160713	1200	1.62E+00	5.05E+00	U	2.80E-01	2.50E-01	-1.46E+00	1.07E+02	9.55E+01	4.48E+00	1.88E-01	5.15E-01
20160801	1230	1.68E+00	5.37E+00	U	4.90E-01	2.86E-01	-1.32E+00	2.58E+02	1.51E+02	4.94E+00	6.10E-01	1.43E+00
20161016	1115	1.88E+00	4.50E+00	U	2.20E-01	2.02E-01	-1.66E+00	4.84E+01	4.44E+01	3.73E+00	1.50E-01	3.48E-01
20170110	1600	2.12E+00	3.27E+00	U	1.60E-01	1.08E-01	-2.30E+00	1.03E+01	6.91E+00	1.86E+00	4.63E-01	1.10E+00
20170411	1000	2.37E+00	6.55E+00	U	2.90E-01	2.71E-01	-1.38E+00	4.96E+02	4.63E+02	6.06E+00	1.43E-01	3.08E-01
20170421	1100	2.39E+00	5.72E+00	U	1.80E-01	2.11E-01	-1.63E+00	1.34E+02	1.57E+02	4.98E+00	-8.47E-02	-1.52E-01
20170524	1130	2.48E+00	4.38E+00	U	7.00E-02	1.70E-01	-1.85E+00	1.37E+01	3.33E+01	3.43E+00	-8.13E-01	-1.88E+00
20170621	1030	2.56E+00	4.50E+00	U	2.20E-01	2.00E-01	-1.69E+00	4.84E+01	4.39E+01	3.71E+00	1.73E-01	4.72E-01
20170718	1240	2.64E+00	2.59E+00	U	1.60E-01	1.55E-01	-1.94E+00	5.21E+00	5.06E+00	1.55E+00	1.05E-01	1.91E-01
20170809	1115	2.70E+00	2.07E+00	U	1.60E-01	1.53E-01	-1.95E+00	3.09E+00	2.97E+00	1.01E+00	1.17E-01	2.29E-01
20171012	1100	2.87E+00	1.98E+00	U	5.00E-02	1.38E-01	-2.06E+00	8.84E-01	2.44E+00	8.18E-01	-9.40E-01	-2.17E+00
20180105	1430	3.10E+00	1.40E-01	U	6.00E-02	8.96E-02	-2.48E+00	1.69E-01	2.52E-01	-1.44E+00	-3.37E-01	-9.08E-01
20180424	1415	3.40E+00	6.01E+00	U	3.60E-01	2.36E-01	-1.52E+00	3.59E+02	2.36E+02	5.39E+00	4.98E-01	1.17E+00
20180508	1300	3.44E+00	3.94E+00	U	1.20E-01	1.43E-01	-2.02E+00	1.51E+01	1.80E+01	2.82E+00	-9.93E-02	-2.29E-01
20180522	945	3.48E+00	3.87E+00	U	1.00E-01	1.51E-01	-1.97E+00	1.17E+01	1.76E+01	2.79E+00	-3.34E-01	-8.52E-01
20180613	1100	3.54E+00	3.56E+00	U	2.10E-01	1.58E-01	-1.92E+00	1.80E+01	1.35E+01	2.53E+00	3.62E-01	9.08E-01
20180710	915	3.61E+00	2.60E+00	U	2.40E-01	1.52E-01	-1.96E+00	7.87E+00	4.97E+00	1.53E+00	5.34E-01	1.25E+00
20180828	1100	3.75E+00	1.38E+00	U	3.50E-01	1.47E-01	-1.99E+00	3.39E+00	1.43E+00	2.82E-01	9.39E-01	2.17E+00
20181019	1015	3.89E+00	1.22E-01	U	6.00E-02	1.28E-01	-2.12E+00	1.66E-01	3.53E-01	-1.11E+00	-6.90E-01	-1.55E+00

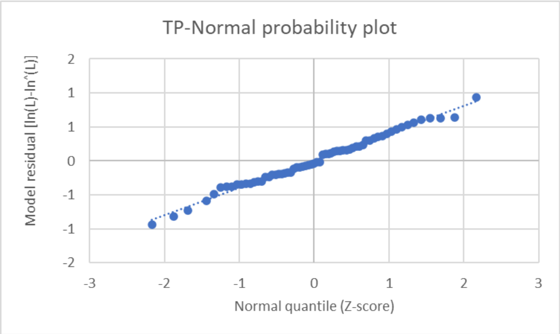

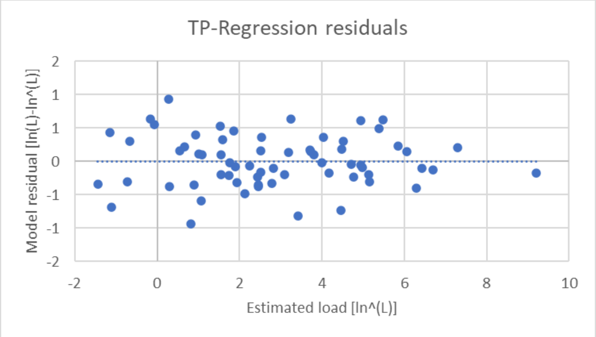

Table: Flood frequency analysis

Flood record for a 90-year period

Used formula: California Formula m/n

m	PeakFlowQp_cms	Frequency F=m/90	Return Period Tr=1/F	Normal Distribution-CDF	Normal Distribution-PDF
1	331.3071099	0.0111	90.00	0.999998166	1.48163E-07
2	297.3268935	0.0222	45.00	0.999975559	1.75137E-06
3	235.0298301	0.0333	30.00	0.998731157	7.00552E-05
4	165.3703865	0.0444	22.50	0.968098584	0.001197584
5	145.8317621	0.0556	18.00	0.936584052	0.002080712
6	144.9822566	0.0667	15.00	0.934797223	0.002126132
7	141.584235	0.0778	12.86	0.92725707	0.00231327
8	138.4693818	0.0889	11.25	0.919774722	0.002492166
9	137.6198764	0.1000	10.00	0.917636432	0.002542115
10	128.8416539	0.1111	9.00	0.89297507	0.003084003
11	124.0277899	0.1222	8.18	0.877378278	0.003397501
12	107.6040186	0.1333	7.50	0.812526541	0.00450207
13	93.4455951	0.1444	6.92	0.742284513	0.005401228
14	90.33074193	0.1556	6.43	0.725179961	0.005579829
15	90.33074193	0.1667	6.00	0.725179961	0.005579829
16	89.76440499	0.1778	5.63	0.722010972	0.005611294
17	89.48123652	0.1889	5.29	0.720419818	0.005626904
18	86.36638335	0.2000	5.00	0.702631516	0.005792916
19	85.80004641	0.2111	4.74	0.699342542	0.005821924
20	85.23370947	0.2222	4.50	0.696037247	0.005850552
21	78.72083466	0.2333	4.29	0.656926802	0.006150264
22	77.02182384	0.2444	4.09	0.646418622	0.006218792
23	74.19013914	0.2556	3.91	0.628658023	0.006323346
24	70.7921175	0.2667	3.75	0.606981145	0.006432056
25	65.41191657	0.2778	3.60	0.57199672	0.006564495
26	64.27924269	0.2889	3.46	0.564548937	0.006585921
27	63.14656881	0.3000	3.33	0.557078187	0.006605046
28	61.73072646	0.3111	3.21	0.547711443	0.006625684
29	59.4653787	0.3222	3.10	0.532671424	0.006651075
30	58.04953635	0.3333	3.00	0.523246284	0.006662133
31	57.48319941	0.3444	2.90	0.519472286	0.006665515
32	55.21785165	0.3556	2.81	0.504362216	0.006673067
33	55.21785165	0.3667	2.73	0.504362216	0.006673067
34	54.08517777	0.3778	2.65	0.496803476	0.006673252
35	51.81983001	0.3889	2.57	0.481692151	0.006666438
36	51.53666154	0.4000	2.50	0.479804638	0.006664914
37	50.9703246	0.4111	2.43	0.476031013	0.006661418
38	48.1386399	0.4222	2.37	0.457201848	0.006635027
39	45.3069552	0.4333	2.31	0.438468287	0.00659393
40	43.04160744	0.4444	2.25	0.423578043	0.006550644
41	41.34259662	0.4556	2.20	0.412480348	0.006512227
42	41.34259662	0.4667	2.14	0.412480348	0.006512227
43	40.49309121	0.4778	2.09	0.406957047	0.006491136
44	39.6435858	0.4889	2.05	0.401452188	0.006468807
45	38.51091192	0.5000	2.00	0.394142874	0.006437133
46	37.94457498	0.5111	1.96	0.390501975	0.006420489
47	37.37823804	0.5222	1.91	0.386870653	0.006403314
48	36.24556416	0.5333	1.88	0.379637936	0.006367387
49	34.26338487	0.5444	1.84	0.367082832	0.006299554
50	33.9802164	0.5556	1.80	0.365300438	0.006289358
51	32.56437405	0.5667	1.76	0.356432736	0.006236528
52	32.28120558	0.5778	1.73	0.354668293	0.006225596
53	32.28120558	0.5889	1.70	0.354668293	0.006225596
54	28.88318394	0.6000	1.67	0.333747325	0.006085239
55	28.06199538	0.6111	1.64	0.328765068	0.006048864
56	27.97704484	0.6222	1.61	0.328251376	0.006045049
57	24.91882536	0.6333	1.58	0.30998094	0.005901336
58	24.60734004	0.6444	1.55	0.308145141	0.005886028
59	23.78615148	0.6556	1.53	0.303328352	0.005845098
60	22.99327976	0.6667	1.50	0.298709862	0.00580481
61	21.94555643	0.6778	1.48	0.292656396	0.005750446
62	21.23763525	0.6889	1.45	0.288598752	0.005713008
63	16.93347451	0.7000	1.43	0.264516606	0.005474031
64	15.99901856	0.7111	1.41	0.259426656	0.00541977
65	15.20614684	0.7222	1.38	0.25514793	0.005373122
66	14.1584235	0.7333	1.36	0.249551025	0.005310663
67	13.98852242	0.7444	1.34	0.248649605	0.00530045
68	13.16733386	0.7556	1.32	0.2443173	0.005250764
69	13.11070016	0.7667	1.30	0.244020028	0.005247318
70	12.54436322	0.7778	1.29	0.241058061	0.005212726
71	11.12852087	0.7889	1.27	0.233739438	0.005125227
72	10.9019861	0.8000	1.25	0.232579995	0.005111099
73	9.797629062	0.8111	1.23	0.226973744	0.005041739
74	8.664955182	0.8222	1.22	0.221303759	0.004969816
75	8.268519324	0.8333	1.20	0.219338567	0.004944467
76	8.240202477	0.8444	1.18	0.219198581	0.004942653
77	6.541191657	0.8556	1.17	0.210893868	0.004833037
78	5.974854717	0.8667	1.15	0.208167171	0.00479618
79	5.38020093	0.8778	1.14	0.20532665	0.004757323
80	4.53069552	0.8889	1.13	0.201308946	0.004701552
81	3.68119011	0.9000	1.11	0.197338743	0.004645496
82	3.652873263	0.9111	1.10	0.197207224	0.004643623
83	3.652873263	0.9222	1.08	0.197207224	0.004643623
84	2.26534776	0.9333	1.07	0.190827912	0.004551508
85	2.248357652	0.9444	1.06	0.190750591	0.004550376
86	1.13267388	0.9556	1.05	0.185715335	0.004475881
87	0.906139104	0.9667	1.03	0.18470311	0.004460714
88	0.622970634	0.9778	1.02	0.183442662	0.004441739
89	0.538020093	0.9889	1.01	0.183065576	0.004436043
90	0.481386399	1.0000	1.00	0.182814454	0.004432245
**Mean**	**54.56417297**				
**Max**	**331.30710990**				
**Min**	**0.48138640**				
**Median**	**38.22774345**				
**Skewne**	**2.36667215**				
**Standa**	**59.78037114**				

Qp_cms	Tr_yr
0.48	1.00
15.21	1.38
38.51	2.00
86.37	5.00
137.62	10.00
144.98	15.00
165.37	22.50
235.03	30.00
297.33	45.00
331.31	90.00

Row Labels	Max of discharge_cfs
1934	393
1935	443
1936	1140
1937	306
1938	291
1939	130
1940	190
1941	1830
1942	3190
1943	1400
1944	494
1945	1140
1946	1150
1947	500
1948	10500
1949	2500
1950	11700
1951	1520
1952	160
1953	80
1954	463
1955	1950
1956	3800
1957	1200
1958	32
1959	1020
1960	2720
1961	19
1962	4550
1963	292
1964	2050
1965	5120
1966	2620
1967	2230
1968	565
1969	4890
1970	1460
1971	3050
1972	1910
1973	211
1974	3030
1975	880
1976	1700
1977	17
1978	3170
1979	8300
1980	385
1981	465
1982	1320
1983	1360
1984	231
1985	840
1986	812
1987	3160
1988	129
1989	129
1990	22
1991	40
1992	991
1993	2310
1994	750
1995	1950
1996	3300
1997	5150
1998	3190
1999	2180
2000	346
2001	1820
2002	1430
2003	869
2004	5000
2005	1800
2006	2030
2007	2100
2008	775
2009	4860
2010	2270
2011	3010
2012	537
2013	5840
2014	2780
2015	988
2016	1210
2017	4380
2018	598
2019	1600
2020	1280
2021	79.4
2022	1460
2023	1340
**Grand Total**	**11700**

Flood record for a 90-year period		
**Year**	**PeakFlowQp_cfs**	**PeakFlowQp_cms**
1934	393	11.12852087
1935	443	12.54436322
1936	1140	32.28120558
1937	306	8.664955182
1938	291	8.240202477
1939	130	3.68119011
1940	190	5.38020093
1941	1830	51.81983001
1942	3190	90.33074193
1943	1400	39.6435858
1944	494	13.98852242
1945	1140	32.28120558
1946	1150	32.56437405
1947	500	14.1584235
1948	10500	297.3268935
1949	2500	70.7921175
1950	11700	331.3071099
1951	1520	43.04160744
1952	160	4.53069552
1953	80	2.26534776
1954	463	13.11070016
1955	1950	55.21785165
1956	3800	107.6040186
1957	1200	33.9802164
1958	32	0.906139104
1959	1020	28.88318394
1960	2720	77.02182384
1961	19	0.538020093
1962	4550	128.8416539
1963	292	8.268519324
1964	2050	58.04953635
1965	5120	144.9822566
1966	2620	74.19013914
1967	2230	63.14656881
1968	565	15.99901856
1969	4890	138.4693818
1970	1460	41.34259662
1971	3050	86.36638335
1972	1910	54.08517777
1973	211	5.974854717
1974	3030	85.80004641
1975	880	24.91882536
1976	1700	48.1386399
1977	17	0.481386399
1978	3170	89.76440499
1979	8300	235.0298301
1980	385	10.9019861
1981	465	13.16733386
1982	1320	37.37823804
1983	1360	38.51091192
1984	231	6.541191657
1985	840	23.78615148
1986	812	22.99327976
1987	3160	89.48123652
1988	129	3.652873263
1989	129	3.652873263
1990	22	0.622970634
1991	40	1.13267388
1992	991	28.06199538
1993	2310	65.41191657
1994	750	21.23763525
1995	1950	55.21785165
1996	3300	93.4455951
1997	5150	145.8317621
1998	3190	90.33074193
1999	2180	61.73072646
2000	346	9.797629062
2001	1820	51.53666154
2002	1430	40.49309121
2003	869	24.60734004
2004	5000	141.584235
2005	1800	50.9703246
2006	2030	57.48319941
2007	2100	59.4653787
2008	775	21.94555643
2009	4860	137.6198764
2010	2270	64.27924269
2011	3010	85.23370947
2012	537	15.20614684
2013	5840	165.3703865
2014	2780	78.72083466
2015	988	27.97704484
2016	1210	34.26338487
2017	4380	124.0277899
2018	598	16.93347451
2019	1600	45.3069552
2020	1280	36.24556416
2021	79.4	2.248357652
2022	1460	41.34259662
2023	1340	37.94457498

	cms
**Mean**	**54.56**
**Max**	**331.31**
**Min**	**0.48**
**Median**	**38.23**
**Skewness**	**2.37**
**Standard Deviation**	**59.78**

Table: Thirty (30) year discharge at the PR river watershed

Date	discharge cfs	Date	dis. cfs	Date	dis. cfs	Date	dis. cfs	Date	dis. cfs	Date	dis. cfs	Date	dis. cfs	Date	dis. cfs	Date	dis. cfs	Date	dis. cfs
5/1/1931	22	8/8/1931	0.2	11/15/1931	0.4	2/22/1932	2	5/31/1932	7.3	9/7/1932	0	12/15/1932	0	3/24/1933	40	7/1/1933	0.5	10/8/1933	0
5/2/1931	17	8/9/1931	0.2	11/16/1931	0.4	2/23/1932	2	6/1/1932	7.3	9/8/1932	0	12/16/1932	0	3/25/1933	40	7/2/1933	0.5	10/9/1933	0
5/3/1931	16	8/10/1931	0	11/17/1931	0.4	2/24/1932	2	6/2/1932	13	9/9/1932	0	12/17/1932	0	3/26/1933	40	7/3/1933	0.5	10/10/1933	0
5/4/1931	16	8/11/1931	0	11/18/1931	0.4	2/25/1932	2	6/3/1932	10	9/10/1932	0	12/18/1932	0	3/27/1933	40	7/4/1933	0.5	10/11/1933	0
5/5/1931	16	8/12/1931	0.2	11/19/1931	0.4	2/26/1932	2	6/4/1932	10	9/11/1932	0	12/19/1932	0	3/28/1933	40	7/5/1933	0.5	10/12/1933	0
5/6/1931	16	8/13/1931	0.5	11/20/1931	0.4	2/27/1932	2	6/5/1932	8.1	9/12/1932	0	12/20/1932	0	3/29/1933	40	7/6/1933	0.5	10/13/1933	0
5/7/1931	17	8/14/1931	0.3	11/21/1931	0.4	2/28/1932	2	6/6/1932	7.3	9/13/1932	0	12/21/1932	0	3/30/1933	40	7/7/1933	0.5	10/14/1933	0
5/8/1931	16	8/15/1931	0.2	11/22/1931	0.4	2/29/1932	2	6/7/1932	12	9/14/1932	0	12/22/1932	0	3/31/1933	40	7/8/1933	0.5	10/15/1933	0
5/9/1931	18	8/16/1931	0.1	11/23/1931	0.4	3/1/1932	15	6/8/1932	8.1	9/15/1932	0	12/23/1932	0	4/1/1933	1500	7/9/1933	0.5	10/16/1933	0
5/10/1931	18	8/17/1931	0	11/24/1931	0.4	3/2/1932	15	6/9/1932	6.5	9/16/1932	0	12/24/1932	0	4/2/1933	2010	7/10/1933	0.5	10/17/1933	0
5/11/1931	18	8/18/1931	0.2	11/25/1931	0.4	3/3/1932	15	6/10/1932	5.9	9/17/1932	0	12/25/1932	0	4/3/1933	1840	7/11/1933	0.5	10/18/1933	0
5/12/1931	17	8/19/1931	0.1	11/26/1931	0.4	3/4/1932	15	6/11/1932	5.3	9/18/1932	0	12/26/1932	0	4/4/1933	1180	7/12/1933	0.5	10/19/1933	0
5/13/1931	16	8/20/1931	0.1	11/27/1931	0.4	3/5/1932	15	6/12/1932	8.1	9/19/1932	0	12/27/1932	0	4/5/1933	837	7/13/1933	0.5	10/20/1933	0
5/14/1931	12	8/21/1931	0	11/28/1931	0.4	3/6/1932	15	6/13/1932	10	9/20/1932	0	12/28/1932	0	4/6/1933	456	7/14/1933	0.5	10/21/1933	0
5/15/1931	12	8/22/1931	0	11/29/1931	0.4	3/7/1932	15	6/14/1932	6.5	9/21/1932	0	12/29/1932	0	4/7/1933	381	7/15/1933	0.5	10/22/1933	0
5/16/1931	10	8/23/1931	0	11/30/1931	0.4	3/8/1932	15	6/15/1932	2.9	9/22/1932	0	12/30/1932	0	4/8/1933	273	7/16/1933	0.5	10/23/1933	0
5/17/1931	12	8/24/1931	0	12/1/1931	0.1	3/9/1932	15	6/16/1932	1.6	9/23/1932	0	12/31/1932	0	4/9/1933	285	7/17/1933	0.5	10/24/1933	0
5/18/1931	12	8/25/1931	0	12/2/1931	0.1	3/10/1932	15	6/17/1932	21	9/24/1932	0	1/1/1933	0	4/10/1933	262	7/18/1933	0.5	10/25/1933	0
5/19/1931	12	8/26/1931	0	12/3/1931	0.1	3/11/1932	15	6/18/1932	8.1	9/25/1932	0	1/2/1933	0	4/11/1933	229	7/19/1933	0.5	10/26/1933	0
5/20/1931	11	8/27/1931	0	12/4/1931	0.1	3/12/1932	15	6/19/1932	2.3	9/26/1932	0	1/3/1933	0	4/12/1933	185	7/20/1933	0.5	10/27/1933	0
5/21/1931	9	8/28/1931	0.5	12/5/1931	0.1	3/13/1932	15	6/20/1932	2.6	9/27/1932	0	1/4/1933	0	4/13/1933	143	7/21/1933	0.5	10/28/1933	0
5/22/1931	9	8/29/1931	0.4	12/6/1931	0.1	3/14/1932	15	6/21/1932	2.9	9/28/1932	0	1/5/1933	0	4/14/1933	112	7/22/1933	0.5	10/29/1933	0
5/23/1931	9	8/30/1931	0.3	12/7/1931	0.1	3/15/1932	15	6/22/1932	1.8	9/29/1932	0	1/6/1933	0	4/15/1933	112	7/23/1933	0.5	10/30/1933	0
5/24/1931	7.3	8/31/1931	0.2	12/8/1931	0.1	3/16/1932	15	6/23/1932	1.2	9/30/1932	0	1/7/1933	0	4/16/1933	207	7/24/1933	0.5	10/31/1933	0
5/25/1931	6.5	9/1/1931	0.1	12/9/1931	0.1	3/17/1932	15	6/24/1932	1.3	10/1/1932	1	1/8/1933	0	4/17/1933	357	7/25/1933	0.5	11/1/1933	0
5/26/1931	8.1	9/2/1931	0.1	12/10/1931	0.1	3/18/1932	15	6/25/1932	1.1	10/2/1932	1	1/9/1933	0	4/18/1933	443	7/26/1933	0.5	11/2/1933	0
5/27/1931	6.5	9/3/1931	0.1	12/11/1931	0.1	3/19/1932	15	6/26/1932	0.6	10/3/1932	1	1/10/1933	0	4/19/1933	369	7/27/1933	0.5	11/3/1933	0
5/28/1931	7.3	9/4/1931	0.1	12/12/1931	0.1	3/20/1932	15	6/27/1932	0.5	10/4/1932	1	1/11/1933	0	4/20/1933	285	7/28/1933	0.5	11/4/1933	0
5/29/1931	6.5	9/5/1931	0.1	12/13/1931	0.1	3/21/1932	15	6/28/1932	0.4	10/5/1932	1	1/12/1933	0	4/21/1933	185	7/29/1933	0.5	11/5/1933	0
5/30/1931	6.5	9/6/1931	0.1	12/14/1931	0.1	3/22/1932	15	6/29/1932	0.4	10/6/1932	1	1/13/1933	0	4/22/1933	143	7/30/1933	0.5	11/6/1933	0
5/31/1931	6.5	9/7/1931	0.1	12/15/1931	0.1	3/23/1932	15	6/30/1932	0.7	10/7/1932	1	1/14/1933	0	4/23/1933	112	7/31/1933	0.5	11/7/1933	0
6/1/1931	6.2	9/8/1931	0.1	12/16/1931	0.1	3/24/1932	15	7/1/1932	0.4	10/8/1932	1	1/15/1933	0	4/24/1933	92	8/1/1933	0.2	11/8/1933	0
6/2/1931	6.2	9/9/1931	0.1	12/17/1931	0.1	3/25/1932	15	7/2/1932	0.4	10/9/1932	1	1/16/1933	0	4/25/1933	77	8/2/1933	0.2	11/9/1933	0
6/3/1931	6.9	9/10/1931	0.1	12/18/1931	0.1	3/26/1932	15	7/3/1932	0.6	10/10/1932	1	1/17/1933	0	4/26/1933	68	8/3/1933	0.2	11/10/1933	0
6/4/1931	6.2	9/11/1931	0.1	12/19/1931	0.1	3/27/1932	15	7/4/1932	1	10/11/1932	1	1/18/1933	0	4/27/1933	59	8/4/1933	0.2	11/11/1933	0
6/5/1931	6.2	9/12/1931	0.1	12/20/1931	0.1	3/28/1932	15	7/5/1932	1	10/12/1932	1	1/19/1933	0	4/28/1933	51	8/5/1933	0.2	11/12/1933	0
6/6/1931	6.2	9/13/1931	0.1	12/21/1931	0.1	3/29/1932	15	7/6/1932	1	10/13/1932	1	1/20/1933	0	4/29/1933	46	8/6/1933	0.2	11/13/1933	0
6/7/1931	5	9/14/1931	0.1	12/22/1931	0.1	3/30/1932	15	7/7/1932	1.1	10/14/1932	1	1/21/1933	0	4/30/1933	42	8/7/1933	0.2	11/14/1933	0
6/8/1931	3.9	9/15/1931	0.1	12/23/1931	0.1	3/31/1932	15	7/8/1932	0.7	10/15/1932	1	1/22/1933	0	5/1/1933	38	8/8/1933	0.2	11/15/1933	0
6/9/1931	3.9	9/16/1931	0.1	12/24/1931	0.1	4/1/1932	80	7/9/1932	0.6	10/16/1932	1	1/23/1933	0	5/2/1933	34	8/9/1933	0.2	11/16/1933	0
6/10/1931	3.5	9/17/1931	0.1	12/25/1931	0.1	4/2/1932	70	7/10/1932	0.3	10/17/1932	1	1/24/1933	0	5/3/1933	30	8/10/1933	0.2	11/17/1933	0
6/11/1931	3.9	9/18/1931	0.1	12/26/1931	0.1	4/3/1932	70	7/11/1932	0.6	10/18/1932	1	1/25/1933	0	5/4/1933	25	8/11/1933	0.2	11/18/1933	0
6/12/1931	3.9	9/19/1931	0.1	12/27/1931	0.1	4/4/1932	60	7/12/1932	0.3	10/19/1932	1	1/26/1933	0	5/5/1933	22	8/12/1933	0.2	11/19/1933	0
6/13/1931	3.3	9/20/1931	0.1	12/28/1931	0.1	4/5/1932	79	7/13/1932	0.2	10/20/1932	1	1/27/1933	0	5/6/1933	21	8/13/1933	0.2	11/20/1933	0
6/14/1931	3	9/21/1931	0.1	12/29/1931	0.1	4/6/1932	153	7/14/1932	0.1	10/21/1932	1	1/28/1933	0	5/7/1933	19	8/14/1933	0.2	11/21/1933	0
6/15/1931	2.6	9/22/1931	0.1	12/30/1931	0.1	4/7/1932	185	7/15/1932	0	10/22/1932	1	1/29/1933	0	5/8/1933	21	8/15/1933	0.2	11/22/1933	0
6/16/1931	2.1	9/23/1931	0.1	12/31/1931	0.1	4/8/1932	526	7/16/1932	0	10/23/1932	1	1/30/1933	0	5/9/1933	24	8/16/1933	0.2	11/23/1933	0
6/17/1931	1.6	9/24/1931	0.1	1/1/1932	0	4/9/1932	608	7/17/1932	0	10/24/1932	1	1/31/1933	0	5/10/1933	25	8/17/1933	0.2	11/24/1933	0
6/18/1931	1.8	9/25/1931	0.1	1/2/1932	0	4/10/1932	738	7/18/1932	0	10/25/1932	1	2/1/1933	0	5/11/1933	22	8/18/1933	0.2	11/25/1933	0
6/19/1931	1.6	9/26/1931	0.1	1/3/1932	0	4/11/1932	705	7/19/1932	0	10/26/1932	1	2/2/1933	0	5/12/1933	21	8/19/1933	0.2	11/26/1933	0
6/20/1931	1.2	9/27/1931	0.1	1/4/1932	0	4/12/1932	487	7/20/1932	0	10/27/1932	1	2/3/1933	0	5/13/1933	19	8/20/1933	0.2	11/27/1933	0
6/21/1931	1.2	9/28/1931	0.1	1/5/1932	0	4/13/1932	292	7/21/1932	0	10/28/1932	1	2/4/1933	0	5/14/1933	22	8/21/1933	0.2	11/28/1933	0
6/22/1931	1.6	9/29/1931	0.1	1/6/1932	0	4/14/1932	192	7/22/1932	0	10/29/1932	1	2/5/1933	0	5/15/1933	18	8/22/1933	0.2	11/29/1933	0
6/23/1931	1.3	9/30/1931	0.1	1/7/1932	0	4/15/1932	271	7/23/1932	0	10/30/1932	1	2/6/1933	0	5/16/1933	16	8/23/1933	0.2	11/30/1933	0
6/24/1931	1.3	10/1/1931	0.1	1/8/1932	0	4/16/1932	125	7/24/1932	0	10/31/1932	1	2/7/1933	0	5/17/1933	16	8/24/1933	0.2	12/1/1933	0
6/25/1931	1.1	10/2/1931	0.1	1/9/1932	0	4/17/1932	102	7/25/1932	0	11/1/1932	0.8	2/8/1933	0	5/18/1933	15	8/25/1933	0.2	12/2/1933	0
6/26/1931	1	10/3/1931	0.1	1/10/1932	0	4/18/1932	95	7/26/1932	0	11/2/1932	0.8	2/9/1933	0	5/19/1933	14	8/26/1933	0.2	12/3/1933	0
6/27/1931	1.1	10/4/1931	0.1	1/11/1932	0	4/19/1932	95	7/27/1932	0	11/3/1932	0.8	2/10/1933	0	5/20/1933	13	8/27/1933	0.2	12/4/1933	0
6/28/1931	1.2	10/5/1931	0.1	1/12/1932	0	4/20/1932	81	7/28/1932	0	11/4/1932	0.8	2/11/1933	0	5/21/1933	13	8/28/1933	0.2	12/5/1933	0
6/29/1931	1.1	10/6/1931	0.1	1/13/1932	0	4/21/1932	74	7/29/1932	0	11/5/1932	0.8	2/12/1933	0	5/22/1933	13	8/29/1933	0.2	12/6/1933	0
6/30/1931	1.1	10/7/1931	0.1	1/14/1932	0	4/22/1932	67	7/30/1932	0	11/6/1932	0.8	2/13/1933	0	5/23/1933	21	8/30/1933	0.2	12/7/1933	0
7/1/1931	4.4	10/8/1931	0.1	1/15/1932	0	4/23/1932	67	7/31/1932	0	11/7/1932	0.8	2/14/1933	0	5/24/1933	18	8/31/1933	0.2	12/8/1933	0
7/2/1931	1.7	10/9/1931	0.1	1/16/1932	0	4/24/1932	67	8/1/1932	0	11/8/1932	0.8	2/15/1933	0	5/25/1933	21	9/1/1933	0	12/9/1933	0
7/3/1931	1.3	10/10/1931	0.1	1/17/1932	0	4/25/1932	56	8/2/1932	0	11/9/1932	0.8	2/16/1933	0	5/26/1933	22	9/2/1933	0	12/10/1933	0
7/4/1931	1.4	10/11/1931	0.1	1/18/1932	0	4/26/1932	50	8/3/1932	0	11/10/1932	0.8	2/17/1933	0	5/27/1933	22	9/3/1933	0	12/11/1933	0
7/5/1931	1.5	10/12/1931	0.1	1/19/1932	0	4/27/1932	39	8/4/1932	0	11/11/1932	0.8	2/18/1933	0	5/28/1933	30	9/4/1933	0	12/12/1933	0
7/6/1931	1.7	10/13/1931	0.1	1/20/1932	0	4/28/1932	35	8/5/1932	0	11/12/1932	0.8	2/19/1933	0	5/29/1933	36	9/5/1933	0	12/13/1933	0
7/7/1931	1.3	10/14/1931	0.1	1/21/1932	0	4/29/1932	46	8/6/1932	0	11/13/1932	0.8	2/20/1933	0	5/30/1933	32	9/6/1933	0	12/14/1933	0
7/8/1931	1.3	10/15/1931	0.1	1/22/1932	0	4/30/1932	35	8/7/1932	0	11/14/1932	0.8	2/21/1933	0	5/31/1933	27	9/7/1933	0	12/15/1933	0
7/9/1931	1.6	10/16/1931	0.1	1/23/1932	0	5/1/1932	33	8/8/1932	0	11/15/1932	0.8	2/22/1933	0	6/1/1933	25	9/8/1933	0	12/16/1933	0
7/10/1931	1	10/17/1931	0.1	1/24/1932	0	5/2/1932	33	8/9/1932	0	11/16/1932	0.8	2/23/1933	0	6/2/1933	19	9/9/1933	0	12/17/1933	0
7/11/1931	1.3	10/18/1931	0.1	1/25/1932	0	5/3/1932	29	8/10/1932	0	11/17/1932	0.8	2/24/1933	0	6/3/1933	17	9/10/1933	0	12/18/1933	0
7/12/1931	1.8	10/19/1931	0.1	1/26/1932	0	5/4/1932	34	8/11/1932	0	11/18/1932	0.8	2/25/1933	0	6/4/1933	17	9/11/1933	0	12/19/1933	0
7/13/1931	1.6	10/20/1931	0.1	1/27/1932	0	5/5/1932	39	8/12/1932	0	11/19/1932	0.8	2/26/1933	0	6/5/1933	19	9/12/1933	0	12/20/1933	0
7/14/1931	1.3	10/21/1931	0.1	1/28/1932	0	5/6/1932	29	8/13/1932	0	11/20/1932	0.8	2/27/1933	0	6/6/1933	17	9/13/1933	0	12/21/1933	0
7/15/1931	1	10/22/1931	0.1	1/29/1932	0	5/7/1932	33	8/14/1932	0	11/21/1932	0.8	2/28/1933	0	6/7/1933	19	9/14/1933	0	12/22/1933	0
7/16/1931	1.3	10/23/1931	0.1	1/30/1932	0	5/8/1932	34	8/15/1932	0	11/22/1932	0.8	3/1/1933	40	6/8/1933	40	9/15/1933	0	12/23/1933	0
7/17/1931	1	10/24/1931	0.1	1/31/1932	0	5/9/1932	33	8/16/1932	0	11/23/1932	0.8	3/2/1933	40	6/9/1933	29	9/16/1933	0	12/24/1933	0
7/18/1931	0.7	10/25/1931	0.1	2/1/1932	2	5/10/1932	35	8/17/1932	0	11/24/1932	0.8	3/3/1933	40	6/10/1933	34	9/17/1933	0	12/25/1933	0
7/19/1931	0.7	10/26/1931	0.1	2/2/1932	2	5/11/1932	42	8/18/1932	0	11/25/1932	0.8	3/4/1933	40	6/11/1933	27	9/18/1933	0	12/26/1933	0
7/20/1931	0.6	10/27/1931	0.1	2/3/1932	2	5/12/1932	32	8/19/1932	0	11/26/1932	0.8	3/5/1933	40	6/12/1933	24	9/19/1933	0	12/27/1933	0
7/21/1931	0.4	10/28/1931	0.1	2/4/1932	2	5/13/1932	27	8/20/1932	0	11/27/1932	0.8	3/6/1933	40	6/13/1933	20	9/20/1933	0	12/28/1933	0
7/22/1931	0.3	10/29/1931	0.1	2/5/1932	2	5/14/1932	28	8/21/1932	0	11/28/1932	0.8	3/7/1933	40	6/14/1933	13	9/21/1933	0	12/29/1933	0
7/23/1931	0.3	10/30/1931	0.1	2/6/1932	2	5/15/1932	25	8/22/1932	0	11/29/1932	0.8	3/8/1933	40	6/15/1933	9.6	9/22/1933	0	12/30/1933	0
7/24/1931	0.2	10/31/1931	0.1	2/7/1932	2	5/16/1932	21	8/23/1932	0	11/30/1932	0.8	3/9/1933	40	6/16/1933	7.5	9/23/1933	0	12/31/1933	0
7/25/1931	0.2	11/1/1931	0.4	2/8/1932	2	5/17/1932	17	8/24/1932	0	12/1/1932	0	3/10/1933	40	6/17/1933	5.6	9/24/1933	0	1/1/1934	0
7/26/1931	0.1	11/2/1931	0.4	2/9/1932	2	5/18/1932	15	8/25/1932	0	12/2/1932	0	3/11/1933	40	6/18/1933	4.4	9/25/1933	0	1/2/1934	0
7/27/1931	0.1	11/3/1931	0.4	2/10/1932	2	5/19/1932	17	8/26/1932	0	12/3/1932	0	3/12/1933	40	6/19/1933	3	9/26/1933	0	1/3/1934	0
7/28/1931	0.1	11/4/1931	0.4	2/11/1932	2	5/20/1932	17	8/27/1932	0	12/4/1932	0	3/13/1933	40	6/20/1933	2	9/27/1933	0	1/4/1934	0
7/29/1931	0.1	11/5/1931	0.4	2/12/1932	2	5/21/1932	14	8/28/1932	0	12/5/1932	0	3/14/1933	40	6/21/1933	2.2	9/28/1933	0	1/5/1934	0
7/30/1931	0.1	11/6/1931	0.4	2/13/1932	2	5/22/1932	12	8/29/1932	0	12/6/1932	0	3/15/1933	40	6/22/1933	2.3	9/29/1933	0	1/6/1934	0
7/31/1931	0.1	11/7/1931	0.4	2/14/1932	2	5/23/1932	12	8/30/1932	0	12/7/1932	0	3/16/1933	40	6/23/1933	2.5	9/30/1933	0	1/7/1934	0
8/1/1931	0.2	11/8/1931	0.4	2/15/1932	2	5/24/1932	7.3	8/31/1932	0	12/8/1932	0	3/17/1933	40	6/24/1933	1.7	10/1/1933	0	1/8/1934	0
8/2/1931	0.5	11/9/1931	0.4	2/16/1932	2	5/25/1932	8.1	9/1/1932	0	12/9/1932	0	3/18/1933	40	6/25/1933	1.4	10/2/1933	0	1/9/1934	0
8/3/1931	0.6	11/10/1931	0.4	2/17/1932	2	5/26/1932	8.1	9/2/1932	0	12/10/1932	0	3/19/1933	40	6/26/1933	1.3	10/3/1933	0	1/10/1934	0
8/4/1931	0.5	11/11/1931	0.4	2/18/1932	2	5/27/1932	7.3	9/3/1932	0	12/11/1932	0	3/20/1933	40	6/27/1933	1.3	10/4/1933	0	1/11/1934	0
8/5/1931	0.3	11/12/1931	0.4	2/19/1932	2	5/28/1932	8.1	9/4/1932	0	12/12/1932	0	3/21/1933	40	6/28/1933	0.9	10/5/1933	0	1/12/1934	0
8/6/1931	0.2	11/13/1931	0.4	2/20/1932	2	5/29/1932	8.1	9/5/1932	0	12/13/1932	0	3/22/1933	40	6/29/1933	0.8	10/6/1933	0	1/13/1934	0
8/7/1931	0.2	11/14/1931	0.4	2/21/1932	2	5/30/1932	8.1	9/6/1932	0	12/14/1932	0	3/23/1933	40	6/30/1933	0.7	10/7/1933	0	1/14/1934	0

Date	discharge cfs
1/15/1934	0
1/16/1934	0
1/17/1934	0
1/18/1934	0
1/19/1934	0
1/20/1934	0
1/21/1934	0
1/22/1934	0
1/23/1934	0
1/24/1934	0
1/25/1934	0
1/26/1934	0
1/27/1934	0
1/28/1934	0
1/29/1934	0
1/30/1934	0
1/31/1934	0
2/1/1934	0.05
2/2/1934	0.05
2/3/1934	0.05
2/4/1934	0.05
2/5/1934	0.05
2/6/1934	0.05
2/7/1934	0.05
2/8/1934	0.05
2/9/1934	0.05
2/10/1934	0.05
2/11/1934	0.05
2/12/1934	0.05
2/13/1934	0.05
2/14/1934	0.05
2/15/1934	0.05
2/16/1934	0.05
2/17/1934	0.05
2/18/1934	0.05
2/19/1934	0.05
2/20/1934	0.05
2/21/1934	0.05
2/22/1934	0.05
2/23/1934	0.05
2/24/1934	0.05
2/25/1934	0.05
2/26/1934	0.05
2/27/1934	0.05
2/28/1934	0.05
3/1/1934	15
3/2/1934	15
3/3/1934	15
3/4/1934	15
3/5/1934	15
3/6/1934	15
3/7/1934	15
3/8/1934	15
3/9/1934	15
3/10/1934	15
3/11/1934	15
3/12/1934	15
3/13/1934	15
3/14/1934	15
3/15/1934	15
3/16/1934	15
3/17/1934	15
3/18/1934	15
3/19/1934	15
3/20/1934	15
3/21/1934	15
3/22/1934	15
3/23/1934	15
3/24/1934	15
3/25/1934	15
3/26/1934	15
3/27/1934	15
3/28/1934	15
3/29/1934	15
3/30/1934	15
3/31/1934	15
4/1/1934	24
4/2/1934	33
4/3/1934	34
4/4/1934	65
4/5/1934	112
4/6/1934	126
4/7/1934	185
4/8/1934	273
4/9/1934	393
4/10/1934	251
4/11/1934	174
4/12/1934	126
4/13/1934	90
4/14/1934	58
4/15/1934	62
4/16/1934	48
4/17/1934	40
4/18/1934	43
4/19/1934	34
4/20/1934	29
4/21/1934	26
4/22/1934	22
4/23/1934	20

Date	dis. cfs
4/24/1934	14
4/25/1934	14
4/26/1934	12
4/27/1934	9
4/28/1934	8.4
4/29/1934	7.8
4/30/1934	7.1
5/1/1934	6.5
5/2/1934	6.5
5/3/1934	6.5
5/4/1934	6.5
5/5/1934	5.5
5/6/1934	5.5
5/7/1934	4.2
5/8/1934	3.2
5/9/1934	2.3
5/10/1934	1.7
5/11/1934	0.8
5/12/1934	0.7
5/13/1934	1
5/14/1934	1.1
5/15/1934	1.2
5/16/1934	1.2
5/17/1934	1
5/18/1934	1
5/19/1934	1.6
5/20/1934	1.4
5/21/1934	1.3
5/22/1934	1
5/23/1934	0.9
5/24/1934	0.8
5/25/1934	0.8
5/26/1934	0.6
5/27/1934	0.6
5/28/1934	0.5
5/29/1934	0.4
5/30/1934	0.3
5/31/1934	0.4
6/1/1934	0.3
6/2/1934	0.2
6/3/1934	0.1
6/4/1934	0.1
6/5/1934	0.5
6/6/1934	0.2
6/7/1934	0.6
6/8/1934	0.6
6/9/1934	0.7
6/10/1934	0.6
6/11/1934	0.8
6/12/1934	1
6/13/1934	1
6/14/1934	0.9
6/15/1934	0.7
6/16/1934	0.7
6/17/1934	0.7
6/18/1934	0.4
6/19/1934	0.2
6/20/1934	0.2
6/21/1934	0.1
6/22/1934	0.1
6/23/1934	0.1
6/24/1934	0
6/25/1934	0.8
6/26/1934	0.3
6/27/1934	0.3
6/28/1934	0.1
6/29/1934	0.1
6/30/1934	0
7/1/1934	0
7/2/1934	0
7/3/1934	0
7/4/1934	0
7/5/1934	0
7/6/1934	0.1
7/7/1934	0.1
7/8/1934	0
7/9/1934	0
7/10/1934	0
7/11/1934	0
7/12/1934	0
7/13/1934	0
7/14/1934	0
7/15/1934	0
7/16/1934	0
7/17/1934	0
7/18/1934	0
7/19/1934	0.1
7/20/1934	0.1
7/21/1934	0
7/22/1934	0
7/23/1934	0
7/24/1934	0
7/25/1934	0
7/26/1934	0
7/27/1934	0
7/28/1934	0
7/29/1934	0
7/30/1934	0
7/31/1934	0

Date	dis. cfs
8/1/1934	0
8/2/1934	0
8/3/1934	0
8/4/1934	0
8/5/1934	0
8/6/1934	0
8/7/1934	0
8/8/1934	0
8/9/1934	0
8/10/1934	0
8/11/1934	0
8/12/1934	0
8/13/1934	0
8/14/1934	0
8/15/1934	0
8/16/1934	0
8/17/1934	0
8/18/1934	0
8/19/1934	0
8/20/1934	0
8/21/1934	0
8/22/1934	0
8/23/1934	0
8/24/1934	0
8/25/1934	0
8/26/1934	0
8/27/1934	0
8/28/1934	0
8/29/1934	0
8/30/1934	0
8/31/1934	0
9/1/1934	0
9/2/1934	0
9/3/1934	0
9/4/1934	0
9/5/1934	0
9/6/1934	0
9/7/1934	0
9/8/1934	0
9/9/1934	0
9/10/1934	0
9/11/1934	0
9/12/1934	0
9/13/1934	0
9/14/1934	0
9/15/1934	0
9/16/1934	0
9/17/1934	0
9/18/1934	0
9/19/1934	0
9/20/1934	0
9/21/1934	0
9/22/1934	0
9/23/1934	0
9/24/1934	0
9/25/1934	0
9/26/1934	0
9/27/1934	0.8
9/28/1934	0.6
9/29/1934	0.5
9/30/1934	0.5
10/1/1934	0
10/2/1934	0
10/3/1934	0
10/4/1934	0
10/5/1934	0
10/6/1934	0
10/7/1934	0
10/8/1934	0
10/9/1934	0
10/10/1934	0
10/11/1934	0
10/12/1934	0
10/13/1934	0
10/14/1934	0
10/15/1934	0
10/16/1934	0
10/17/1934	0
10/18/1934	0
10/19/1934	0
10/20/1934	0
10/21/1934	0
10/22/1934	0
10/23/1934	0
10/24/1934	0
10/25/1934	0
10/26/1934	0
10/27/1934	0
10/28/1934	0
10/29/1934	0
10/30/1934	0
10/31/1934	0
11/1/1934	0
11/2/1934	0
11/3/1934	0
11/4/1934	0
11/5/1934	0
11/6/1934	0
11/7/1934	0

Date	dis. cfs
11/8/1934	0
11/9/1934	0
11/10/1934	0
11/11/1934	0
11/12/1934	0
11/13/1934	0
11/14/1934	0
11/15/1934	0
11/16/1934	0
11/17/1934	0
11/18/1934	0
11/19/1934	0
11/20/1934	0
11/21/1934	0
11/22/1934	0
11/23/1934	0
11/24/1934	0
11/25/1934	0
11/26/1934	0
11/27/1934	0
11/28/1934	0
11/29/1934	0
11/30/1934	0
12/1/1934	0
12/2/1934	0
12/3/1934	0
12/4/1934	0
12/5/1934	0
12/6/1934	0
12/7/1934	0
12/8/1934	0
12/9/1934	0
12/10/1934	0
12/11/1934	0
12/12/1934	0
12/13/1934	0
12/14/1934	0
12/15/1934	0
12/16/1934	0
12/17/1934	0
12/18/1934	0
12/19/1934	0
12/20/1934	0
12/21/1934	0
12/22/1934	0
12/23/1934	0
12/24/1934	0
12/25/1934	0
12/26/1934	0
12/27/1934	0
12/28/1934	0
12/29/1934	0
12/30/1934	0
12/31/1934	0
1/1/1935	0
1/2/1935	0
1/3/1935	0
1/4/1935	0
1/5/1935	0
1/6/1935	0
1/7/1935	0
1/8/1935	0
1/9/1935	0
1/10/1935	0
1/11/1935	0
1/12/1935	0
1/13/1935	0
1/14/1935	0
1/15/1935	0
1/16/1935	0
1/17/1935	0
1/18/1935	0
1/19/1935	0
1/20/1935	0
1/21/1935	0
1/22/1935	0
1/23/1935	0
1/24/1935	0
1/25/1935	0
1/26/1935	0
1/27/1935	0
1/28/1935	0
1/29/1935	0
1/30/1935	0
1/31/1935	0
2/1/1935	0
2/2/1935	0
2/3/1935	0
2/4/1935	0
2/5/1935	0
2/6/1935	0
2/7/1935	0
2/8/1935	0
2/9/1935	0
2/10/1935	0
2/11/1935	0
2/12/1935	0
2/13/1935	0
2/14/1935	0

Date	dis. cfs
2/15/1935	0
2/16/1935	0
2/17/1935	0
2/18/1935	0
2/19/1935	0
2/20/1935	0
2/21/1935	0
2/22/1935	0
2/23/1935	0
2/24/1935	0
2/25/1935	0
2/26/1935	0
2/27/1935	0
2/28/1935	0
3/1/1935	0
3/2/1935	0
3/3/1935	0
3/4/1935	0
3/5/1935	0
3/6/1935	0
3/7/1935	0
3/8/1935	0
3/9/1935	0
3/10/1935	0
3/11/1935	0
3/12/1935	0
3/13/1935	0
3/14/1935	2
3/15/1935	5
3/16/1935	10
3/17/1935	20
3/18/1935	50
3/19/1935	51
3/20/1935	40
3/21/1935	90
3/22/1935	43
3/23/1935	164
3/24/1935	51
3/25/1935	62
3/26/1935	117
3/27/1935	345
3/28/1935	443
3/29/1935	229
3/30/1935	240
3/31/1935	174
4/1/1935	135
4/2/1935	99
4/3/1935	73
4/4/1935	54
4/5/1935	51
4/6/1935	43
4/7/1935	43
4/8/1935	37
4/9/1935	54
4/10/1935	65
4/11/1935	104
4/12/1935	144
4/13/1935	196
4/14/1935	154
4/15/1935	126
4/16/1935	108
4/17/1935	81
4/18/1935	65
4/19/1935	51
4/20/1935	40
4/21/1935	34
4/22/1935	29
4/23/1935	24
4/24/1935	22
4/25/1935	27
4/26/1935	27
4/27/1935	27
4/28/1935	22
4/29/1935	22
4/30/1935	20
5/1/1935	20
5/2/1935	16
5/3/1935	12
5/4/1935	9.4
5/5/1935	8.7
5/6/1935	7.1
5/7/1935	6.5
5/8/1935	7.1
5/9/1935	12
5/10/1935	5.5
5/11/1935	3.6
5/12/1935	5.2
5/13/1935	7.4
5/14/1935	6.3
5/15/1935	6
5/16/1935	5.2
5/17/1935	5.8
5/18/1935	5.2
5/19/1935	4.2
5/20/1935	3.8
5/21/1935	3.8
5/22/1935	3.2
5/23/1935	2.5
5/24/1935	2.3

Date	dis. cfs
5/25/1935	2.3
5/26/1935	3.4
5/27/1935	4.2
5/28/1935	3.6
5/29/1935	3.6
5/30/1935	1.2
5/31/1935	1.2
6/1/1935	1.4
6/2/1935	3.2
6/3/1935	2.7
6/4/1935	1.6
6/5/1935	1.2
6/6/1935	1.3
6/7/1935	1
6/8/1935	1
6/9/1935	0.9
6/10/1935	0.7
6/11/1935	0.4
6/12/1935	1
6/13/1935	2.7
6/14/1935	2.7
6/15/1935	2.9
6/16/1935	3.6
6/17/1935	5.8
6/18/1935	5
6/19/1935	4
6/20/1935	2.5
6/21/1935	2
6/22/1935	4.7
6/23/1935	5
6/24/1935	4
6/25/1935	5
6/26/1935	18
6/27/1935	14
6/28/1935	8.4
6/29/1935	5.5
6/30/1935	5
7/1/1935	2
7/2/1935	2
7/3/1935	2
7/4/1935	2
7/5/1935	2
7/6/1935	2
7/7/1935	2
7/8/1935	2
7/9/1935	2
7/10/1935	2
7/11/1935	2
7/12/1935	2
7/13/1935	2
7/14/1935	2
7/15/1935	2
7/16/1935	2
7/17/1935	2
7/18/1935	2
7/19/1935	2
7/20/1935	2
7/21/1935	2
7/22/1935	2
7/23/1935	2
7/24/1935	2
7/25/1935	2
7/26/1935	2
7/27/1935	2
7/28/1935	2
7/29/1935	2
7/30/1935	2
7/31/1935	2
8/1/1935	0.5
8/2/1935	0.5
8/3/1935	0.5
8/4/1935	0.5
8/5/1935	0.5
8/6/1935	0.5
8/7/1935	0.5
8/8/1935	0.5
8/9/1935	0.5
8/10/1935	0.5
8/11/1935	0.5
8/12/1935	0.5
8/13/1935	0.5
8/14/1935	0.5
8/15/1935	0.5
8/16/1935	0.5
8/17/1935	0.5
8/18/1935	0.5
8/19/1935	0.5
8/20/1935	0.5
8/21/1935	0.5
8/22/1935	0.5
8/23/1935	0.5
8/24/1935	0.5
8/25/1935	0.5
8/26/1935	0.5
8/27/1935	0.5
8/28/1935	0.5
8/29/1935	0.5
8/30/1935	0.5
8/31/1935	0.5

Date	dis. cfs
9/1/1935	0
9/2/1935	0
9/3/1935	0
9/4/1935	0
9/5/1935	0
9/6/1935	0
9/7/1935	0
9/8/1935	0
9/9/1935	0
9/10/1935	0
9/11/1935	0
9/12/1935	0
9/13/1935	0
9/14/1935	0
9/15/1935	0
9/16/1935	0
9/17/1935	0
9/18/1935	0
9/19/1935	0
9/20/1935	0
9/21/1935	0
9/22/1935	0
9/23/1935	0
9/24/1935	0
9/25/1935	0
9/26/1935	0
9/27/1935	0
9/28/1935	0
9/29/1935	0
9/30/1935	0
10/1/1935	0
10/2/1935	0
10/3/1935	0
10/4/1935	0
10/5/1935	0
10/6/1935	0
10/7/1935	0
10/8/1935	0
10/9/1935	0
10/10/1935	0
10/11/1935	0
10/12/1935	0
10/13/1935	0
10/14/1935	0
10/15/1935	0
10/16/1935	0
10/17/1935	0
10/18/1935	0
10/19/1935	0
10/20/1935	0
10/21/1935	0
10/22/1935	0
10/23/1935	0
10/24/1935	0
10/25/1935	0
10/26/1935	0
10/27/1935	0
10/28/1935	0
10/29/1935	0
10/30/1935	0
10/31/1935	0
11/1/1935	0
11/2/1935	0
11/3/1935	0
11/4/1935	0
11/5/1935	0
11/6/1935	0
11/7/1935	0
11/8/1935	0
11/9/1935	0
11/10/1935	0
11/11/1935	0
11/12/1935	0
11/13/1935	0
11/14/1935	0
11/15/1935	0
11/16/1935	0
11/17/1935	0
11/18/1935	0
11/19/1935	0
11/20/1935	0
11/21/1935	0
11/22/1935	0
11/23/1935	0
11/24/1935	0
11/25/1935	0
11/26/1935	0
11/27/1935	0
11/28/1935	0
11/29/1935	0
11/30/1935	0
12/1/1935	0
12/2/1935	0
12/3/1935	0
12/4/1935	0
12/5/1935	0
12/6/1935	0
12/7/1935	0
12/8/1935	0

Date	dis. cfs
12/9/1935	0
12/10/1935	0
12/11/1935	0
12/12/1935	0
12/13/1935	0
12/14/1935	0
12/15/1935	0
12/16/1935	0
12/17/1935	0
12/18/1935	0
12/19/1935	0
12/20/1935	0
12/21/1935	0
12/22/1935	0
12/23/1935	0
12/24/1935	0
12/25/1935	0
12/26/1935	0
12/27/1935	0
12/28/1935	0
12/29/1935	0
12/30/1935	0
12/31/1935	0
1/1/1936	0
1/2/1936	0
1/3/1936	0
1/4/1936	0
1/5/1936	0
1/6/1936	0
1/7/1936	0
1/8/1936	0
1/9/1936	0
1/10/1936	0
1/11/1936	0
1/12/1936	0
1/13/1936	0
1/14/1936	0
1/15/1936	0
1/16/1936	0
1/17/1936	0
1/18/1936	0
1/19/1936	0
1/20/1936	0
1/21/1936	0
1/22/1936	0
1/23/1936	0
1/24/1936	0
1/25/1936	0
1/26/1936	0
1/27/1936	0
1/28/1936	0
1/29/1936	0
1/30/1936	0
1/31/1936	0
2/1/1936	0
2/2/1936	0
2/3/1936	0
2/4/1936	0
2/5/1936	0
2/6/1936	0
2/7/1936	0
2/8/1936	0
2/9/1936	0
2/10/1936	0
2/11/1936	0
2/12/1936	0
2/13/1936	0
2/14/1936	0
2/15/1936	0
2/16/1936	0
2/17/1936	0
2/18/1936	0
2/19/1936	0
2/20/1936	0
2/21/1936	0
2/22/1936	0
2/23/1936	0
2/24/1936	0
2/25/1936	0
2/26/1936	0
2/27/1936	0
2/28/1936	0
2/29/1936	0
3/1/1936	0
3/2/1936	0
3/3/1936	0
3/4/1936	0
3/5/1936	0
3/6/1936	0
3/7/1936	0
3/8/1936	0
3/9/1936	0
3/10/1936	0
3/11/1936	0
3/12/1936	0
3/13/1936	0
3/14/1936	0
3/15/1936	0
3/16/1936	0

Date	dis. cfs
3/17/1936	0
3/18/1936	0
3/19/1936	0
3/20/1936	0
3/21/1936	0
3/22/1936	0
3/23/1936	0
3/24/1936	0
3/25/1936	0
3/26/1936	0
3/27/1936	0
3/28/1936	0
3/29/1936	0
3/30/1936	0
3/31/1936	0
4/1/1936	0
4/2/1936	0
4/3/1936	0
4/4/1936	0
4/5/1936	0
4/6/1936	0
4/7/1936	0
4/8/1936	0
4/9/1936	0
4/10/1936	0
4/11/1936	0
4/12/1936	40
4/13/1936	513
4/14/1936	1120
4/15/1936	1140
4/16/1936	1060
4/17/1936	860
4/18/1936	547
4/19/1936	336
4/20/1936	262
4/21/1936	207
4/22/1936	155
4/23/1936	117
4/24/1936	99
4/25/1936	82
4/26/1936	68
4/27/1936	56
4/28/1936	59
4/29/1936	48
4/30/1936	42
5/1/1936	38
5/2/1936	35
5/3/1936	32
5/4/1936	28
5/5/1936	23
5/6/1936	25
5/7/1936	20
5/8/1936	19
5/9/1936	14
5/10/1936	11
5/11/1936	11
5/12/1936	14
5/13/1936	9.5
5/14/1936	9.5
5/15/1936	9.5
5/16/1936	8.8
5/17/1936	8
5/18/1936	8
5/19/1936	7.2
5/20/1936	5.8
5/21/1936	5.8
5/22/1936	5
5/23/1936	5
5/24/1936	3.5
5/25/1936	3.5
5/26/1936	3.5
5/27/1936	2.4
5/28/1936	2.4
5/29/1936	2.4
5/30/1936	2.1
5/31/1936	1.4
6/1/1936	1
6/2/1936	1
6/3/1936	0.7
6/4/1936	0.7
6/5/1936	1.4
6/6/1936	16
6/7/1936	36
6/8/1936	42
6/9/1936	148
6/10/1936	54
6/11/1936	32
6/12/1936	16
6/13/1936	9.5
6/14/1936	8
6/15/1936	6.5
6/16/1936	5
6/17/1936	3.2
6/18/1936	2.8
6/19/1936	1.4
6/20/1936	1.4
6/21/1936	0.7
6/22/1936	1
6/23/1936	0.4

Date	dis. cfs
6/24/1936	0
6/25/1936	0
6/26/1936	0
6/27/1936	0
6/28/1936	0
6/29/1936	0
6/30/1936	0.7
7/1/1936	0.7
7/2/1936	0
7/3/1936	0
7/4/1936	0
7/5/1936	0
7/6/1936	0
7/7/1936	0
7/8/1936	0
7/9/1936	0.4
7/10/1936	0
7/11/1936	0
7/12/1936	0
7/13/1936	0
7/14/1936	0
7/15/1936	0
7/16/1936	0
7/17/1936	0
7/18/1936	0
7/19/1936	0
7/20/1936	0
7/21/1936	0
7/22/1936	0
7/23/1936	0
7/24/1936	0
7/25/1936	0
7/26/1936	0
7/27/1936	0
7/28/1936	0
7/29/1936	0
7/30/1936	0
7/31/1936	0
8/1/1936	0
8/2/1936	0
8/3/1936	0
8/4/1936	0
8/5/1936	0
8/6/1936	0
8/7/1936	0
8/8/1936	0
8/9/1936	0
8/10/1936	0
8/11/1936	0
8/12/1936	0
8/13/1936	0
8/14/1936	0
8/15/1936	0
8/16/1936	0
8/17/1936	0
8/18/1936	0
8/19/1936	0
8/20/1936	0
8/21/1936	0
8/22/1936	0
8/23/1936	0
8/24/1936	0
8/25/1936	0
8/26/1936	0
8/27/1936	0
8/28/1936	0
8/29/1936	0
8/30/1936	0
8/31/1936	0
9/1/1936	0
9/2/1936	0
9/3/1936	0
9/4/1936	0
9/5/1936	0
9/6/1936	0
9/7/1936	0
9/8/1936	0
9/9/1936	0
9/10/1936	0
9/11/1936	0
9/12/1936	0
9/13/1936	0
9/14/1936	0
9/15/1936	0
9/16/1936	0
9/17/1936	0
9/18/1936	0
9/19/1936	0
9/20/1936	0
9/21/1936	0
9/22/1936	0
9/23/1936	0
9/24/1936	0
9/25/1936	0
9/26/1936	0
9/27/1936	0
9/28/1936	0
9/29/1936	0
9/30/1936	0

....

Date	discharge cfs	Date	dis. cfs	Date	dis. cfs	Date	dis. cfs	Date	dis. cfs	Date	dis. cfs	Date	dis. cfs	Date	dis. cfs	Date	dis. cfs	Date	dis. cfs
7/31/2018	7.67	11/7/2018	0.94	2/14/2019	0.74	5/24/2019	46.4	8/31/2019	2.31	12/8/2019	25.8	3/16/2020	12.4	6/23/2020	26.2	9/30/2020	4.48	1/7/2021	1.18
8/1/2018	6.78	11/8/2018	0.88	2/15/2019	0.72	5/25/2019	54.2	9/1/2019	2.33	12/9/2019	25.7	3/17/2020	12.3	6/24/2020	23.8	10/1/2020	4.21	1/8/2021	1.18
8/2/2018	6.88	11/9/2018	0.79	2/16/2019	0.71	5/26/2019	57.5	9/2/2019	2.41	12/10/2019	25.3	3/18/2020	12.3	6/25/2020	21.6	10/2/2020	4.3	1/9/2021	1.18
8/3/2018	6.61	11/10/2018	0.69	2/17/2019	0.71	5/27/2019	58.8	9/3/2019	2.47	12/11/2019	24.4	3/19/2020	12.4	6/26/2020	20.9	10/3/2020	4.33	1/10/2021	1.18
8/4/2018	7.2	11/11/2018	0.62	2/18/2019	0.7	5/28/2019	62.6	9/4/2019	2.5	12/12/2019	18.3	3/20/2020	12.5	6/27/2020	25.5	10/4/2020	4.35	1/11/2021	1.18
8/5/2018	7.08	11/12/2018	0.56	2/19/2019	0.7	5/29/2019	61.6	9/5/2019	2.56	12/13/2019	13.1	3/21/2020	12.7	6/28/2020	42.3	10/5/2020	4.27	1/12/2021	1.18
8/6/2018	6.47	11/13/2018	0.53	2/20/2019	0.69	5/30/2019	56.4	9/6/2019	2.44	12/14/2019	11.6	3/22/2020	13.1	6/29/2020	36.8	10/6/2020	4.16	1/13/2021	1.18
8/7/2018	6.44	11/14/2018	0.54	2/21/2019	0.69	5/31/2019	51.5	9/7/2019	2.42	12/15/2019	11.4	3/23/2020	14.2	6/30/2020	27.6	10/7/2020	4.05	1/14/2021	1.18
8/8/2018	6.34	11/15/2018	0.94	2/22/2019	0.68	6/1/2019	43.7	9/8/2019	2.42	12/16/2019	11.3	3/24/2020	15.7	7/1/2020	54.6	10/8/2020	3.93	1/15/2021	1.18
8/9/2018	6.04	11/16/2018	1.31	2/23/2019	0.67	6/2/2019	36.5	9/9/2019	2.71	12/17/2019	11	3/25/2020	15.8	7/2/2020	241	10/9/2020	4.05	1/16/2021	1.18
8/10/2018	5.5	11/17/2018	1.34	2/24/2019	0.67	6/3/2019	30.2	9/10/2019	3.2	12/18/2019	10.4	3/26/2020	16.7	7/3/2020	241	10/10/2020	4	1/17/2021	1.18
8/11/2018	4.73	11/18/2018	1.35	2/25/2019	0.66	6/4/2019	34.8	9/11/2019	3.09	12/19/2019	10.1	3/27/2020	22.3	7/4/2020	211	10/11/2020	4.1	1/18/2021	1.18
8/12/2018	4.47	11/19/2018	1.34	2/26/2019	0.65	6/5/2019	36.2	9/12/2019	2.75	12/20/2019	9.86	3/28/2020	60.1	7/5/2020	170	10/12/2020	5.38	1/19/2021	1.18
8/13/2018	4.16	11/20/2018	1.34	2/27/2019	0.65	6/6/2019	29.5	9/13/2019	2.85	12/21/2019	9.62	3/29/2020	175	7/6/2020	135	10/13/2020	5.18	1/20/2021	1.18
8/14/2018	4.32	11/21/2018	1.34	2/28/2019	0.64	6/7/2019	22.9	9/14/2019	2.76	12/22/2019	9.33	3/30/2020	332	7/7/2020	102	10/14/2020	4.15	1/21/2021	1.18
8/15/2018	4.4	11/22/2018	1.35	3/1/2019	0.63	6/8/2019	77.1	9/15/2019	2.69	12/23/2019	9.23	3/31/2020	713	7/8/2020	85.1	10/15/2020	3.7	1/22/2021	1.18
8/16/2018	4.42	11/23/2018	1.35	3/2/2019	0.63	6/9/2019	172	9/16/2019	2.59	12/24/2019	9.06	4/1/2020	1110	7/9/2020	78.8	10/16/2020	3.82	1/23/2021	1.18
8/17/2018	4.39	11/24/2018	1.34	3/3/2019	0.62	6/10/2019	141	9/17/2019	2.56	12/25/2019	8.96	4/2/2020	1150	7/10/2020	66.6	10/17/2020	3.85	1/24/2021	1.17
8/18/2018	4.38	11/25/2018	1.27	3/4/2019	0.62	6/11/2019	97.8	9/18/2019	2.55	12/26/2019	8.9	4/3/2020	968	7/11/2020	48.4	10/18/2020	3.81	1/25/2021	1.16
8/19/2018	4.63	11/26/2018	1.17	3/5/2019	0.61	6/12/2019	76	9/19/2019	2.59	12/27/2019	8.9	4/4/2020	766	7/12/2020	43	10/19/2020	3.82	1/26/2021	1.16
8/20/2018	4.23	11/27/2018	1.11	3/6/2019	0.6	6/13/2019	62.6	9/20/2019	2.83	12/28/2019	8.89	4/5/2020	734	7/13/2020	38.1	10/20/2020	3.85	1/27/2021	1.16
8/21/2018	4.06	11/28/2018	1.09	3/7/2019	0.6	6/14/2019	32.7	9/21/2019	5.74	12/29/2019	8.89	4/6/2020	670	7/14/2020	34.1	10/21/2020	3.91	1/28/2021	1.16
8/22/2018	4.06	11/29/2018	1.09	3/8/2019	0.59	6/15/2019	20.1	9/22/2019	3.82	12/30/2019	8.89	4/7/2020	774	7/15/2020	32.4	10/22/2020	3.81	1/29/2021	1.16
8/23/2018	3.99	11/30/2018	1.09	3/9/2019	0.59	6/16/2019	15.3	9/23/2019	2.74	12/31/2019	8.9	4/8/2020	1280	7/16/2020	25.9	10/23/2020	3.64	1/30/2021	1.16
8/24/2018	3.81	12/1/2018	1.1	3/10/2019	0.59	6/17/2019	14.5	9/24/2019	2.62	1/1/2020	8.9	4/9/2020	1110	7/17/2020	24.1	10/24/2020	3.38	1/31/2021	1.16
8/25/2018	3.77	12/2/2018	1.1	3/11/2019	0.59	6/18/2019	13.8	9/25/2019	1.99	1/2/2020	8.9	4/10/2020	990	7/18/2020	25.8	10/25/2020	3.33	2/1/2021	1.15
8/26/2018	3.8	12/3/2018	1.1	3/12/2019	0.59	6/19/2019	12.9	9/26/2019	1.75	1/3/2020	8.9	4/11/2020	881	7/19/2020	23.6	10/26/2020	3.29	2/2/2021	1.15
8/27/2018	3.87	12/4/2018	1.1	3/13/2019	0.59	6/20/2019	12.1	9/27/2019	2.56	1/4/2020	8.89	4/12/2020	716	7/20/2020	24.6	10/27/2020	3.26	2/3/2021	1.14
8/28/2018	3.96	12/5/2018	1.1	3/14/2019	0.58	6/21/2019	12	9/28/2019	2.79	1/5/2020	8.88	4/13/2020	561	7/21/2020	21	10/28/2020	3.2	2/4/2021	1.13
8/29/2018	3.86	12/6/2018	1.08	3/15/2019	0.58	6/22/2019	13.5	9/29/2019	2.93	1/6/2020	8.85	4/14/2020	448	7/22/2020	18.8	10/29/2020	3.13	2/5/2021	1.12
8/30/2018	3.63	12/7/2018	1.07	3/16/2019	0.57	6/23/2019	16.3	9/30/2019	4.05	1/7/2020	8.76	4/15/2020	373	7/23/2020	20.4	10/30/2020	3.05	2/6/2021	1.11
8/31/2018	3.42	12/8/2018	1.04	3/17/2019	0.56	6/24/2019	14.9	10/1/2019	3.93	1/8/2020	8.63	4/16/2020	314	7/24/2020	19.1	10/31/2020	2.98	2/7/2021	1.1
9/1/2018	3.31	12/9/2018	0.97	3/18/2019	0.57	6/25/2019	14.9	10/2/2019	3.8	1/9/2020	8.55	4/17/2020	282	7/25/2020	31	11/1/2020	2.93	2/8/2021	1.09
9/2/2018	3.21	12/10/2018	0.89	3/19/2019	0.61	6/26/2019	16	10/3/2019	5.01	1/10/2020	8.48	4/18/2020	264	7/26/2020	24.3	11/2/2020	2.92	2/9/2021	1.09
9/3/2018	3	12/11/2018	0.83	3/20/2019	1.91	6/27/2019	13.1	10/4/2019	4.06	1/11/2020	8.4	4/19/2020	253	7/27/2020	24.1	11/3/2020	2.92	2/10/2021	1.09
9/4/2018	2.89	12/12/2018	0.76	3/21/2019	10.3	6/28/2019	10.8	10/5/2019	4.45	1/12/2020	8.33	4/20/2020	263	7/28/2020	22	11/4/2020	2.92	2/11/2021	1.08
9/5/2018	2.73	12/13/2018	0.71	3/22/2019	16.7	6/29/2019	9.55	10/6/2019	4.57	1/13/2020	8.27	4/21/2020	222	7/29/2020	19	11/5/2020	2.92	2/12/2021	1.08
9/6/2018	2.73	12/14/2018	0.67	3/23/2019	36.5	6/30/2019	9.64	10/7/2019	3.63	1/14/2020	8.22	4/22/2020	197	7/30/2020	23.6	11/6/2020	2.92	2/13/2021	1.08
9/7/2018	2.79	12/15/2018	0.62	3/24/2019	84	7/1/2019	9.72	10/8/2019	3.55	1/15/2020	8.19	4/23/2020	175	7/31/2020	12.9	11/7/2020	2.91	2/14/2021	1.08
9/8/2018	2.76	12/16/2018	0.58	3/25/2019	64.1	7/2/2019	9.08	10/9/2019	3.87	1/16/2020	8.17	4/24/2020	162	8/1/2020	11.1	11/8/2020	2.9	2/15/2021	1.07
9/9/2018	2.87	12/17/2018	0.54	3/26/2019	34.5	7/3/2019	7.95	10/10/2019	3.98	1/17/2020	8.15	4/25/2020	163	8/2/2020	9.87	11/9/2020	2.8	2/16/2021	1.07
9/10/2018	3.07	12/18/2018	0.51	3/27/2019	20.4	7/4/2019	8.72	10/11/2019	7.75	1/18/2020	8.15	4/26/2020	165	8/3/2020	9.2	11/10/2020	2.6	2/17/2021	1.07
9/11/2018	3.09	12/19/2018	0.47	3/28/2019	17.6	7/5/2019	7.05	10/12/2019	14	1/19/2020	8.16	4/27/2020	160	8/4/2020	8.51	11/11/2020	2.47	2/18/2021	1.07
9/12/2018	3.02	12/20/2018	0.44	3/29/2019	23.8	7/6/2019	6.23	10/13/2019	18.8	1/20/2020	8.16	4/28/2020	157	8/5/2020	8.15	11/12/2020	2.36	2/19/2021	1.1
9/13/2018	2.78	12/21/2018	0.41	3/30/2019	32	7/7/2019	5.79	10/14/2019	11.5	1/21/2020	8.16	4/29/2020	157	8/6/2020	8.49	11/13/2020	2.26	2/20/2021	1.2
9/14/2018	2.7	12/22/2018	0.38	3/31/2019	28.6	7/8/2019	5.66	10/15/2019	23.5	1/22/2020	8.16	4/30/2020	160	8/7/2020	9.34	11/14/2020	2.21	2/21/2021	1.28
9/15/2018	2.78	12/23/2018	0.35	4/1/2019	81.2	7/9/2019	6.52	10/16/2019	40.3	1/23/2020	8.15	5/1/2020	168	8/8/2020	15.2	11/15/2020	2.17	2/22/2021	1.36
9/16/2018	2.76	12/24/2018	0.33	4/2/2019	291	7/10/2019	6.53	10/17/2019	71.8	1/24/2020	8.14	5/2/2020	165	8/9/2020	20.4	11/16/2020	2.13	2/23/2021	1.43
9/17/2018	2.6	12/25/2018	0.31	4/3/2019	579	7/11/2019	5.95	10/18/2019	99.3	1/25/2020	8.12	5/3/2020	171	8/10/2020	24	11/17/2020	2.09	2/24/2021	1.5
9/18/2018	2.57	12/26/2018	0.3	4/4/2019	737	7/12/2019	6.27	10/19/2019	187	1/26/2020	8.12	5/4/2020	162	8/11/2020	25.3	11/18/2020	2.03	2/25/2021	1.54
9/19/2018	2.47	12/27/2018	0.29	4/5/2019	614	7/13/2019	5.39	10/20/2019	362	1/27/2020	8.12	5/5/2020	161	8/12/2020	10.8	11/19/2020	2	2/26/2021	1.6
9/20/2018	2.67	12/28/2018	0.27	4/6/2019	507	7/14/2019	4.86	10/21/2019	437	1/28/2020	8.14	5/6/2020	116	8/13/2020	9.36	11/20/2020	1.99	2/27/2021	1.63
9/21/2018	2.6	12/29/2018	0.26	4/7/2019	568	7/15/2019	4.8	10/22/2019	520	1/29/2020	8.15	5/7/2020	183	8/14/2020	9.22	11/21/2020	1.96	2/28/2021	1.64
9/22/2018	2.57	12/30/2018	0.26	4/8/2019	799	7/16/2019	4.49	10/23/2019	580	1/30/2020	8.16	5/8/2020	154	8/15/2020	9.46	11/22/2020	1.92	3/1/2021	1.67
9/23/2018	2.4	12/31/2018	0.25	4/9/2019	1010	7/17/2019	4.65	10/24/2019	566	1/31/2020	8.15	5/9/2020	144	8/16/2020	9.08	11/23/2020	1.89	3/2/2021	1.74
9/24/2018	2.26	1/1/2019	0.25	4/10/2019	1600	7/18/2019	4.48	10/25/2019	550	2/1/2020	8.18	5/10/2020	144	8/17/2020	7.7	11/24/2020	1.88	3/3/2021	1.84
9/25/2018	2.16	1/2/2019	0.25	4/11/2019	1260	7/19/2019	4.31	10/26/2019	531	2/2/2020	8.38	5/11/2020	136	8/18/2020	7.39	11/25/2020	1.86	3/4/2021	1.93
9/26/2018	2.23	1/3/2019	0.25	4/12/2019	1090	7/20/2019	4.24	10/27/2019	492	2/3/2020	8.42	5/12/2020	129	8/19/2020	7.7	11/26/2020	1.86	3/5/2021	2.05
9/27/2018	2.2	1/4/2019	0.26	4/13/2019	1010	7/21/2019	4.27	10/28/2019	440	2/4/2020	8.41	5/13/2020	124	8/20/2020	7.91	11/27/2020	1.85	3/6/2021	2.22
9/28/2018	1.92	1/5/2019	0.28	4/14/2019	800	7/22/2019	4.14	10/29/2019	380	2/5/2020	8.41	5/14/2020	126	8/21/2020	7.66	11/28/2020	1.83	3/7/2021	2.4
9/29/2018	1.78	1/6/2019	0.31	4/15/2019	632	7/23/2019	4	10/30/2019	323	2/6/2020	8.4	5/15/2020	124	8/22/2020	7.4	11/29/2020	1.81	3/8/2021	2.6
9/30/2018	1.71	1/7/2019	0.35	4/16/2019	899	7/24/2019	3.9	10/31/2019	244	2/7/2020	8.39	5/16/2020	125	8/23/2020	7.19	11/30/2020	1.8	3/9/2021	2.77
10/1/2018	1.57	1/8/2019	0.41	4/17/2019	1030	7/25/2019	3.8	11/1/2019	210	2/8/2020	8.39	5/17/2020	125	8/24/2020	6.95	12/1/2020	1.79	3/10/2021	2.92
10/2/2018	1.43	1/9/2019	0.45	4/18/2019	1020	7/26/2019	3.72	11/2/2019	178	2/9/2020	8.38	5/18/2020	121	8/25/2020	6.89	12/2/2020	1.78	3/11/2021	3.07
10/3/2018	1.37	1/10/2019	0.48	4/19/2019	1020	7/27/2019	3.57	11/3/2019	156	2/10/2020	8.38	5/19/2020	116	8/26/2020	7.02	12/3/2020	1.76	3/12/2021	3.22
10/4/2018	1.27	1/11/2019	0.51	4/20/2019	911	7/28/2019	4.53	11/4/2019	127	2/11/2020	8.39	5/20/2020	109	8/27/2020	7.41	12/4/2020	1.75	3/13/2021	3.35
10/5/2018	1.42	1/12/2019	0.55	4/21/2019	790	7/29/2019	4.89	11/5/2019	106	2/12/2020	8.34	5/21/2020	103	8/28/2020	7.3	12/5/2020	1.74	3/14/2021	3.49
10/6/2018	1.61	1/13/2019	0.62	4/22/2019	631	7/30/2019	4.34	11/6/2019	88.1	2/13/2020	8.34	5/22/2020	97.4	8/29/2020	6.82	12/6/2020	1.73	3/15/2021	3.62
10/7/2018	1.55	1/14/2019	0.7	4/23/2019	534	7/31/2019	3.55	11/7/2019	75.4	2/14/2020	8.36	5/23/2020	92.5	8/30/2020	6.78	12/7/2020	1.71	3/16/2021	3.77
10/8/2018	1.54	1/15/2019	0.81	4/24/2019	471	8/1/2019	3.34	11/8/2019	70.6	2/15/2020	8.38	5/24/2020	87	8/31/2020	6.74	12/8/2020	1.69	3/17/2021	3.86
10/9/2018	1.51	1/16/2019	0.99	4/25/2019	413	8/2/2019	3.3	11/9/2019	68.1	2/16/2020	8.38	5/25/2020	84.9	9/1/2020	6.53	12/9/2020	1.67	3/18/2021	3.89
10/10/2018	1.6	1/17/2019	1.06	4/26/2019	369	8/3/2019	3.2	11/10/2019	64.8	2/17/2020	8.38	5/26/2020	81.8	9/2/2020	6.07	12/10/2020	1.64	3/19/2021	3.91
10/11/2018	1.51	1/18/2019	1.05	4/27/2019	319	8/4/2019	3.08	11/11/2019	59.2	2/18/2020	8.39	5/27/2020	73.9	9/3/2020	5.89	12/11/2020	1.6	3/20/2021	4.05
10/12/2018	1.41	1/19/2019	1.03	4/28/2019	280	8/5/2019	3	11/12/2019	58.8	2/19/2020	8.43	5/28/2020	74	9/4/2020	5.73	12/12/2020	1.56	3/21/2021	4.24
10/13/2018	1.48	1/20/2019	1.02	4/29/2019	263	8/6/2019	2.87	11/13/2019	58.6	2/20/2020	8.47	5/29/2020	71.5	9/5/2020	5.55	12/13/2020	1.52	3/22/2021	4.29
10/14/2018	1.47	1/21/2019	1.01	4/30/2019	249	8/7/2019	2.89	11/14/2019	56.7	2/21/2020	8.6	5/30/2020	60.5	9/6/2020	11.8	12/14/2020	1.39	3/23/2021	4.38
10/15/2018	1.4	1/22/2019	1	5/1/2019	233	8/8/2019	2.83	11/15/2019	56.2	2/22/2020	8.83	5/31/2020	62.1	9/7/2020	12.6	12/15/2020	1.34	3/24/2021	4.56
10/16/2018	1.33	1/23/2019	0.99	5/2/2019	227	8/9/2019	2.78	11/16/2019	56.4	2/23/2020	8.92	6/1/2020	56.7	9/8/2020	13.1	12/16/2020	1.32	3/25/2021	4.87
10/17/2018	1.23	1/24/2019	0.98	5/3/2019	212	8/10/2019	2.67	11/17/2019	58.5	2/24/2020	8.96	6/2/2020	53.1	9/9/2020	13.2	12/17/2020	1.3	3/26/2021	4.88
10/18/2018	1.16	1/25/2019	0.98	5/4/2019	199	8/11/2019	2.67	11/18/2019	60.5	2/25/2020	8.98	6/3/2020	45.9	9/10/2020	13.7	12/18/2020	1.27	3/27/2021	4.75
10/19/2018	1.13	1/26/2019	0.98	5/5/2019	183	8/12/2019	2.82	11/19/2019	62.1	2/26/2020	8.99	6/4/2020	39.3	9/11/2020	13.6	12/19/2020	1.26	3/28/2021	4.88
10/20/2018	1.02	1/27/2019	0.98	5/6/2019	168	8/13/2019	2.96	11/20/2019	60.6	2/27/2020	9.01	6/5/2020	37.5	9/12/2020	11.9	12/20/2020	1.24	3/29/2021	5.14
10/21/2018	0.93	1/28/2019	0.98	5/7/2019	156	8/14/2019	2.93	11/21/2019	56.7	2/28/2020	9.02	6/6/2020	34	9/13/2020	7.1	12/21/2020	1.22	3/30/2021	4.87
10/22/2018	0.89	1/29/2019	0.98	5/8/2019	143	8/15/2019	2.87	11/22/2019	45.7	2/29/2020	9.04	6/7/2020	37.7	9/14/2020	5.86	12/22/2020	1.21	3/31/2021	4.89
10/23/2018	0.82	1/30/2019	0.97	5/9/2019	132	8/16/2019	2.75	11/23/2019	30.9	3/1/2020	9.13	6/8/2020	137	9/15/2020	5.69	12/23/2020	1.19	4/1/2021	5.1
10/24/2018	0.8	1/31/2019	0.96	5/10/2019	119	8/17/2019	2.7	11/24/2019	31.2	3/2/2020	9.24	6/9/2020	225	9/16/2020	5.4	12/24/2020	1.18	4/2/2021	5.34
10/25/2018	0.79	2/1/2019	0.94	5/11/2019	105	8/18/2019	2.6	11/25/2019	30.7	3/3/2020	9.33	6/10/2020	347	9/17/2020	5.03	12/25/2020	1.18	4/3/2021	5.65
10/26/2018	0.77	2/2/2019	0.93	5/12/2019	94.8	8/19/2019	2.48	11/26/2019	30.8	3/4/2020	9.39	6/11/2020	291	9/18/2020	4.79	12/26/2020	1.18	4/4/2021	6.06
10/27/2018	0.76	2/3/2019	0.92	5/13/2019	85.4	8/20/2019	2.4	11/27/2019	32.6	3/5/2020	9.45	6/12/2020	211	9/19/2020	4.88	12/27/2020	1.18	4/5/2021	6.16
10/28/2018	0.93	2/4/2019	0.89	5/14/2019	77.5	8/21/2019	2.4	11/28/2019	29.4	3/6/2020	9.78	6/13/2020	159	9/20/2020	4.48	12/28/2020	1.18	4/6/2021	5.95
10/29/2018	0.94	2/5/2019	0.86	5/15/2019	73.1	8/22/2019	2.51	11/29/2019	29.4	3/7/2020	10.4	6/14/2020	131	9/21/2020	4.67	12/29/2020	1.18	4/7/2021	5.88
10/30/2018	0.99	2/6/2019	0.83	5/16/2019	69.3	8/23/2019	2.37	11/30/2019	32	3/8/2020	10.8	6/15/2020	102	9/22/2020	5.88	12/30/2020	1.18	4/8/2021	6
10/31/2018	1.04	2/7/2019	0.8	5/17/2019	66.1	8/24/2019	2.22	12/1/2019	27.1	3/9/2020	11.3	6/16/2020	80.6	9/23/2020	5.4	12/31/2020	1.18	4/9/2021	6.14
11/1/2018	1.05	2/8/2019	0.78	5/18/2019	60.6	8/25/2019	2.33	12/2/2019	26.7	3/10/2020	13	6/17/2020	61.7	9/24/2020	4.66	1/1/2021	1.18	4/10/2021	6.1
11/2/2018	1.05	2/9/2019	0.78	5/19/2019	58.1	8/26/2019	2.48	12/3/2019	32.8	3/11/2020	15.3	6/18/2020	52.6	9/25/2020	5.36	1/2/2021	1.18	4/11/2021	6.18
11/3/2018	1.03	2/10/2019	0.77	5/20/2019	52.4	8/27/2019	2.46	12/4/2019	33.2	3/12/2020	15.8	6/19/2020	45.4	9/26/2020	6.41	1/3/2021	1.18	4/12/2021	6.11
11/4/2018	0.98	2/11/2019	0.77	5/21/2019	48.5	8/28/2019	2.43	12/5/2019	30.5	3/13/2020	15.8	6/20/2020	39.3	9/27/2020	6.05	1/4/2021	1.18	4/13/2021	5.54
11/5/2018	0.95	2/12/2019	0.76	5/22/2019	44.2	8/29/2019	2.34	12/6/2019	28.2	3/14/2020	14.1	6/21/2020	32.4	9/28/2020	4.58	1/5/2021	1.18	4/14/2021	5.25
11/6/2018	0.98	2/13/2019	0.75	5/23/2019	44.1	8/30/2019	2.28	12/7/2019	26.4	3/15/2020	12.9	6/22/2020	30	9/29/2020	4.4	1/6/2021	1.18	4/15/2021	5.32

Date	discharge cfs	Date	dis. cfs	Date	dis. cfs	Date	dis. cfs	Date	dis. cfs	Date	dis. cfs	Date	dis. cfs	Date	dis. cfs	Date	dis. cfs	Date	dis. cfs
4/16/2021	5.33	7/24/2021	0.14	10/31/2021	15.1	2/7/2022	0.48	5/17/2022	646	8/24/2022	32.4	12/1/2022	3.85	3/10/2023	3.25	6/17/2023	14.2	9/24/2023	5.31
4/17/2021	5.26	7/25/2021	0.13	11/1/2021	14.4	2/8/2022	0.48	5/18/2022	560	8/25/2022	34.3	12/2/2022	3.74	3/11/2023	3.29	6/18/2023	13.5	9/25/2023	5.38
4/18/2021	5.19	7/26/2021	0.12	11/2/2021	16.3	2/9/2022	0.48	5/19/2022	501	8/26/2022	30.9	12/3/2022	3.64	3/12/2023	3.34	6/19/2023	13.3	9/26/2023	6.51
4/19/2021	4.58	7/27/2021	0.12	11/3/2021	17.5	2/10/2022	0.48	5/20/2022	478	8/27/2022	28.7	12/4/2022	3.49	3/13/2023	3.38	6/20/2023	13	9/27/2023	5.58
4/20/2021	4.64	7/28/2021	0.11	11/4/2021	21	2/11/2022	0.48	5/21/2022	469	8/28/2022	25	12/5/2022	3.22	3/14/2023	3.44	6/21/2023	12.7	9/28/2023	4.93
4/21/2021	4.82	7/29/2021	0.12	11/5/2021	20.5	2/12/2022	0.48	5/22/2022	481	8/29/2022	23.4	12/6/2022	2.87	3/15/2023	3.49	6/22/2023	12.4	9/29/2023	5.6
4/22/2021	4.95	7/30/2021	0.14	11/6/2021	20.6	2/13/2022	0.48	5/23/2022	474	8/30/2022	27.1	12/7/2022	2.82	3/16/2023	3.57	6/23/2023	12.1	9/30/2023	6.18
4/23/2021	4.77	7/31/2021	0.16	11/7/2021	20.6	2/14/2022	0.48	5/24/2022	447	8/31/2022	17.1	12/8/2022	2.82	3/17/2023	3.65	6/24/2023	12.8	10/1/2023	6.24
4/24/2021	4.94	8/1/2021	0.17	11/8/2021	20.5	2/15/2022	0.48	5/25/2022	398	9/1/2022	12.1	12/9/2022	2.82	3/18/2023	3.72	6/25/2023	23.6	10/2/2023	5.76
4/25/2021	4.6	8/2/2021	0.18	11/9/2021	20.3	2/16/2022	0.48	5/26/2022	355	9/2/2022	11.3	12/10/2022	2.82	3/19/2023	3.79	6/26/2023	62	10/3/2023	5.63
4/26/2021	4.48	8/3/2021	0.18	11/10/2021	20.7	2/17/2022	0.48	5/27/2022	324	9/3/2022	11.4	12/11/2022	2.82	3/20/2023	3.83	6/27/2023	155	10/4/2023	4.64
4/27/2021	4.72	8/4/2021	0.18	11/11/2021	20.7	2/18/2022	0.48	5/28/2022	293	9/4/2022	11	12/12/2022	2.82	3/21/2023	3.84	6/28/2023	316	10/5/2023	4.39
4/28/2021	4.68	8/5/2021	0.18	11/12/2021	20.7	2/19/2022	0.47	5/29/2022	268	9/5/2022	7.97	12/13/2022	2.82	3/22/2023	3.84	6/29/2023	336	10/6/2023	5.21
4/29/2021	4.48	8/6/2021	0.18	11/13/2021	20.3	2/20/2022	0.47	5/30/2022	247	9/6/2022	7.21	12/14/2022	2.82	3/23/2023	3.84	6/30/2023	308	10/7/2023	6.46
4/30/2021	5.37	8/7/2021	0.18	11/14/2021	19.5	2/21/2022	0.47	5/31/2022	264	9/7/2022	7	12/15/2022	2.82	3/24/2023	6.26	7/1/2023	275	10/8/2023	5.18
5/1/2021	5.8	8/8/2021	0.17	11/15/2021	20.8	2/22/2022	0.47	6/1/2022	350	9/8/2022	6.92	12/16/2022	2.82	3/25/2023	25.4	7/2/2023	243	10/9/2023	4.29
5/2/2021	4.97	8/9/2021	0.16	11/16/2021	20.7	2/23/2022	0.47	6/2/2022	439	9/9/2022	6.64	12/17/2022	2.82	3/26/2023	32.9	7/3/2023	203	10/10/2023	3.87
5/3/2021	4.98	8/10/2021	0.16	11/17/2021	20.3	2/24/2022	0.47	6/3/2022	504	9/10/2022	6.83	12/18/2022	2.82	3/27/2023	31.3	7/4/2023	148	10/11/2023	3.66
5/4/2021	4.68	8/11/2021	0.15	11/18/2021	17.7	2/25/2022	0.47	6/4/2022	530	9/11/2022	6.21	12/19/2022	2.82	3/28/2023	33.5	7/5/2023	108	10/12/2023	3.45
5/5/2021	4.67	8/12/2021	0.15	11/19/2021	17	2/26/2022	0.47	6/5/2022	490	9/12/2022	5.81	12/20/2022	2.82	3/29/2023	36	7/6/2023	69.9	10/13/2023	3.17
5/6/2021	4.59	8/13/2021	0.14	11/20/2021	16.6	2/27/2022	0.47	6/6/2022	396	9/13/2022	5.11	12/21/2022	2.82	3/30/2023	38.3	7/7/2023	47.1	10/14/2023	3.08
5/7/2021	4.56	8/14/2021	0.13	11/21/2021	12.3	2/28/2022	0.47	6/7/2022	316	9/14/2022	4.5	12/22/2022	2.82	3/31/2023	32.7	7/8/2023	37.9	10/15/2023	3.2
5/8/2021	4.6	8/15/2021	0.13	11/22/2021	9.45	3/1/2022	0.47	6/8/2022	261	9/15/2022	4.74	12/23/2022	2.82	4/1/2023	24.7	7/9/2023	25.9	10/16/2023	3.24
5/9/2021	4.62	8/16/2021	0.12	11/23/2021	9.33	3/2/2022	0.47	6/9/2022	217	9/16/2022	5.46	12/24/2022	2.82	4/2/2023	19	7/10/2023	21.5	10/17/2023	3.26
5/10/2021	4.64	8/17/2021	0.12	11/24/2021	8.74	3/3/2022	0.47	6/10/2022	186	9/17/2022	5.37	12/25/2022	2.82	4/3/2023	15.9	7/11/2023	18.1	10/18/2023	3.18
5/11/2021	4.49	8/18/2021	0.08	11/25/2021	7.37	3/4/2022	0.47	6/11/2022	166	9/18/2022	6.69	12/26/2022	2.82	4/4/2023	13	7/12/2023	15.5	10/19/2023	3.04
5/12/2021	4.55	8/19/2021	0.05	11/26/2021	6.83	3/5/2022	0.48	6/12/2022	153	9/19/2022	5.9	12/27/2022	2.82	4/5/2023	9.77	7/13/2023	13.3	10/20/2023	3.1
5/13/2021	4.45	8/20/2021	0.07	11/27/2021	6.55	3/6/2022	0.48	6/13/2022	140	9/20/2022	5.34	12/28/2022	2.82	4/6/2023	7.68	7/14/2023	12.3	10/21/2023	3.03
5/14/2021	4.3	8/21/2021	0.09	11/28/2021	6.44	3/7/2022	0.48	6/14/2022	136	9/21/2022	4.77	12/29/2022	2.82	4/7/2023	13.1	7/15/2023	11.6	10/22/2023	3.55
5/15/2021	4.32	8/22/2021	0.07	11/29/2021	6.39	3/8/2022	0.48	6/15/2022	163	9/22/2022	6.01	12/30/2022	2.82	4/8/2023	13.2	7/16/2023	10.7	10/23/2023	4.8
5/16/2021	4.32	8/23/2021	0.05	11/30/2021	6.3	3/9/2022	0.48	6/16/2022	414	9/23/2022	7.1	12/31/2022	2.82	4/9/2023	7.99	7/17/2023	9.93	10/24/2023	3.9
5/17/2021	4.2	8/24/2021	0.03	12/1/2021	6.23	3/10/2022	0.48	6/17/2022	414	9/24/2022	5.51	1/1/2023	2.82	4/10/2023	7.52	7/18/2023	9.18	10/25/2023	3.51
5/18/2021	4.12	8/25/2021	0.02	12/2/2021	6.2	3/11/2022	0.48	6/18/2022	409	9/25/2022	4.52	1/2/2023	2.82	4/11/2023	9.33	7/19/2023	8.88	10/26/2023	3.65
5/19/2021	4.19	8/26/2021	0.01	12/3/2021	6.16	3/12/2022	0.48	6/19/2022	401	9/26/2022	4.09	1/3/2023	2.83	4/12/2023	13	7/20/2023	8	10/27/2023	3.57
5/20/2021	6.61	8/27/2021	0.02	12/4/2021	5.93	3/13/2022	0.48	6/20/2022	367	9/27/2022	4.88	1/4/2023	2.83	4/13/2023	50.5	7/21/2023	7.64	10/28/2023	3.58
5/21/2021	5.51	8/28/2021	0.02	12/5/2021	5.63	3/14/2022	0.48	6/21/2022	295	9/28/2022	4.47	1/5/2023	2.83	4/14/2023	328	7/22/2023	7.79	10/29/2023	3.5
5/22/2021	5.62	8/29/2021	0.03	12/6/2021	5.21	3/15/2022	0.48	6/22/2022	228	9/29/2022	3.99	1/6/2023	2.83	4/15/2023	633	7/23/2023	7.28	10/30/2023	3.41
5/23/2021	5.04	8/30/2021	0.02	12/7/2021	4.96	3/16/2022	0.83	6/23/2022	181	9/30/2022	3.75	1/7/2023	2.83	4/16/2023	846	7/24/2023	7	10/31/2023	3.36
5/24/2021	5.19	8/31/2021	0.02	12/8/2021	4.87	3/17/2022	1.82	6/24/2022	157	10/1/2022	3.63	1/8/2023	2.83	4/17/2023	1010	7/25/2023	7.31	11/1/2023	3.33
5/25/2021	5.73	9/1/2021	0.02	12/9/2021	4.85	3/18/2022	3.06	6/25/2022	564	10/2/2022	3.51	1/9/2023	2.83	4/18/2023	1270	7/26/2023	9.23	11/2/2023	3.52
5/26/2021	5.17	9/2/2021	0.01	12/10/2021	4.84	3/19/2022	26.5	6/26/2022	887	10/3/2022	3.47	1/10/2023	2.83	4/19/2023	1340	7/27/2023	11.7	11/3/2023	4.17
5/27/2021	5.32	9/3/2021	0.02	12/11/2021	4.84	3/20/2022	73.2	6/27/2022	552	10/4/2022	3.39	1/11/2023	2.84	4/20/2023	1290	7/28/2023	10.9	11/4/2023	5.7
5/28/2021	5.39	9/4/2021	0.02	12/12/2021	4.84	3/21/2022	221	6/28/2022	464	10/5/2022	3.64	1/12/2023	2.84	4/21/2023	1230	7/29/2023	9.42	11/5/2023	6.34
5/29/2021	4.91	9/5/2021	0.02	12/13/2021	4.84	3/22/2022	615	6/29/2022	412	10/6/2022	3.88	1/13/2023	2.84	4/22/2023	1180	7/30/2023	8.39	11/6/2023	6.26
5/30/2021	4.75	9/6/2021	0.02	12/14/2021	4.84	3/23/2022	781	6/30/2022	375	10/7/2022	4.65	1/14/2023	2.84	4/23/2023	1130	7/31/2023	7.8	11/7/2023	6.22
5/31/2021	4.41	9/7/2021	0.02	12/15/2021	4.84	3/24/2022	899	7/1/2022	321	10/8/2022	5.6	1/15/2023	2.84	4/24/2023	1000	8/1/2023	7.77	11/8/2023	6.18
6/1/2021	4.36	9/8/2021	0.02	12/16/2021	4.75	3/25/2022	1050	7/2/2022	261	10/9/2022	4.76	1/16/2023	2.84	4/25/2023	822	8/2/2023	7.77	11/9/2023	6.15
6/2/2021	4.32	9/9/2021	0.01	12/17/2021	4.62	3/26/2022	1010	7/3/2022	212	10/10/2022	5.18	1/17/2023	2.84	4/26/2023	805	8/3/2023	7.9	11/10/2023	6.11
6/3/2021	4.15	9/10/2021	0.01	12/18/2021	4.48	3/27/2022	818	7/4/2022	179	10/11/2022	4.34	1/18/2023	2.84	4/27/2023	938	8/4/2023	8.03	11/11/2023	6.08
6/4/2021	4.07	9/11/2021	0	12/19/2021	4.28	3/28/2022	798	7/5/2022	154	10/12/2022	3.95	1/19/2023	2.84	4/28/2023	1250	8/5/2023	7.96	11/12/2023	6.05
6/5/2021	3.78	9/12/2021	0	12/20/2021	4.11	3/29/2022	788	7/6/2022	140	10/13/2022	4.58	1/20/2023	2.84	4/29/2023	1220	8/6/2023	7.86	11/13/2023	6.03
6/6/2021	3.41	9/13/2021	0	12/21/2021	3.94	3/30/2022	693	7/7/2022	128	10/14/2022	5.65	1/21/2023	2.85	4/30/2023	1220	8/7/2023	7.91	11/14/2023	6
6/7/2021	2.73	9/14/2021	0.01	12/22/2021	3.77	3/31/2022	496	7/8/2022	113	10/15/2022	4.92	1/22/2023	2.85	5/1/2023	1220	8/8/2023	8.14	11/15/2023	5.96
6/8/2021	2.32	9/15/2021	0.01	12/23/2021	3.59	4/1/2022	457	7/9/2022	102	10/16/2022	3.94	1/23/2023	2.85	5/2/2023	1190	8/9/2023	7.88	11/16/2023	5.93
6/9/2021	2.14	9/16/2021	0.06	12/24/2021	3.4	4/2/2022	446	7/10/2022	101	10/17/2022	3.47	1/24/2023	2.85	5/3/2023	1160	8/10/2023	7.43	11/17/2023	5.9
6/10/2021	2.05	9/17/2021	0.06	12/25/2021	3.19	4/3/2022	553	7/11/2022	97.3	10/18/2022	3.37	1/25/2023	2.85	5/4/2023	1140	8/11/2023	7.77	11/18/2023	5.88
6/11/2021	2.41	9/18/2021	0.06	12/26/2021	2.97	4/4/2022	542	7/12/2022	88.9	10/19/2022	3.53	1/26/2023	2.85	5/5/2023	1080	8/12/2023	7.32	11/19/2023	5.86
6/12/2021	2.71	9/19/2021	0.06	12/27/2021	2.76	4/5/2022	516	7/13/2022	73	10/20/2022	3.54	1/27/2023	2.85	5/6/2023	943	8/13/2023	7.29	11/20/2023	5.84
6/13/2021	2.43	9/20/2021	0.08	12/28/2021	2.55	4/6/2022	626	7/14/2022	63.4	10/21/2022	3.77	1/28/2023	2.85	5/7/2023	813	8/14/2023	7.56	11/21/2023	5.83
6/14/2021	2.29	9/21/2021	0.08	12/29/2021	2.37	4/7/2022	620	7/15/2022	61.3	10/22/2022	4.1	1/29/2023	2.85	5/8/2023	728	8/15/2023	8.21	11/22/2023	5.8
6/15/2021	2.18	9/22/2021	0.08	12/30/2021	2.24	4/8/2022	565	7/16/2022	63.3	10/23/2022	4.11	1/30/2023	2.86	5/9/2023	660	8/16/2023	8.28	11/23/2023	5.79
6/16/2021	2.05	9/23/2021	0.07	12/31/2021	2.09	4/9/2022	483	7/17/2022	64.2	10/24/2022	4.6	1/31/2023	2.87	5/10/2023	599	8/17/2023	8.88	11/24/2023	5.77
6/17/2021	1.93	9/24/2021	0.07	1/1/2022	1.92	4/10/2022	460	7/18/2022	58.2	10/25/2022	4.46	2/1/2023	2.87	5/11/2023	548	8/18/2023	10.1	11/25/2023	5.75
6/18/2021	1.81	9/25/2021	0.06	1/2/2022	1.77	4/11/2022	566	7/19/2022	49.1	10/26/2022	6.16	2/2/2023	2.87	5/12/2023	499	8/19/2023	8.67	11/26/2023	5.73
6/19/2021	1.71	9/26/2021	0.06	1/3/2022	1.67	4/12/2022	578	7/20/2022	59.3	10/27/2022	5.76	2/3/2023	2.88	5/13/2023	451	8/20/2023	7.23	11/27/2023	5.7
6/20/2021	1.65	9/27/2021	0.05	1/4/2022	1.56	4/13/2022	668	7/21/2022	54.6	10/28/2022	4.73	2/4/2023	2.88	5/14/2023	408	8/21/2023	6.94	11/28/2023	5.68
6/21/2021	1.57	9/28/2021	0.05	1/5/2022	1.44	4/14/2022	585	7/22/2022	50.3	10/29/2022	4.55	2/5/2023	2.88	5/15/2023	371	8/22/2023	6.48	11/29/2023	5.66
6/22/2021	1.47	9/29/2021	0.04	1/6/2022	1.32	4/15/2022	429	7/23/2022	58.1	10/30/2022	6.07	2/6/2023	2.89	5/16/2023	330	8/23/2023	6.32	11/30/2023	5.62
6/23/2021	1.38	9/30/2021	0.04	1/7/2022	1.23	4/16/2022	342	7/24/2022	80.4	10/31/2022	5.56	2/7/2023	2.89	5/17/2023	297	8/24/2023	6.01	12/1/2023	5.59
6/24/2021	1.3	10/1/2021	0.03	1/8/2022	1.16	4/17/2022	260	7/25/2022	108	11/1/2022	5.54	2/8/2023	2.89	5/18/2023	267	8/25/2023	5.57	12/2/2023	5.57
6/25/2021	1.25	10/2/2021	0.03	1/9/2022	1.09	4/18/2022	235	7/26/2022	112	11/2/2022	6.48	2/9/2023	2.9	5/19/2023	244	8/26/2023	4.9	12/3/2023	5.54
6/26/2021	1.21	10/3/2021	0.03	1/10/2022	1	4/19/2022	203	7/27/2022	116	11/3/2022	5.86	2/10/2023	2.91	5/20/2023	222	8/27/2023	4.61	12/4/2023	5.51
6/27/2021	1.17	10/4/2021	0.03	1/11/2022	0.92	4/20/2022	188	7/28/2022	112	11/4/2022	5.85	2/11/2023	2.91	5/21/2023	203	8/28/2023	5.43	12/5/2023	5.48
6/28/2021	1.12	10/5/2021	0.02	1/12/2022	0.84	4/21/2022	175	7/29/2022	100	11/5/2022	5.85	2/12/2023	2.92	5/22/2023	178	8/29/2023	6.06	12/6/2023	5.46
6/29/2021	1.07	10/6/2021	0.02	1/13/2022	0.79	4/22/2022	204	7/30/2022	85.5	11/6/2022	5.83	2/13/2023	2.92	5/23/2023	160	8/30/2023	4.77	12/7/2023	5.44
6/30/2021	1	10/7/2021	0.01	1/14/2022	0.74	4/23/2022	639	7/31/2022	71.6	11/7/2022	5.8	2/14/2023	2.93	5/24/2023	142	8/31/2023	4.06	12/8/2023	5.42
7/1/2021	0.94	10/8/2021	0.03	1/15/2022	0.69	4/24/2022	1050	8/1/2022	65.4	11/8/2022	5.65	2/15/2023	2.94	5/25/2023	133	9/1/2023	3.56	12/9/2023	5.39
7/2/2021	0.89	10/9/2021	0.11	1/16/2022	0.64	4/25/2022	1460	8/2/2022	55.7	11/9/2022	5.12	2/16/2023	2.94	5/26/2023	124	9/2/2023	3.16	12/10/2023	5.37
7/3/2021	0.84	10/10/2021	0.47	1/17/2022	0.61	4/26/2022	1440	8/3/2022	47.3	11/10/2022	4.41	2/17/2023	2.94	5/27/2023	111	9/3/2023	2.94	12/11/2023	5.35
7/4/2021	0.79	10/11/2021	0.64	1/18/2022	0.58	4/27/2022	1380	8/4/2022	47.3	11/11/2022	4.37	2/18/2023	2.95	5/28/2023	98.6	9/4/2023	4.89	12/12/2023	5.32
7/5/2021	0.73	10/12/2021	1.07	1/19/2022	0.57	4/28/2022	1340	8/5/2022	35.4	11/12/2022	5.28	2/19/2023	2.96	5/29/2023	85.3	9/5/2023	6.98	12/13/2023	5.29
7/6/2021	0.67	10/13/2021	2.21	1/20/2022	0.55	4/29/2022	1310	8/6/2022	30.2	11/13/2022	5.47	2/20/2023	2.97	5/30/2023	73.8	9/6/2023	7.77	12/14/2023	5.27
7/7/2021	0.62	10/14/2021	7.91	1/21/2022	0.54	4/30/2022	1320	8/7/2022	29.7	11/14/2022	5.5	2/21/2023	2.98	5/31/2023	67.1	9/7/2023	7.27	12/15/2023	5.26
7/8/2021	0.52	10/15/2021	10.3	1/22/2022	0.52	5/1/2022	1360	8/8/2022	24.8	11/15/2022	5.5	2/22/2023	2.99	6/1/2023	60.8	9/8/2023	6.14	12/16/2023	5.23
7/9/2021	0.39	10/16/2021	27.5	1/23/2022	0.5	5/2/2022	1400	8/9/2022	22.3	11/16/2022	5.5	2/23/2023	3	6/2/2023	51.4	9/9/2023	5.34	12/17/2023	5.22
7/10/2021	0.31	10/17/2021	79.4	1/24/2022	0.49	5/3/2022	1360	8/10/2022	20.5	11/17/2022	5.5	2/24/2023	3.01	6/3/2023	42.3	9/10/2023	4.75	12/18/2023	5.19
7/11/2021	0.25	10/18/2021	58.5	1/25/2022	0.48	5/4/2022	1280	8/11/2022	17.4	11/18/2022	5.5	2/25/2023	3.02	6/4/2023	36	9/11/2023	4.5	12/19/2023	5.16
7/12/2021	0.2	10/19/2021	38.9	1/26/2022	0.48	5/5/2022	1200	8/12/2022	15.8	11/19/2022	5.46	2/26/2023	3.03	6/5/2023	30.5	9/12/2023	4.13	12/20/2023	5.14
7/13/2021	0.17	10/20/2021	27.9	1/27/2022	0.48	5/6/2022	1130	8/13/2022	14.7	11/20/2022	5.32	2/27/2023	3.04	6/6/2023	32.4	9/13/2023	4.28	12/21/2023	5.12
7/14/2021	0.16	10/21/2021	19.1	1/28/2022	0.49	5/7/2022	1070	8/14/2022	13.9	11/21/2022	5.14	2/28/2023	3.05	6/7/2023	28.6	9/14/2023	4.64	12/22/2023	5.09
7/15/2021	0.15	10/22/2021	15.4	1/29/2022	0.49	5/8/2022	1030	8/15/2022	13.1	11/22/2022	5.02	3/1/2023	3.06	6/8/2023	27.1	9/15/2023	4.52	12/23/2023	5.07
7/16/2021	0.14	10/23/2021	13.1	1/30/2022	0.49	5/9/2022	1000	8/16/2022	12.8	11/23/2022	5	3/2/2023	3.08	6/9/2023	26.4	9/16/2023	4.31	12/24/2023	5.03
7/17/2021	0.14	10/24/2021	11.9	1/31/2022	0.49	5/10/2022	997	8/17/2022	12.4	11/24/2022	4.99	3/3/2023	3.09	6/10/2023	26	9/17/2023	5.6	12/25/2023	5.01
7/18/2021	0.13	10/25/2021	10.2	2/1/2022	0.49	5/11/2022	960	8/18/2022	12.8	11/25/2022	4.99	3/4/2023	3.11	6/11/2023	24.5	9/18/2023	5.34	12/26/2023	4.98
7/19/2021	0.14	10/26/2021	9.37	2/2/2022	0.49	5/12/2022	945	8/19/2022	13.7	11/26/2022	4.99	3/5/2023	3.12	6/12/2023	22.9	9/19/2023	4.56	12/27/2023	4.88
7/20/2021	0.16	10/27/2021	9.84	2/3/2022	0.49	5/13/2022	933	8/20/2022	16.7	11/27/2022	4.98	3/6/2023	3.14	6/13/2023	21.6	9/20/2023	4.51	12/28/2023	4.63
7/21/2021	0.16	10/28/2021	9.98	2/4/2022	0.48	5/14/2022	918	8/21/2022	32.9	11/28/2022	4.96	3/7/2023	3.16	6/14/2023	19.8	9/21/2023	4.78	12/29/2023	4.4
7/22/2021	0.15	10/29/2021	10.9	2/5/2022	0.48	5/15/2022	848	8/22/2022	35.4	11/29/2022	4.74	3/8/2023	3.18	6/15/2023	17.8	9/22/2023	5.39	12/30/2023	4.1
7/23/2021	0.15	10/30/2021	18.8	2/6/2022	0.48	5/16/2022	728	8/23/2022	32.7	11/30/2022	4.18	3/9/2023	3.21	6/16/2023	14.8	9/23/2023	4.97	12/31/2023	3.75

References

Chu, X., Yang, J., Zhang, J., Chi, Y. (2010). An improved method for watershed delineation and computation of surface depression storage. In: Watershed Management 2010. American Society of Civil Engineers, Reston, VA, pp. 1113–1122.

Khanaum, M. M., Qi, T., & Chu, X. (2025). Dynamic Partial Contributing Area (DPCA) approach: Improved hydrologic modeling for depression-dominated watersheds. *Journal of Hydrology*, *658*, 133077. https://doi.org/10.1016/j.jhydrol.2025.133077
